# Dysregulated m^6^A via compensatory arginine methylation primes premalignancy in metabolic dysfunction–associated steatotic liver disease

**DOI:** 10.1126/sciadv.aec5094

**Published:** 2026-09-09

**Authors:** Geun-Woo D. Kim, Dahee Choi, Soo-Young Kim, Haengdueng Jeong, Geon Kang, So Jung Eom, Sangkyeong Eom, Jinyoung Park, Hye-Sook Lee, Insook Yang, Je Kyung Sung, Ki Taek Nam, Young Nyun Park, Sung Wook Chi, Seung-Hoi Koo

**Affiliations:** ^1^ Division of Life Sciences, Korea University, Seoul 02841, Korea.; ^2^ KU-KIST Graduate School of Converging Science and Technology, Korea University, Seoul 02841, Korea.; ^3^ Department of Molecular and Life Science, Hanyang University, Ansan, Korea.; ^4^ Yonsei Biomedical Research Institute, Yonsei University College of Medicine, Seoul 03760, Korea.; ^5^ Korea Model Animal Priority Center, Seoul National University, Seoul 08826, Korea.; ^6^ Department of Biomedical Sciences, Brain Korea 21 Project for Medical Science, Yonsei University College of Medicine, Seoul 03760, Korea.; ^7^ Department of Pathology, Graduate School of Medical Science, Brain Korea 21 Project for Medical Science, Yonsei University College of Medicine, Seoul 03760, Korea.; ^8^ National Research Laboratory for Convergence Degradation Biology, Korea University, Seoul 02841, Korea.; ^9^ Medicinal Materials Research Center, Biomedical Research Division, Korea Institute of Science and Technology (KIST), Seoul 02792, Korea.

## Abstract

Hepatocellular carcinoma (HCC) originates from premalignant disease-associated hepatocytes (daHeps) that emerge during the progression of metabolic dysfunction–associated steatotic liver disease (MASLD) to metabolic dysfunction–associated steatohepatitis (MASH). As daHeps are compensatorily primed by metabolic stress, we reproduced the accelerated progression of MASLD-associated HCC in mice by phenocopying the decreased expression of a metabolic regulator, protein arginine methyltransferase 1 (PRMT1). In *Prmt1* liver-specific knockout (LKO), m^6^A-mediated changes in mRNA stability reprogram the transcriptome via paralog compensation; increased PRMT6 activates m^6^A methyltransferases by inducing asymmetric arginine dimethylation of METTL3. This event enhances global m^6^A deposition that leads to the down-regulation of *Keap1*, which would trigger the NRF2 axis, promoting premalignancy; under diet- and chemical-induced stress, the incidence of steatohepatitic HCC was increased, clinically correlating with the PRMT-METTL3-NRF2 pathway. Together, we propose that compensatory arginine methylation primes MASLD-associated HCC by modulating m^6^A-mediated transcriptome and NRF2 regulatory pathways as adaptive defenses against metabolic and oxidative stress.

## INTRODUCTION

Metabolic dysfunction–associated steatotic liver disease (MASLD), formerly known as nonalcoholic fatty liver disease (NAFLD), has emerged as one of the major health threats in recent years (*1*–*4*). Diet-induced hepatic steatosis is associated with various metabolic disorders and, if left untreated, can develop into hepatic fibrosis, metabolic dysfunction–associated steatohepatitis (MASH), and, ultimately, hepatocellular carcinoma (HCC) (*5*).

HCC develops from differentiated hepatocytes that proliferate to compensate for damaged liver during the progression of MASLD to MASH (*6*, *7*). Chronic injury under metabolic stress mainly induces hepatocyte senescence during these fibrotic stages, initially conferring tumor suppressive activity (*8*, *9*). Senescent hepatocytes are characterized by activation of p53 and subsequent expression of gluconeogenic enzyme fructose-1,6-bisphosphatase 1 (FBP1), which also functions as a direct inhibitor of AKT proto-oncogene (*10*, *11*). Recently, a disease-associated hepatocyte (daHep) subpopulation, which resembles mouse HCC progenitor cells, has been identified as conferring premalignant cell states, driven by ongoing metabolic stress that activates nuclear factor erythroid–derived 2–related factor (NRF2) to reverse senescence and support proliferation (*7*, *10*, *12*). Aberrant activation of NRF2 induces phosphorylation-dependent FBP1 degradation, removes the brake on AKT activation, and accelerates p53 degradation to afford somatic mutations, premalignant changes that may be primed by global RNA dysregulation altering metabolic signaling as a compensatory defense against metabolic and oxidative stress.

Critical metabolic regulators, protein arginine methyltransferases (PRMTs), comprise 11 family members that promote methylation of arginine residues on both histone proteins and cellular proteins (*13*, *14*). Among the type I PRMTs, which confer asymmetric dimethyl arginine and generally promote transcription, PRMT1 is responsible for most of asymmetric arginine dimethylation found in mammalian cell (*13*, *15*–*19*). In addition to its role in cardiac myocytes, skeletal muscles, and adipocytes (*20*–*22*), the role of PRMT1 in the liver has been delineated recently. In the liver, PRMT1 functions as a positive regulator of hepatic gluconeogenesis via modification of FoxO1 proteins (*23*, *24*) and protects alcoholic fatty liver disease (AFLD) by regulating hepatic nuclear factor 4 (HNF4) (*25*), highlighting its prominent role in hepatic metabolic homeostasis.

The *N*^6^-methyladenosine (m^6^A) is the most prevalent mRNA modification, regulating RNA stability, splicing, transport, and translation to control transcriptome profile and expression (*26*). m^6^A modification frequently occurs near stop codon and 3′ untranslated region (3′UTR), and long exon (*26*). The m^6^A methyltransferase complex consists of methyltransferase-like 3 (METTL3), METTL14, and Wilms tumor 1–associated protein (WTAP). METTL14 functions in substrate recognition (RRACH; R means G or A, and H means A, C, or U) and activation of the main catalytic enzyme METTL3 (*27*), and WTAP is required for nuclear localization as a regulatory subunit (*28*). m^6^A modification can be reversed by demethylases, fat mass and obesity-associated protein (FTO) (*29*), and AlkB homolog 5 (ALKBH5) (*30*). Dysregulation of m^6^A modification has been linked to metabolic disorders and hepatocarcinogenesis (*31*, *32*), with context-dependent roles in hepatic diseases. Chronic deletion of hepatic METTL3 causes lethal hepatic malformations (*33*, *34*), whereas postnatal conditional loss is benign (*33*). Likewise, m^6^A modification has been reported to both exacerbate (*35*, *36*) and suppress (*37*) MASLD. Although PRMT1-mediated arginine methylation of METTL14 has been detected during differentiation of mouse embryonic stem cells (*38*, *39*), the pathological relevance of PRMT paralogs and m^6^A mRNA modification in metabolic dysfunction and tumorigenesis remains poorly understood.

Because PRMT1 metabolically functions in gluconeogenesis like FBP1 (*10*, *11*, *23*, *24*), we initially examined expression of PRMT1 and observed specific reduction in the premalignant daHep subpopulation. To phenocopy this, we generated liver-specific *Prmt1* knockout (*Prmt1* LKO) mice and observed accelerated MASLD-associated HCC progression. *Prmt1* LKO mice showed progression of fatty liver disease under high-fat diet (HFD), with transcriptome alterations in lipid metabolism and redox-responsive genes reflecting activation of the NRF2 pathway. Close investigation revealed that the depletion of *Prmt1* resulted in compensatory increase in PRMT6, which induced asymmetric arginine dimethylation of METTL3 and resultant m^6^A modification that destabilized target mRNAs including *Keap1*. This would consequently activate NRF2, reduce FBP1, and decrease p53 (*10*), thereby priming premalignancy for MASLD-associated HCC. Such m^6^A-mediated mRNA regulation correlated with lower *PRMT1* and higher *PRMT6* in protumorigenic hepatocytes from patients with MASLD-associated HCC. Consistent with this protumorigenic PRMT-METTL3-NRF2-FBP1 axis, *Prmt1* LKO mice showed enhanced HCC progression under HFD and chemically induced tumorigenic conditions. These data collectively demonstrate that dysregulation of m^6^A deposition, mediated by compensatory arginine methylation, is critical for progression to MASLD-associated HCC by priming premalignant states through NRF2 activation as an adaptive defense against metabolic and oxidative stress.

## RESULTS

### Liver-specific depletion of *Prmt1* phenocopies MASLD to MASH in mice

**Fig. 1.**
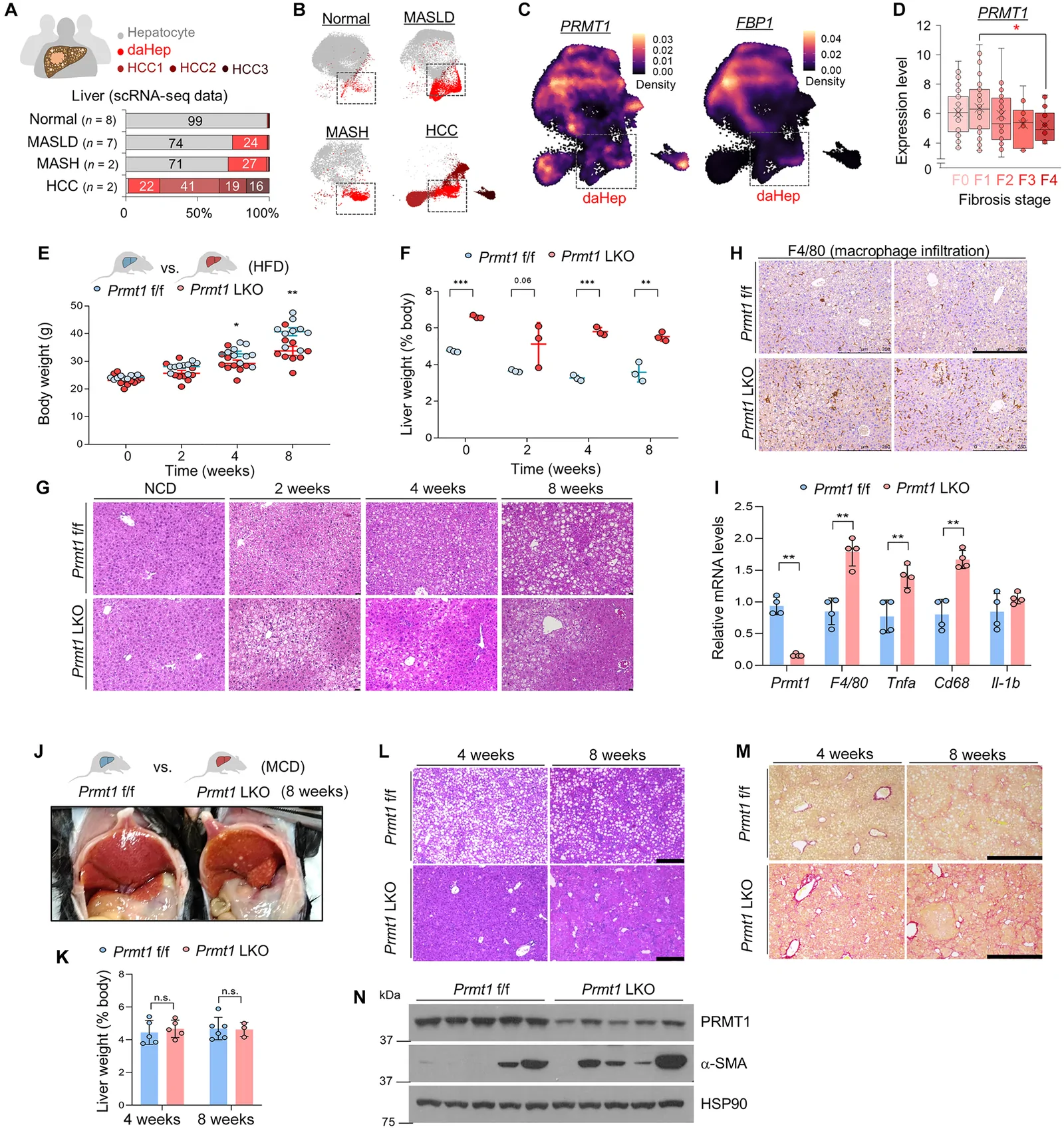
Hepatic PRMT1 deters the progression of diet-induced MASLD. (**A** and **B**) Distribution of hepatocyte populations in patients (A), derived from scRNA-seq data; UMAP plots (B); daHep population, highlighted by a dotted rectangle. (**C**) Heatmap density plots for expression. (**D**) Average expression levels of *PRMT1* in patients with MASH [*n* = 112; GSE162694; (*40*)]; box plots (lines represent the median and the first and third quartiles, whiskers denote the minima and maxima, and “x” marks the mean). (**E**) Changes of body weight of *Prmt1* f/f mice (*n* = 9) and *Prmt1* LKO mice (*n* = 9) upon HFD feeding. (**F**) Changes of liver weight relative to body weight of *Prmt1* f/f mice (*n* = 3) and *Prmt1* LKO mice (*n* = 3) upon HFD feeding. (**G**) Paraffin-embedded sections of liver tissues from 8-week HFD-fed mice; hematoxylin and eosin (H&E) staining (*n* = 3 independent mice per group). Scale bars, 250 μm (×100). (**H**) Histological staining of F4/80 (brown; *n* = 3 independent mice per group). Scale bars, 250 μm (×200). (**I**) The mRNA levels of macrophage-specific genes; quantitative reverse transcription polymerase chain reaction (qRT-PCR) in the liver tissues (*n* = 4 per group). (**J**) A representative picture showing morphologies of livers from *Prmt1* f/f mice or *Prmt1* LKO mice following 8-week MCD diet. (**K**) Relative liver weight after 4-week MCD diet (left; *n* = 5 for *Prmt1* f/f mice and *n* = 5 for *Prmt1* LKO mice) or 8-week MCD diet (right; *n* = 6 for *Prmt1* f/f mice and *n* = 3 for *Prmt1* LKO mice). (**L** and **M**) H&E staining (L) and Picrosirius Red staining (M). Data represent three independent experiments (*n* = 3 mice per group). Scale bars, 100 μm (×50). (**N**) Western blot analysis in the liver tissues from either *Prmt1* f/f mice or *Prmt1* LKO mice under 4-week MCD diet. Values represent means ± SEM. *P < 0.05, **P < 0.01, and ***P < 0.001. n.s., not significant.

By compiling single-nucleus RNA-sequencing (snRNA-seq) data obtained at different stepwise stages of MASLD-associated HCC, we initially confirmed development of daHep subpopulation during progression of MASLD (24%) to MASH (27%), followed by transformation into HCC clusters thought to represent HCC progenitors (Fig. 1, A and B). In line with the recent study (*10*), daHep population in patients also showed reduced expression of fructose 1,6-bisphosphatase (*FBP1*), a critical regulator of gluconeogenesis (Fig. 1C, right). Because PRMT1 also regulates hepatic gluconeogenesis (*23*, *24*), we examined its expression and found significant down-regulation in daHeps (Fig. 1C, left). Notably, similar to *FBP1*, *PRMT1* expression exhibited a progressive decline with increasing fibrosis stage in patients with MASH (Fig. 1D and fig. S1A) (*40*, *41*), prompting us to further investigate the effect of hepatic depletion of *Prmt1* in MASLD and/or MASLD-associated HCC by generation of *Prmt1* LKO mice (fig. S1, B and C). Under a normal chow diet, chronic depletion of hepatic *Prmt1* did not perturb glucose metabolism as shown by the measurement of blood glucose levels as well as the result of glucose tolerance test (GTT) and insulin tolerance test (ITT; fig. S1, D and E). On the other hand, we observed an increase in hepatic triacylglycerol (TG) levels in *Prmt1* LKO mice compared with those in *Prmt1* wild-type (WT; *Prmt1* f/f) mice without significant changes in serum TG levels (fig. S1, F and G), suggesting that depletion of hepatic *Prmt1* perturbs lipid metabolism.

To instigate the MASLD phenotypes in mice, we fed mice HFD for up to 8 weeks. *Prmt1* LKO mice showed resistance to body weight gain upon HFD feeding compared with the control (Fig. 1E). In spite of the reduction in body weight, liver weight remained higher in *Prmt1* LKO mice compared with that in *Prmt1* f/f mice throughout the HFD feeding period (Fig. 1F). As expected, accumulation of lipid droplets after 2-week HFD feeding was more prominent in livers of *Prmt1* LKO mice compared with those of the control (Fig. 1G). Unexpectedly, prolonged HFD feeding (4 to 8 weeks) resulted in the formation of more localized lipid droplets in livers of *Prmt1* LKO mice, while lipid droplets were spread uniformly throughout the liver in the case for *Prmt1* f/f mice. These data suggest that HFD feeding might enhance the progression of MASH in *Prmt1* LKO mice compared with that of *Prmt1* f/f mice. We observed an increased macrophage infiltration of livers of *Prmt1* LKO mice compared with those of the control, as shown by the F4/80 staining and the assessment of mRNA levels for proinflammatory cytokines (Fig. 1, H and I). To confirm the notion that hepatic depletion of *Prmt1* promotes MASH in mice, we fed mice with methionine/choline-deficient (MCD) diet for up to 8 weeks. While we observed more pronounced lipid droplets in the livers of control mice under both 4- and 8-week MCD diet without changes in body weight or liver weight, we noticed deformation of livers of *Prmt1* LKO mice compared with those of the control, hinting an increase in fibrosis (Fig. 1, J to L). We observed an enhanced Picrosirius Red staining and an increased expression of alpha smooth muscle actin (α-SMA) in the livers of *Prmt1* LKO mice compared with those of the control (Fig. 1, M and N), showing that hepatic depletion of *Prmt1* promotes diet-induced MASH in mouse models. Overall, we confirmed that *Prmt1* LKO mice reproduced diet-induced MASLD and MASH.

### NRF2 signaling is activated by *Keap1* down-regulation upon depletion of *Prmt1* in MASLD to MASH

**Fig. 2.**
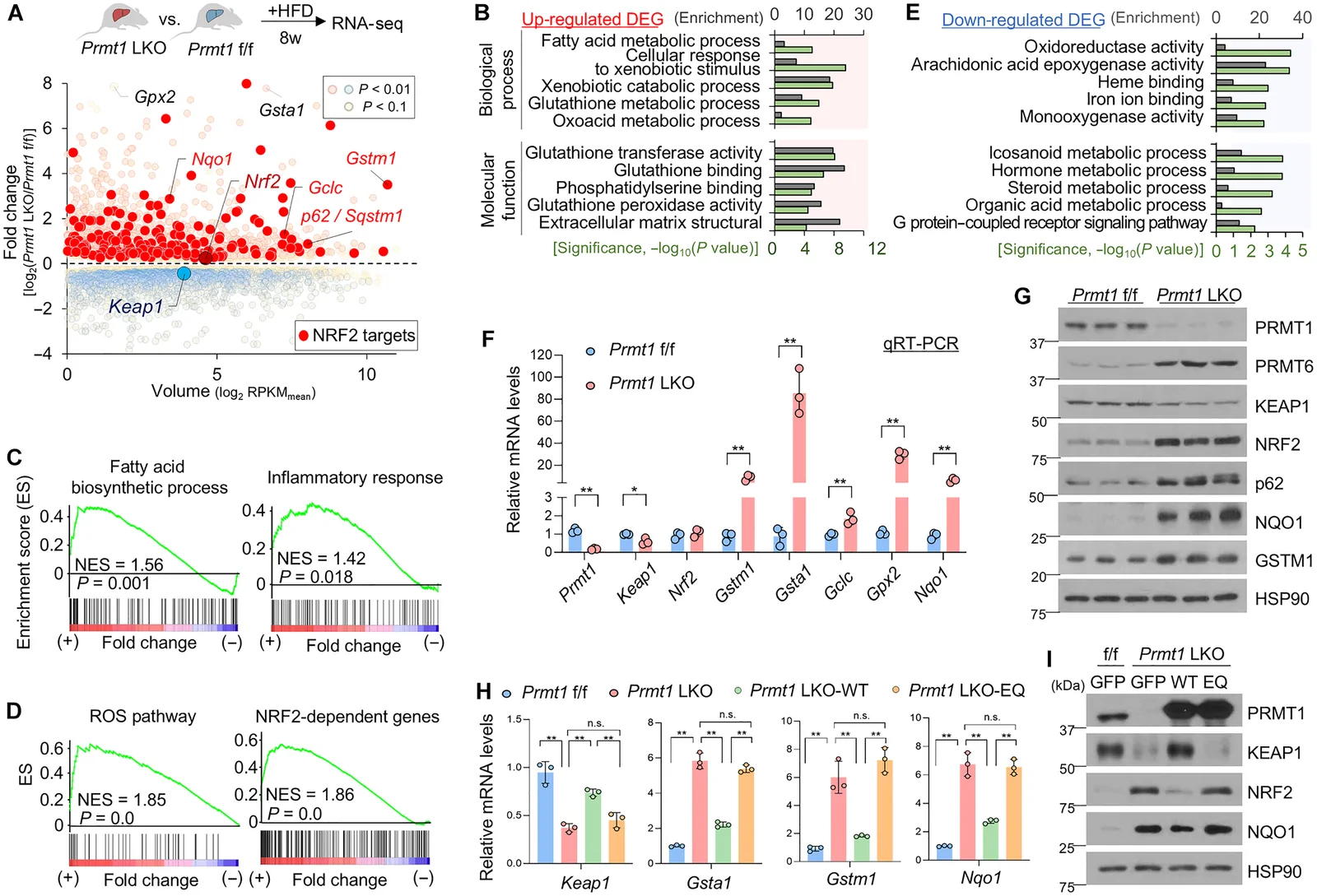
Depletion of *Prmt1* activates NRF2-dependent transcriptional program in the liver. (**A**) RNA-seq analysis in *Prmt1* LKO livers; MA plot relative to *Prmt1* f/f livers (*n* = 3 individual samples); *P* < 0.01 (Cuffdiff); faint red circle (up-regulation); blue circle (down-regulation); highlights for *Keap1*, *Nrf2*, and up-regulated NRF2 targets (red circle). 8w, 8 weeks. (**B**) Gene Ontology (GO) analysis for transcriptome profiles in *Prmt1* LKO liver; top five terms in molecular function and biological process, significantly enriched in up-regulated DEG (fold change > 2, *P* < 0.01); significance, EASE score from DAVID. (**C** and **D**) GSEA results with functional terms in MSigDB; fold change [log_2_ (*Prmt1* LKO/f/f)]. Normalized enrichment score (NES) and nominal *P* value. (**E**) Same GO analyses as in (B) except for down-regulated DEG (fold change < −2, *P* < 0.01). (**F**) The mRNA levels of NRF2 target genes were measured by qRT-PCR in the liver tissues from *Prmt1* f/f or *Prmt1* LKO mice under HFD for 8 weeks. Values represent means ± SEM (*n* = 3 per group). *P < 0.05 and **P < 0.01. (**G**) Western blot analyses showing expression of PRMTs, KEAP1, NRF2 and its downstream target proteins in the liver tissues from *Prmt1* f/f or *Prmt1* LKO mice under HFD for 8 weeks. (**H**) The mRNA levels of NRF2 target genes; qRT-PCR in the primary hepatocytes from *Prmt1* f/f or *Prmt1* LKO mice, infected with Ad-GFP, Ad-WT *Prmt1*, or Ad-mutant *Prmt1* [E153Q (EQ)]. Values represent means ± SEM (*n* = 3 per group). **P < 0.01. (**I**) Western blot analysis showing expression of PRMT1, KEAP1, NRF2, and its downstream target proteins in the primary hepatocytes from *Prmt1* f/f or *Prmt1* LKO mice, infected with Ad-GFP, Ad-WT *Prmt1*, or Ad-mutant *Prmt1* [E153Q (EQ)]. n.s., not significant.

To gain insight into the molecular mechanism by which *Prmt1* deficiency initiates diet-induced MASLD and promotes MASH phenotypes, we initially examined transcriptome profiles of livers from *Prmt1* LKO mice versus *Prmt1* f/f mice under HFD feeding by applying RNA-sequencing (RNA-seq) analysis (fig. S2A). Loss of *Prmt1* substantially changed gene expression profiles, with a number of differentially expressed genes (DEGs; *n* = 3190, *P* < 0.01; Fig. 2A and fig. S2B), implicating substantial reprogramming of transcriptome in *Prmt1* LKO liver. In concert with MASLD phenotype in *Prmt1* LKO mice, Gene Ontology (GO) analysis showed that up-regulated DEGs following depletion of *Prmt1* (fold change > 2, *P* < 0.01) play a significant role in lipid metabolic processes (Fig. 2B). In addition, gene set enrichment analysis (GSEA) revealed a significant enrichment in fatty acid biosynthesis, inflammatory response (Fig. 2C), and fibroblast proliferation pathways (fig. S2C).

The up-regulated DEGs were also enriched in xenobiotic detoxification and antioxidant processes (Fig. 2, A and B), wherein expression of genes involved in glutathione biosynthesis is known to be induced by the critical protumorigenic NRF2 transcription factor (*42*). GSEA in *Prmt1* LKO mice showed activation of ROS pathway and NRF2 target genes, which include NRF2-dependent genes (Fig. 2D) and direct NRF2 targets (fig. S2D) (*43*). Concordantly, we observed down-regulation of the redox sensing *Keap1* mRNA (Fig. 2A and fig. S2B) and significant suppression of genes functioning in metabolic pathways requiring oxygen-dependent reactions according to GO analysis in down-regulated DEGs (fold change < −2, *P* < 0.01; *n* = 74; Fig. 2E). Such changes in the transcriptome implicate a metabolic shift toward excess lipid biosynthesis as well as antioxidant responses via activation of the protumorigenic NRF2 pathway. We were able to confirm that NRF2 target genes (*Gstm1*, *Gsta1*, *Gclc*, *Gpx2*, and *Nqo1*) were highly up-regulated in *Prmt1* LKO mice using quantitative reverse transcription polymerase chain reaction (qRT-PCR) analysis (Fig. 2F).

Next, we examined the levels of proteins related to the NRF2 pathway. As expected, we observed a marked increase in NRF2 and its targets, NQO1 and GSTM1, and a decrease in KEAP1 protein levels in livers from *Prmt1* LKO mice (Fig. 2G). We found that expression of PRMT6, another type I PRMT, was elevated in *Prmt1* LKO mice compared with that in the control (Fig. 2G). Based on our earlier findings in skeletal muscle and white adipose tissue (*20*, *21*), such compensatory PRMT6 paralog expression could be explained by the removal of a transcription corepressor complex, recruited by PRMT1 on the promoter of *Prmt6* (fig. S3A).

Furthermore, we performed a couple of rescue experiments in primary hepatocytes from either *Prmt1* f/f mice or *Prmt1* LKO mice. Ectopic expression of enzymatically active PRMT1, but not functionally defective PRMT1 (EQ), in *Prmt1* LKO hepatocytes restored KEAP1 expression, resulting in down-regulation of NRF2 protein levels and its target gene expression (Fig. 2, H and I). Similarly, overexpression of KEAP1 in *Prmt1*-depleted hepatocytes also reduced NRF2 protein levels and its target gene expression (fig. S3, B and C). These data collectively demonstrate that depletion of *Prmt1* directly reduced expression of KEAP1, resulting in the constitutive activation of NRF2 in the liver.

Notably, we also observed a marked increase in p62 protein (Fig. 2G), accumulation of p62 aggregates (fig. S4A), and ROS (fig. S4B) in livers of *Prmt1* LKO mice. Increased expression of p62 by NRF2 could promote accumulation of p62 in the liver as a form of Mallory-Denk bodies, which is recognized as a marker for MASLD including MASH and HCC in the clinic (*44*–*46*), suggesting that depletion of *Prmt1* could promote hepatic fibrosis via the induction of p62 accumulation. In line with this, we tested whether restoration of PRMT1 expression could deter the progression of MASH in *Prmt1* LKO mice. We injected an adenovirus expressing *Prmt1* into *Prmt1* LKO mice under the MCD diet and observed that 2-week expression of PRMT1 in *Prmt1*-deficient mice reduced ROS accumulation, macrophage infiltration, and fibrosis in the liver (fig. S4, C to E).

These changes were accompanied by restoration of KEAP1 expression and the resultant reduction in NRF2 and p62 expression, further supporting the hypothesis that the increased progression of MASH in *Prmt1*-deficient mice was due to the hyperactivation of the NRF2-p62 axis (fig. S4, F and G). Compensatory elevation of PRMT6 in *Prmt1* LKO mice could be reversed by expressing *Prmt1* (fig. S4F), implicating its potential role in regulating these pathways differently from PRMT1. Overall, all of these results suggest that hepatic *Prmt1* depletion down-regulates KEAP1, thereby activating NRF2 pathways for the progress of MASLD to MASH while simultaneously up-regulating PRMT6.

### Compensatory PRMT6 expression controls m^6^A-modifying machineries in *Prmt1* LKO mice

Next, we wanted to explore the potential mechanism by which depletion of *Prmt1* reduced KEAP1 expression in the liver. Recent data revealed that hepatic depletion of *Prmt1* led to the reduction in HNF4α expression and its downstream target genes upon acute and chronic alcohol consumption in mice (*25*), prompting us to look for the potential involvement of this factor in the regulation of KEAP1 expression. We failed to observe any effect of ectopic HNF4α expression on KEAP1 protein levels in hepatocytes, excluding the potential involvement of this factor in this pathway (fig. S5A). Furthermore, while we saw no changes in protein levels of HNF4α upon *Prmt1* knockout, we observed a small increase in *Hnf4a* mRNA levels in *Prmt1*-depleted livers of HFD-fed mice compared with those in the control (fig. S5, B and C). As a result, the potential target genes of HNF4α were rather induced upon liver-specific depletion of *Prmt1*. These data suggest that HNF4α was not involved in the development of MASH as opposed to alcoholic steatohepatitis (ASH) in *Prmt1* LKO mice.

**Fig. 3.**
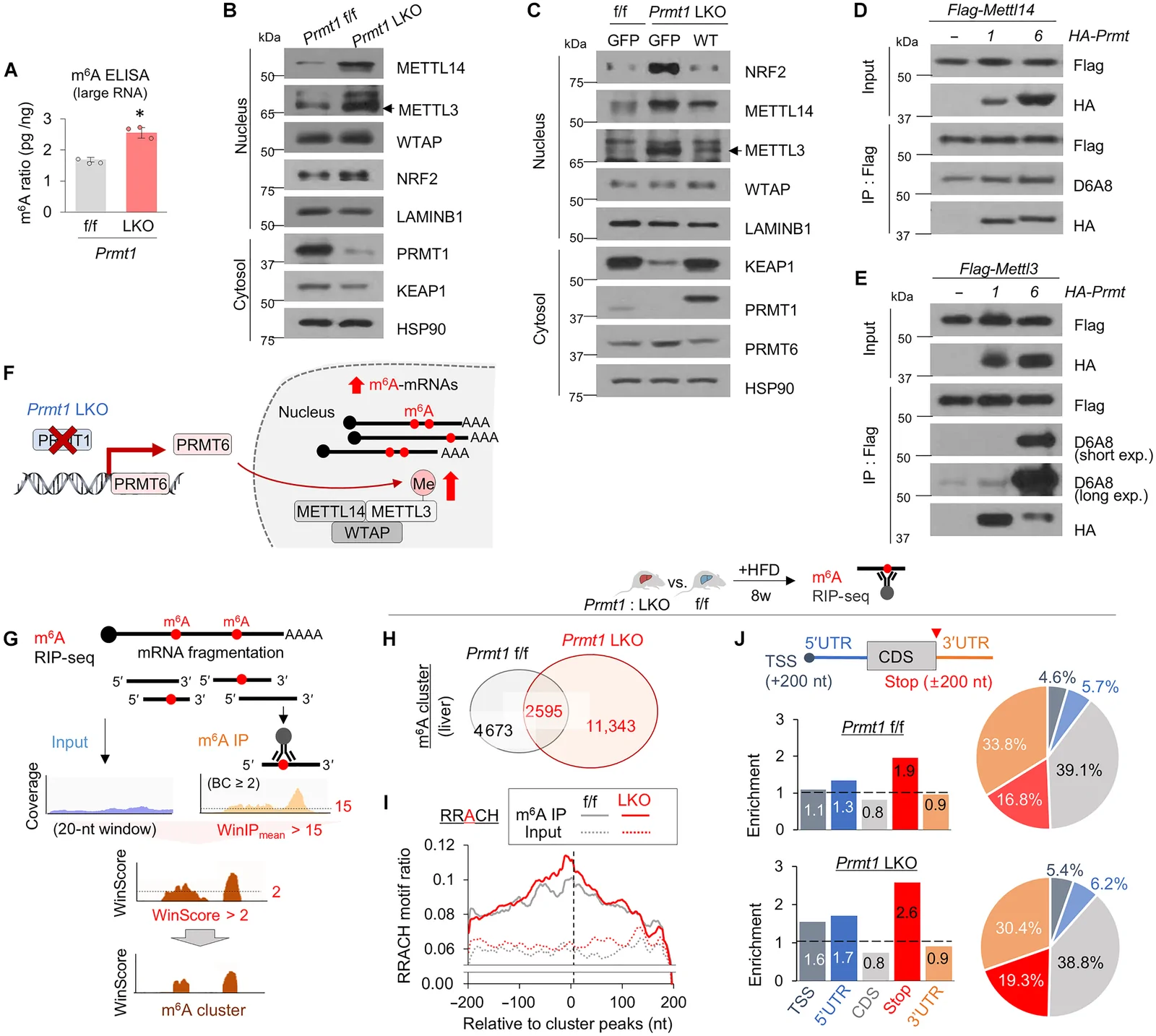
Hepatic *Prmt1* deficiency induces *Prmt6* that activates the m^6^A-modifying machinery in the nucleus. (**A**) Quantification of m^6^A in large RNA [>200 nucleotides (nt); extracted from liver; enzyme-linked immunosorbent assay (ELISA)]; *Prmt1* LKO and *Prmt1* f/f mice under 8-week HFD; *P = 0.001 (*n* = 3); m^6^A ratio, amount of m^6^A (picograms) per total RNA (nanograms). (**B**) Western blot analysis for the m^6^A-modifying machinery and NRF2 in the nucleus of liver tissues; *Prmt1* f/f versus *Prmt1* LKO mice under normal chow diet; *n* = 3 independent experiments mice per group. (**C**) Same Western blot analysis as in (B) except infection with Ad-GFP or Ad-WT *Prmt1*; *n* = 3 independent experiments mice per group. (**D** and **E**) Coimmunoprecipitation between METTL14 (D) or METTL3 (E) and PRMTs. Asymmetric dimethylarginine (ADMA; D6A8) levels of METTL proteins were also shown; *n* = 3 independent experiments mice per group. (**F**) Schematic diagram of the compensatory up-regulation of PRMT6 following hepatic *Prmt1* depletion, resulting in asymmetric arginine dimethylation of METTL3 and activation of writer complex for m^6^A mRNA modifications. (**G**) m^6^A RIP-seq analysis; WinIP_mean_, mean coverage of m^6^A IP (20-nt window). m^6^A clusters were selected using WinScore (log ratio of normalized coverages between m^6^A IP and input control; >2) and WinIP_mean_ (>15); 4.6% of false discovery rate (FDR; ratio of selected windows in m^6^A IP relative to the cases with input being treated as IP; <5%). (**H**) Venn diagram of the m^6^A clusters; *Prmt1* LKO versus *Prmt1* f/f mice. (**I**) Enrichment of RRACH motif (ratio) relative to peak position of unique m^6^A mRNA clusters. Unique clusters derived from input were used as a control. (**J**) Positional enrichment of m^6^A mRNA clusters for five annotated segments (top); enrichment, relative ratio of observed frequency to the expected frequency considering total nucleotides of the given annotation (left); fraction to total number of annotations (right).

Recent studies revealed that PRMT1 is responsible for the activation of METTL14 in an arginine methylation–dependent manner during differentiation of mouse embryonic stem cells (*38*). Because protumorigenic cell states for MASLD-associated HCC precede oncogenic somatic mutations, alteration of transcriptome might be initially induced in *Prmt1* LKO mice by changing global RNA regulation. In line with this, *Keap1* expression could be regulated at the level of mRNA stability, perhaps due to its modification via m^6^A–dependent manner. We checked the level of m^6^A in large RNA [>200 nucleotides (nt)] by enzyme-linked immunosorbent assay (ELISA) and found significant increase of m^6^A modification in *Prmt1* LKO mice compared with that in *Prmt1* f/f mice (Fig. 3A). After observing changes in m^6^A level upon depletion of *Prmt1*, we then wanted to check whether arginine methylation may affect the m^6^A-modifying machineries.

Because METTL14 forms a complex with METTL3 and is predominantly localized in the nucleus in association with WTAP to function as a methyltransferase complex for m^6^A of mRNAs (*28*), we analyzed protein levels of these proteins in the nucleus of livers from either *Prmt1* f/f mice or *Prmt1* LKO mice. Unexpectedly, unlike our expectation, we found that protein levels of METTL14, as well as METTL3, were highly elevated in the hepatic nuclear fraction of *Prmt1* LKO mice compared with that of *Prmt1* f/f mice (Fig. 3B). These trends were abolished by an ectopic expression of PRMT1 in *Prmt1* KO hepatocytes (Fig. 3C). Additionally, as noted above (Fig. 2G), PRMT6 protein levels were markedly increased in *Prmt1* LKO hepatocytes, suggesting a potential role of this methyltransferase in the absence of PRMT1 (Fig. 3C). As METTL14 was shown to be activated by PRMT1-mediated asymmetric arginine dimethylation, we tested whether PRMT6 could also function as an arginine methyltransferase for METTL14 in the absence of PRMT1. In our hands, both PRMT6 and PRMT1 were only marginally functional in conferring asymmetric dimethylation on arginine residues of METTL14 (Fig. 3D). Unlike METTL14, asymmetric arginine dimethylation of METTL3 by PRMTs has not been reported to date. Unexpectedly, we found that PRMT6, but not PRMT1, catalyzed asymmetric arginine dimethylation of METTL3 (Fig. 3E). Overall, these data strongly suggest that paralog compensation by PRMT6 in the absence of PRMT1 may result in elevated m^6^A mRNA modifications by enhancing asymmetric arginine dimethylation of METTL3 (Fig. 3F).

### Elevated m^6^A mRNA modifications in *Prmt1*-deficient liver

To test whether the elevated level of nuclear METTL3/14 complex in the absence of PRMT1 (and with a compensatory increase in PRMT6) could affect m^6^A modification (Fig. 3F), we attempted to map m^6^A in mRNAs by applying m^6^A RNA immunoprecipitation sequencing (*47*, *48*) and analysis (*47*) (m^6^A RIP-seq) (Fig. 3G). Based on reproducible reads [biological complexity (BC) ≥ 2; fig. S6A], m^6^A clusters were defined (Fig. 3G) by selecting enriched read counts in m^6^A immunoprecipitation [m^6^A IP; false discovery rate (FDR) < 5%; Fig. 3G and fig. S6, B and C] more than thresholds within the transcript (WinIP_mean_ > 15), relative to the input control (WinScore > 2). As m^6^A IP signals, which exceeded the selected thresholds, were found to be increased in *Prmt1* LKO in comparison with *Prmt1* f/f mice (fig. S6D), the m^6^A clusters were analyzed to be 1.9-fold more abundant in *Prmt1* LKO mice (*n* = 13,938) than in *Prmt1* f/f mice (*n* = 7268) with some overlaps (*n* = 2595; Fig. 3H). We also confirmed that m^6^A methylation motif (RRACH) was also observed more frequently near peaks of the m^6^A clusters in *Prmt1* LKO mice than those in in *Prmt1* f/f mice by analyzing unique m^6^A mRNA clusters (relative to input, Fig. 3I; relative to control motifs, fig. S6E). As previously reported (*48*), m^6^A mRNA clusters in *Prmt1* LKO mice were mostly derived from the 3′UTR including stop codon (49.7%) and more enriched in the vicinity of the stop codon than the clusters in *Prmt1* f/f mice (enrichment, 2.6 versus 1.9; relative to expected frequency; Fig. 3J). Albeit to a lesser extent, m^6^A mRNA clusters in *Prmt1* LKO mice were also more frequently observed in 5′UTR (1.7 versus 1.3) and transcription start site (TSS; 1.6 versus 1.1) than the clusters in *Prmt1* f/f mice (Fig. 3J)

### m^6^A modifications activate protumorigenic NRF2 pathway by inducing mRNA degradation in *Prmt1*-deficient liver

**Fig. 4.**
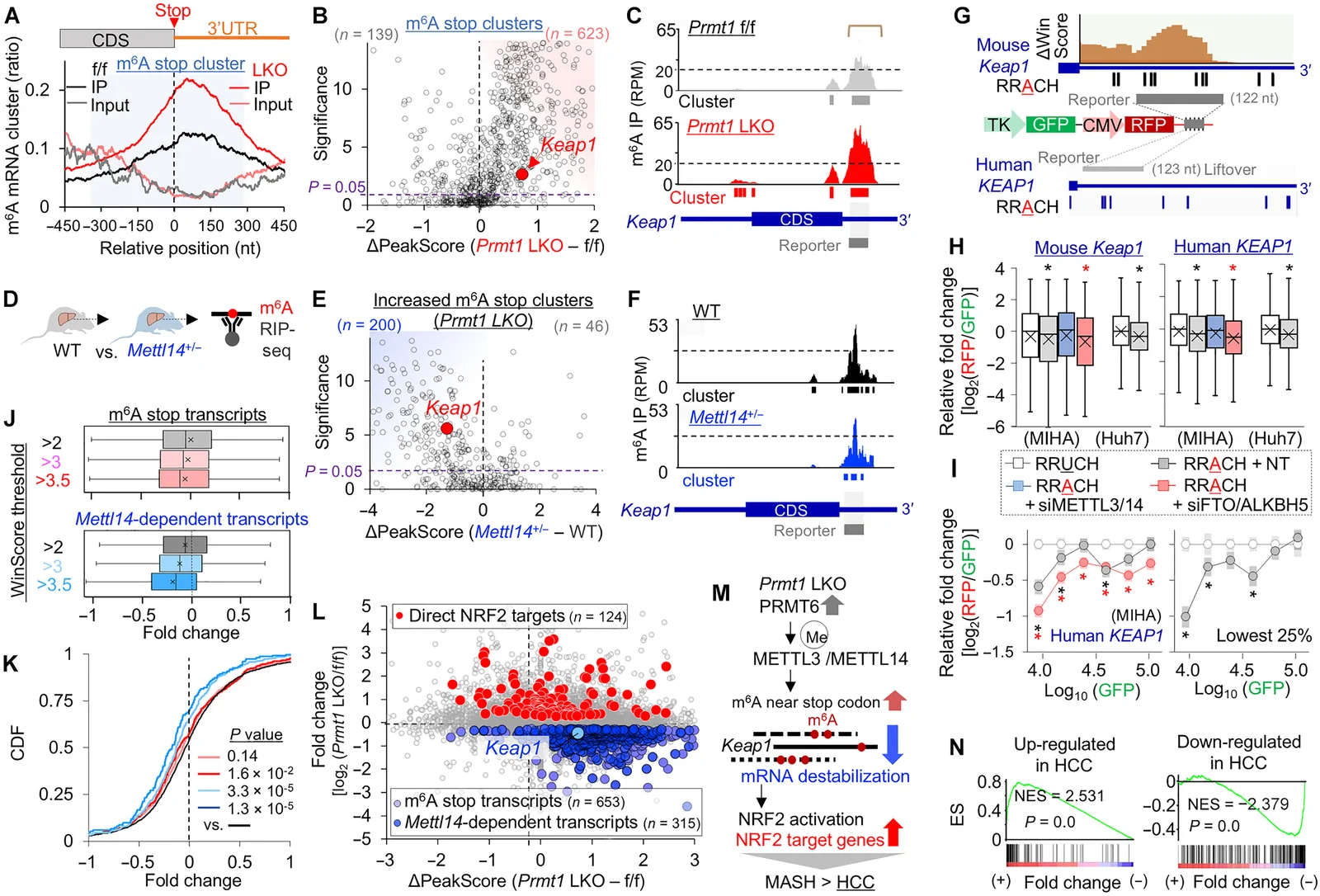
Down-regulation of m^6^A-modified mRNAs activates the KEAP1-NRF2 pathway in *Prmt1*-deficient liver. (**A**) m^6^A clusters relative to stop codon; *Prmt1* f/f versus LKO mice; m^6^A stop cluster (±300 nt). Input-derived clusters were used as controls. (**B**) Volcano plot for differentially induced m^6^A stop clusters in *Prmt1* LKO; ΔPeakScore and statistical significance [−log_10_ (*P* value); *t* test for WinScore, unpaired two-tailed]. (**C**) m^6^A IP reads in *Keap1* mRNA; “reporter,” the region used for reporter assays. (**D** to **F**) m^6^A RIP-seq in hepatocytes of *Mettl14^+/−^* mice (D). ΔPeakScore analysis as in (B) except using Mettl14^+/−^ for increased m^6^A stop clusters in *Prmt1* LKO (ΔPeakScore > 0, *P <* 0.05; E). m^6^A IP reads in *Keap1* mRNA (F). (**G**) The m^6^A-enriched region in mouse *Keap1* mRNA [ΔWinScore (*Prmt1* LKO − f/f)] and the corresponding region in human (liftover), used in dFP reporters. (**H**) Cytometry of dFP reporters (*n* ≥ 1784 cells); relative fold change, normalized to the median value from control dFP reporters (RRUCH); knockdown of m^6^A writers (siMETTL3/14) or erasers (siFTO/ALKBH5); control siRNA (NT). (**I**) Relative fold change depending on the level of dFP vector expression [log_10_(GFP); left], further analyzed for only the lowest 25% subset of RFP signals (right). Values represent means ± SEM; *P < 0.05, relative to RRUCH. (**J** to **L**) Distribution of fold change [log_2_ (*Prmt1* LKO/*Prmt1* f/f)] in “m^6^A stop transcripts” (±300 nt of stop codon) and their subset, METTL14-dependent transcripts [ΔPeakScore (*Mettl14*^+/−^ − WT) < 0], depending on WinScore threshold (box plot, J; CDF, K; *P* value, two-sided KS test) or m^6^A enrichment in *Prmt1* LKO (ΔPeakScore), highlighting down-regulation (including *Keap1*) and up-regulation of direct NRF2 targets (*43*). (**M**) Hepatic *Prmt1* depletion leads to MASH and HCC progression. (**N**) GSEA results; up-regulated or down-regulated genes in HCC (*Txnip*^−/−^); NES and nominal *P* value.

In *Prmt1* LKO mice, we observed that m^6^A mRNA clusters were predominantly enriched near the stop codon (±300 nt, relative to control clusters derived from input; Fig. 4A). Such m^6^A stop clusters in *Prmt1* LKO mice (*n* = 4672) were 2.4-fold more abundant than those in *Prmt1* f/f mice (*n* = 1922), representing significant propensity of elevation in peak heights (ΔPeakScore) for the overlapping clusters (*n* = 989; Fig. 4B and fig. S7A), and represented by enriched m^6^A deposition on *Keap1* mRNA (Fig. 4C). The m^6^A stop clusters seemed to be directly mediated by the METTL3/14 complex because m^6^A RIP-seq in livers of *Mettl14*^+/−^ mice (Fig. 4D and fig. S7B) (*49*) showed significant reduction of the increased m^6^A stop clusters (ΔPeakScore < 0, *P* < 0.05; Fig. 4E) that were identified in *Prmt1* LKO mice (*n* = 623; ΔPeakScore > 0, *P* < 0.05; Fig. 4B); therefore, we named these “METTL14-dependent m^6^A stop clusters.” The m^6^A stop clusters in *Keap1* mRNA, which was markedly induced in *Prmt1* LKO mice, were identified to be significantly reduced in *Mettl14*^+/−^ mice (Fig. 4F). *Mettl14*^+/−^ mice also exhibited marked decrease of peak heights in IP clusters (PeakScore; fig. S7C) as well as in m^6^A clusters of *Prmt1* LKO mice (WinScore; fig. S7, D to F).

Next, regulatory activity of m^6^A-modified sequence in mouse *Keap1* mRNA, as well as the conserved sequence in human *KEAP1* mRNA, was functionally investigated in human liver cell lines (Huh7 and MIHA) using luciferase reporter assays (fig. S8, B and C) after validating small interfering RNAs (siRNAs) (fig. S8A) against m^6^A erasers (siFTO/ALKBH5) and writers (siMETTL3/14). Luciferase reporters with m^6^A methylation sites (RRACH) were repressed relative to the reporters with mutant sites (RRUCH; fig. S8, B and C), more significantly after siFTO/ALKBH5 transfection (fig. S8B). Although marginal, siMETTL3/14 transfection showed derepression of the reporters with m^6^A methylation sites in MIHA, but not in Huh7 (fig. S8, B and C), presumably confounded by hyperactivation of m^6^A methyltransferase (*50*, *51*), and cellular heterogeneity observed in liver cancer cell. To examine m^6^A-mediated reporter activity at the single-cell level, we conducted flow cytometric analyses using dual fluorescent protein (dFP) reporters (Fig. 4, G to I), which express a red fluorescent protein (RFP) gene that includes the m^6^A-modified sequence (RRACH) near the stop codon and a green fluorescent protein (GFP) gene that has no site (relative fold change; Fig. 4G). The same m^6^A-dependent repression of the m^6^A methylation sites (RRACH) was significantly observed in the distributions of relative fold change (Fig. 4H). Notably, when only the cells with the lowest 25% RFP values in dFP reporter for human *KEAP1* were considered in MIHA, relative fold change of m^6^A methylation site was quantified as effectively as the case of siFTO/ALKBH5 transfection (Fig. 4I), indicating that a distinct subpopulation that harbors high m^6^A-mediated *Keap1* mRNA repression in hepatocytes. In support of our model, we observed that mRNA stability of *Keap1* was significantly lower in *Prmt1* LKO hepatocytes compared with that in the control hepatocytes (fig. S8D), and treatment of METTL3 inhibitor (STM2457) significantly stabilized *Keap1* mRNA levels in *Prmt1* LKO hepatocytes (fig. S8E).

As observed in global transcript levels (volcano plot; fig. S8F), the m^6^A stop transcripts showed significant repressive propensity depending on the degree of elevation in peak height (WinScore thresholds in *Prmt1* LKO), especially evident in METTL14-dependent transcripts [ΔPeakScore (*Mettl14*^+/−^ − WT) < 0] as analyzed in box plot (Fig. 4J) and cumulative distribution function (CDF; Fig. 4K). These analyses could explain alterations of transcriptome in *Prmt1* LKO mice for both down-regulation and up-regulation, in that METTL14-mediated elevation of m^6^A modification near stop codon destabilized the corresponding m^6^A target transcripts including *Keap1* mRNA, thereby transcriptionally activating direct NRF2 targets (Fig. 4L).

To address the role of m^6^A regulation on KEAP1-NRF2 axis, we performed both gain-of-function and loss-of-function experiments for m^6^A writer machinery in primary hepatocytes. Overexpression of METTL3 and METTL14 in primary hepatocytes reduced expression of KEAP1, leading to the elevated expression of NRF2 and its downstream target NQO1 (fig. S9A). Conversely, inhibition of m^6^A writer machinery by treatment of METTL3 inhibitor (STM2457) partially restored KEAP1 expression in *Prmt1* LKO hepatocytes, resulting in the repression of NRF2 expression and its downstream target NQO1 (fig. S9, B to D). These data underscored the role of m^6^A machinery for controlling KEAP1-NRF2 axis in the liver.

Overall, compensatory expression of PRMT6, caused by hepatic *Prmt1* depletion, induces hyperactivation of the m^6^A methyltransferase complex (METTL3 and METTL14) that results in enriched m^6^A deposition near stop codon of mRNAs including *Keap1* for destabilization, thereby leading to chronic activation of NRF2 targets that potentially drive MASH and the progression to HCC (Fig. 4M). In support of protumorigenic HCC progression, up-regulated genes in *Prmt1* LKO mice significantly correlated with genes functioning in epithelial-mesenchymal transition (EMT) pathways (GSEA; fig. S10A), as reported in NRF2-induced carcinogenesis (*52*, *53*). Furthermore, transcriptome profiles of *Prmt1* LKO mice significantly correlated with both up-regulated [normalized enrichment score (NES) = 2.531, *P* = 0.0] and down-regulated genes (NES = −2.379, *P* = 0.0) in oxidative stress-induced HCC mouse model (GSEA; Fig. 4N) genetically induced by dysregulation of redox balance (*Txnip*^−/−^) (*54*). Therefore, m^6^A-mediated down-regulation in *Prmt1* LKO mice also appears to increase susceptibility for hepatocarcinogenesis.

### Compensatory induction of PRMT6 with reduced PRMT1 expression is associated with premalignancy in patients with HCC

**Fig. 5.**
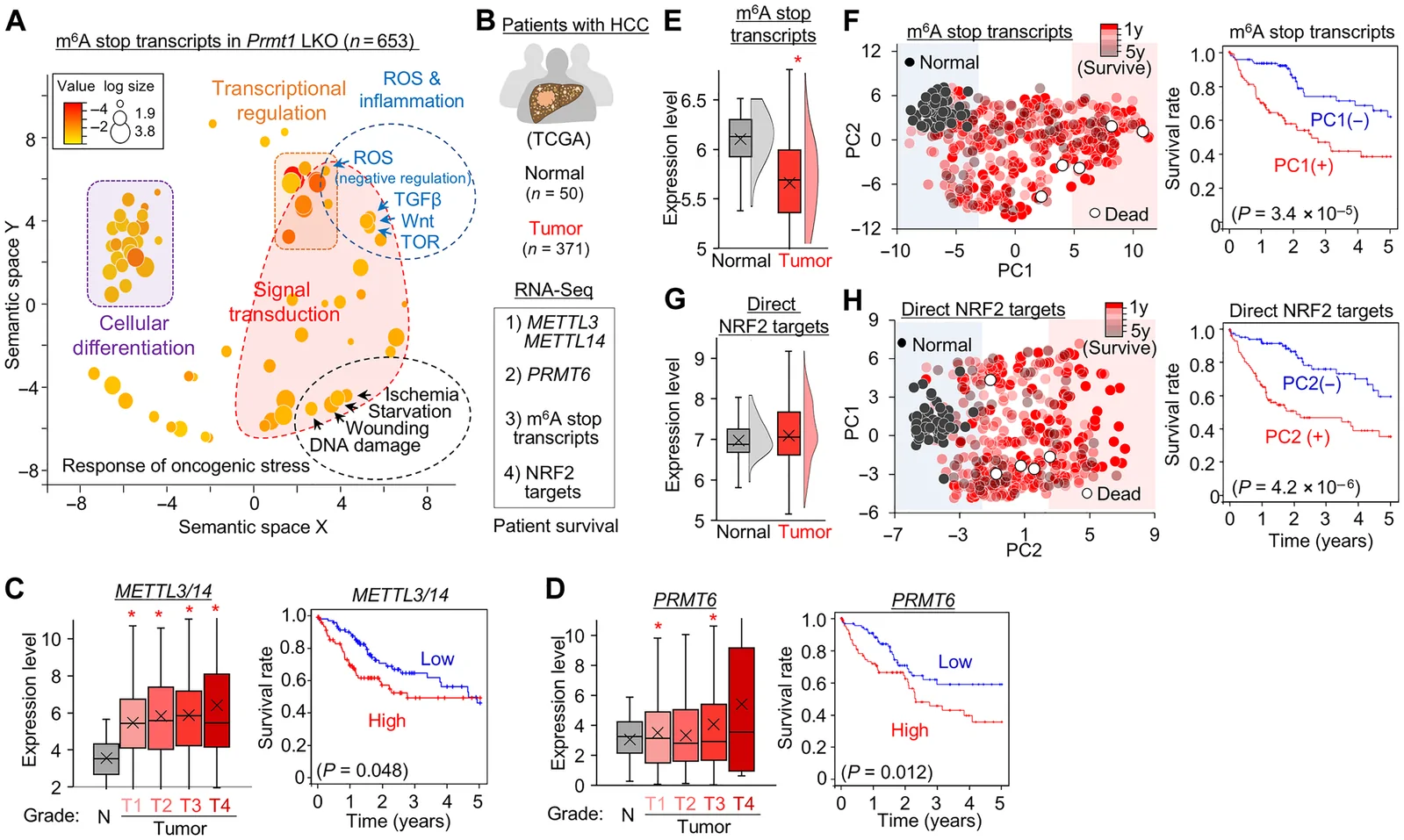
*PRMT1* down-regulation, compensatory *PRMT6* induction, and NRF2 activation associate with patients with MASLD-associated HCC. (**A**) GO analysis of m^6^A stop transcripts identified in *Prmt1* LKO mice (*n* = 653); semantic space as cluster representatives; the color and proportional size of circles indicate log_10_(*P* value); *P* values were obtained using DAVID. TGFβ, transforming growth factor–β. (**B**) Cohort analysis of patients with HCC (TCGA-LIHC; normal, *n* = 50; tumor, *n* = 371), comparing gene expression levels (RNA-seq) and patient survival for PRMT6-METTL-m6A-NRF2 pathways. (**C**) Average expression levels of *METTL3* and *METTL14* in patients with HCC across different tumor grades [normal tissue (N); T1, T2, T3, and T4 grades], represented as box plots (left). Kaplan-Meier survival analysis with patients with HCC (right); high expression (top quartile, 25%) versus low expression (bottom quartile, 25%) of *METTL3* and *METTL14*; 5-year survival rate, *P = 0.048. (**D**) Same analyses as in (C) except for *PRMT6*. *P = 0.012. (**E**) Average expression levels of the m^6^A stop transcripts in normal and tumor tissues of patients with HCC, displayed with box plot and violin plot. (**F**) PCA analysis results with highly variable genes in the m^6^A stop transcripts, colored with patient survival years (left); Kaplan-Meier survival analysis as in (C) except using PC1 values (right); *P = 3.4 × 10^−5^. 1y, 1 year; 5y, 5 years. (**G** and **H**) Same analyses as in (E) and (F) except using direct NRF2 targets and PC2; *P* = 4.2 × 10^−6^.

In accordance with changes of transcriptome featured with tumor-promoting function, m^6^A stop transcripts in *Prmt1* LKO mice were significantly enriched in GO terms related to ROS and inflammation (transforming growth factor–β, Wnt, and mTOR pathways) and response to oncogenic stress (ischemia, starvation, wound, and DNA damage response) as part of cellular differentiation, transcriptional regulation, and signal transduction functions (Fig. 5A), implicating a substantial contribution of m^6^A mRNA modifications to these protumorigenic changes. Similar GO results were also observed for METTL14-dependent transcripts (fig. S10B).

Further expanding the analyses to patients with HCC [The Cancer Genome Atlas (TCGA); Fig. 5B], we observed elevated levels of m^6^A methyltransferases (*METTL3* and *METTL14*) that correlated with exacerbated tumor grades and survival rates (Fig. 5C). Similar results were also obtained for *PRMT6* as an activator of the METTL3/14 complex (Fig. 5D). Of note, m^6^A stop transcripts tended to be down-regulated during HCC progression, associated with tumor malignancy (Fig. 5E and fig. S10C). Highly variable genes in m^6^A stop transcripts showed distinct discrimination between normal and tumor tissues in principal components analysis (PCA), significantly correlating with patient survival (Fig. 5F). The same contribution from up-regulating direct NRF2 targets to tumor progression (Fig. 5G and fig. S10D) was also confirmed by the PCA and survival analysis in patients with liver HCC (LIHC; Fig. 5H).

**Fig. 6.**
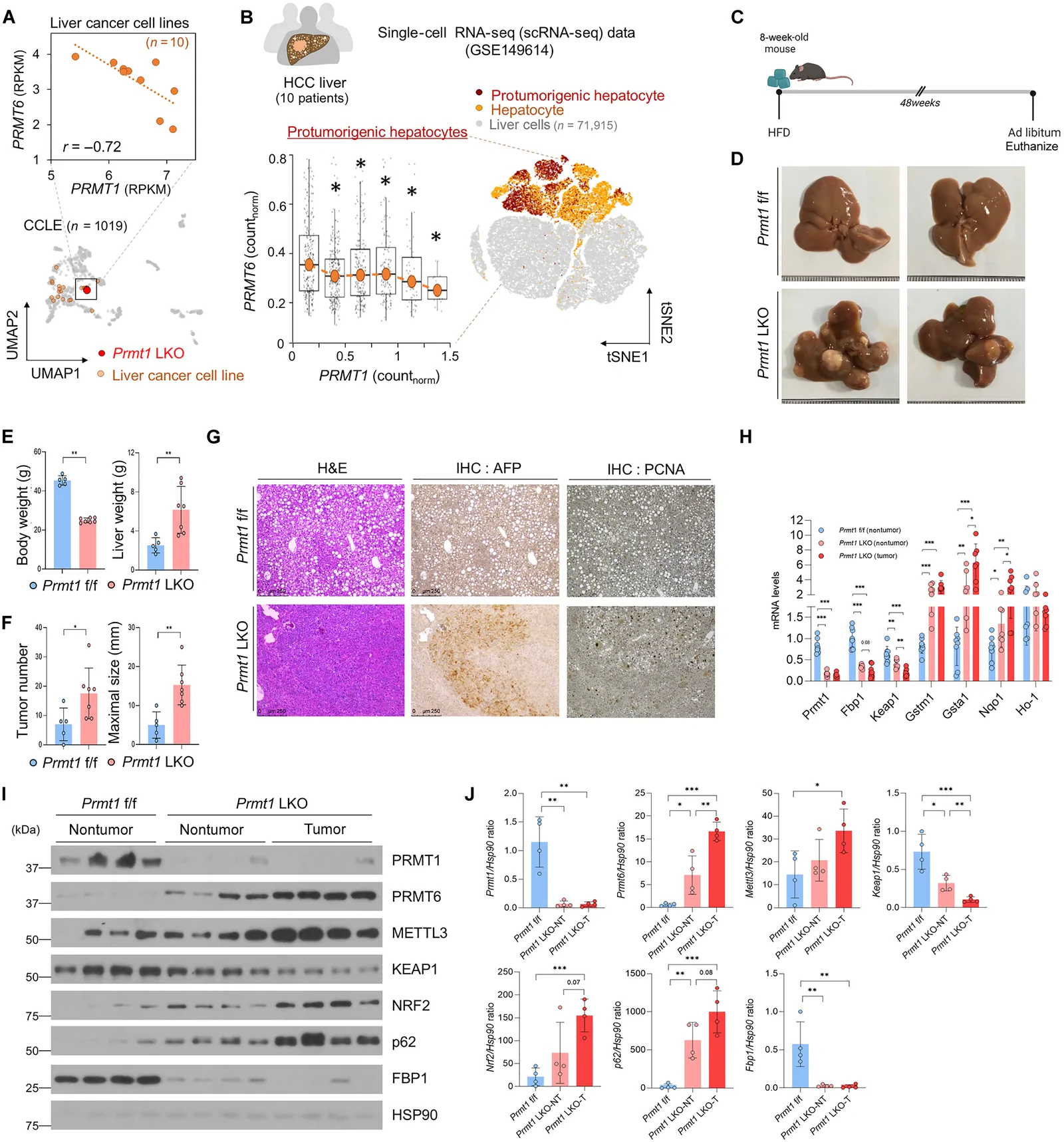
Hepatic depletion of *Prmt1* promotes MASLD-associated HCC in mice. (**A** and **B**) Comparison between *PRMT1* and *PRMT6* expression in liver cancer cell lines [RPKM; correlation coefficient (*r*)], selected as clustered with *Prmt1* LKO (UMAP; gene expression profiles) in Cancer Cell Line Encyclopedia (CCLE; A), or in protumorigenic hepatocyte population (count_norm_, normalized read count), identified by scRNA-seq in patients with HCC (*n* = 10, GSE149614; B); *P < 0.05 (two-sided *t* tests), relative to cells with count_norm_ < 0.25. (**C** to **J**) Schematic diagram for the generation of MASLD-associated HCC (HFD for 48 weeks; C) in *Prmt1* f/f or *Prmt1* LKO mice and representative picture of their livers (D). Body weight and liver weight (*n* = 8 for *Prmt1* f/f mice and *n* = 7 for *Prmt1* LKO mice; E). Tumor nodule number and size (*n* = 7 mice per group; F). H&E staining, Alpha-fetoprotein (AFP) staining and proliferating cell nuclear antigen (PCNA) staining (*n* = 3 mice per group; G). Scale bars, 250 μm (×100). The mRNA levels of NRF2 target genes (qRT-PCR; *n* = 7 for *Prmt1* f/f mice and *n* = 6 to 7 for *Prmt1* LKO mice; H). Western blot analysis showing expression of PRMTs, KEAP1, METTL3, FBP1, p53, NRF2, and its downstream target proteins (*n* = 4 per group; I). Quantitation of each band shown in (I) (*n* = 4 per group; J). Values represent means ± SEM. *P < 0.05, **P < 0.01, and ***P < 0.001. (C) Created in BioRender. S. Kim (2026), https://BioRender.com/o2vy791.

Particularly, compensatory induction of *PRMT6* was observed with decreasing *PRMT1* expression in HCC cell lines [correlation coefficient (*r*) = −0.72], classified as the same expression types as *Prmt1* LKO mice in Cancer Cell Line Encyclopedia (CCLE; Fig. 6A). In contrast, *PRMT1* expression showed a marginal correlation with *PRMT6* in most cancer cell lines (fig. S10E). In the gene expression analyses of single cells in patients with HCC [single-cell RNA sequencing (scRNA-seq); *n* = 10] (*55*) protumorigenic hepatocytes particularly exhibited a negative correlation between *PRMT1* and *PRMT6* expression (Fig. 6B) more evidently than the total hepatocyte population (fig. S10F). Overall, as expression of m^6^A methyltransferases and subsequent oncogenic destabilization of m^6^A stop transcripts and induction of NRF2 targets are clinically related to malignancy of patients with HCC, we observed compensatory induction of *PRMT6* with decreasing *PRMT1* expression occurring in protumorigenic hepatocytes in liver cancer.

### Chronic activation of premalignant m^6^A-KEAP1-NRF2 pathway promotes MASLD-associated HCC in patients and *Prmt1* LKO mice

Because we observed that *Prmt1* deficiency and the resultant activation of PRMT6 could be associated with premalignancy in NRF2-dependent manner, we decided to investigate whether the depletion of *Prmt1* in the liver promotes MASLD-associated HCC in mouse models. To test the effect of Prmt1 deficiency on more natural MASLD-associated HCC, we fed mice under 1-year HFD feeding (Fig. 6C). We found that prolonged HFD feeding induced a marked increase in liver weight despite the overall reduction in body weight, and it accelerated formation of tumors in *Prmt1* LKO mice (Fig. 6, D and E). Both the overall number and the maximal size of tumors were increased more than twofold in livers of *Prmt1* LKO mice compared with those of the control (Fig. 6F). Alpha-fetoprotein and proliferating cell nuclear antigen staining showed that there was an increased incidence of HCCs in livers of *Prmt1* LKO mice (Fig. 6, G and H). We found a similar result with chemically induced HCC as the number and the size of tumors were higher in *Prmt1* LKO mice (fig. S11, A to E). As expected, we also observed a persistent activation of the NRF2 pathway, as evidenced by the increased NRF2 protein levels and the decreased KEAP1 protein levels, as well as increased expression of NRF2 targets and p62 in livers of DEN-injected *Prmt1* LKO mice (Fig. 6, H to J; and fig. S12, A to C).

Recently, it was shown that activation of NRF2 and AKT induced the decline in FBP1 and p53 expression in a protumorigenic daHep subpopulation, leading to the inhibition of cellular senescence and the resultant premalignant state to HCC (*10*). As expected from the increased incidence of HCC in *Prmt1* LKO mice (Fig. 6, C to G), we observed a reduced expression of FBP1 and that stemmed from an induction of the PRMT6-METTL3-NRF2 axis in *Prmt1*-deficient livers (Fig. 6, H to J; and fig. S12, A to C). We found that PRMT6-METTL3-p62-NRF2 were more specifically up-regulated and that KEAP1 expression was more specifically down-regulated in tumors compared with adjacent nontumors in *Prmt1* LKO livers, although the aforementioned axis was significantly elevated even in nontumors of *Prmt1* LKO livers compared with those of WT livers. These data suggest that PRMT6-METTL3-p62-KEAP1-NRF2 axis could be important in driving severe liver disease and is further accentuated during the progression of steatotic HCC. Focusing on the daHeps, we found that *Prmt1* LKO livers showed significant enrichment of daHep marker genes (*12*) (GSEA; NES = 1.40, *P* = 0.007), suggesting an expansion of the daHeps in *Prmt1* LKO liver (fig. S13A). Using scRNA-seq results in patients with MASLD-associated HCC, we estimated hepatocyte subpopulations in *Prmt1* LKO livers by quantitatively deconvoluting bulk RNA-seq data [CIBERSORTx; (*56*)], and observed significant enrichment of daHep population in *Prmt1* LKO livers (fig. S13B). Consistent with the altered transcriptome in *Prmt1* LKO mice (fig. S13C), daHep population in patients showed reduction of *PRMT1*, induction of *PRMT6*, decrease in m^6^A stop transcripts, and increase in direct NRF2 targets (fig. S13D).

**Fig. 7.**
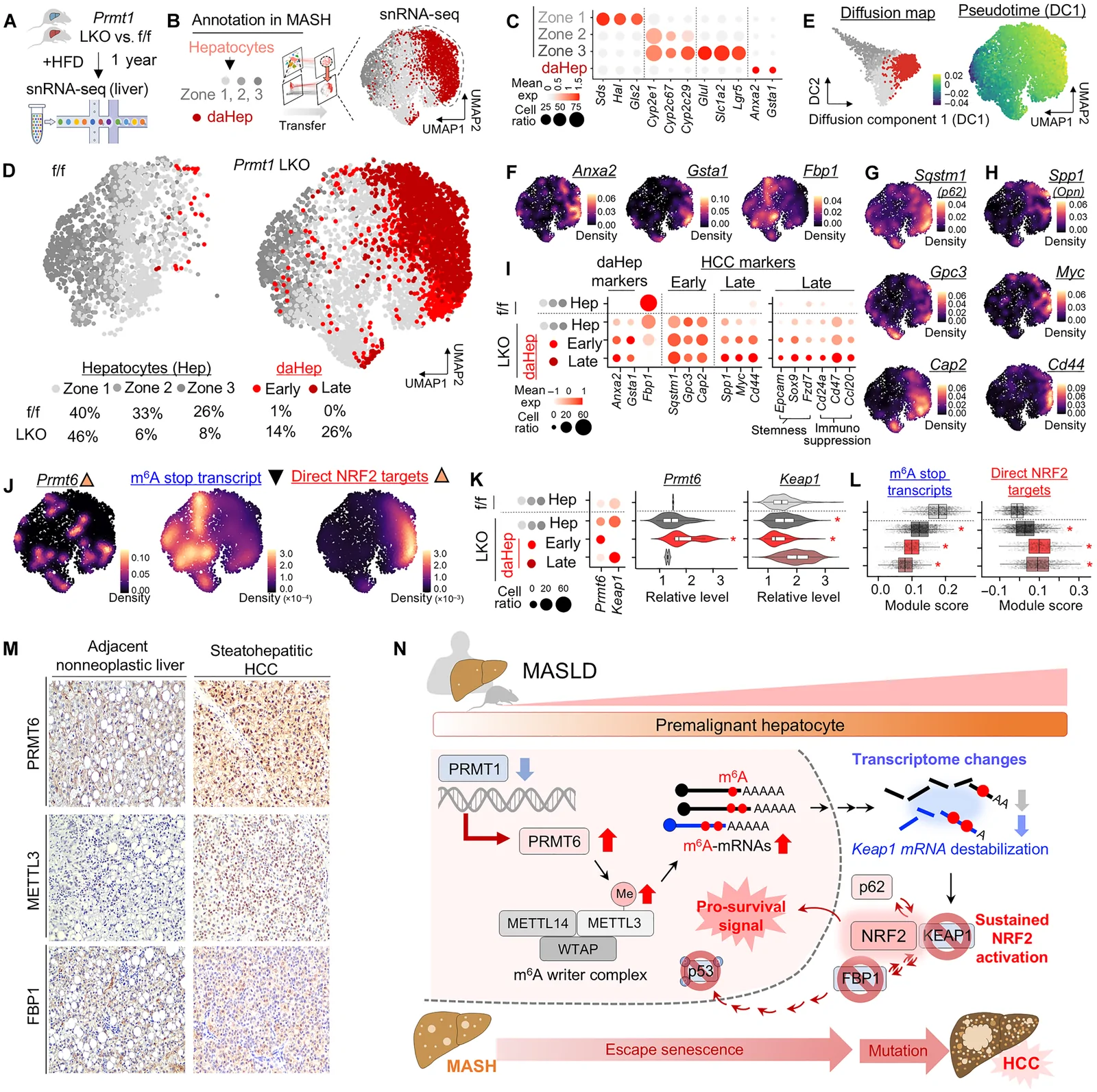
*Prmt1* deficiency produces premalignant daHeps with enhanced NRF2 signaling. (**A**) snRNA-seq experiments for livers of *Prmt1* LKO and f/f mice after 1-year HFD feeding. (**B** and **C**) Reference-based annotation of hepatocyte subpopulations (zones 1, 2, and 3; daHep) by sequential anchor-based label transfer (B) using previously annotated snRNA-seq dataset [mouse MASH models; (*12*)] and their marker gene expression (dot plots; C). (**D** and **E**) Distribution of hepatocyte subpopulations (UMAP; %) in *Prmt1* f/f and LKO (D), where daHeps were further subclustered as early (red) and late (dark red) stages based on diffusion map–based pseudotime analysis (E); diffusion component (DC; E, left) and UMAP clusters with DC1 values (heatmap intensity; E, right) that represent pseudotime (*105*). (**F** to **I**) Relative expression of marker genes associated with daHep (F) and HCC progression (early stage, G; late stage, H) in density plots on UMAP, also represented in dot plots for hepatocytes and daHep subpopulations (*Prmt1* f/f versus LKO; I). Marker genes for late stage of HCC progression include stemness-related and immunosuppression-related genes. (**J**) Relative expression of *Prmt6*, m^6^A stop transcripts and direct NRF2 targets (density plots on UMAP). (**K** and **L**) Relative expression of *Prmt6*, *Keap1* (K), m^6^A stop transcripts, and direct NRF2 targets (module score; L), analyzed for hepatocytes and daHep subpopulations (early and late stages) in *Prmt1* f/f and LKO; dot plots (K, left), violin plots (K, middle and right), and box plots with jitter (L); *P < 0.05, relative to hepatocytes in LKO (K, middle) or in f/f (K, right; L). (**M**) Histological staining of paraffin-embedded sections of adjacent nonneoplastic liver and the steatohepatitic HCC tissues from human patients with HCC; representative image. (**N**) A proposed model for the progression of steatohepatitic HCC. In premalignant daHeps, imbalance between PRMT1 and PRMT6 results in the reduced KEAP1 levels in m^6^A-dependent manner, activating NRF2 signaling.

More definitely, we conducted snRNA-seq in *Prmt1* LKO and f/f livers after 1-year HFD feeding (Fig. 7A and fig. S13, E and F). Because daHeps in mouse MASH models (*12*) also showed induction of *Prmt6*, decrease in m^6^A stop transcripts, and increase in direct NRF2 targets (fig. S13G), annotations including daHep in their snRNA-seq data were used to subcluster hepatocyte populations in *Prmt1* LKO and f/f livers (fig. S13F) via transfer learning (Fig. 7B and fig. S13H) (*57*). After confirming subclusters with marker gene (*10*) expression (Fig. 7C), daHeps in *Prmt1* LKO were found to be markedly increased (40% versus 1%) with reduction of normal hepatocytes [60% versus 99%; particularly in zone2 (midzonal; 6% versus 33%) and zone3 (pericentral; 8% versus 26%); Fig. 7D]. Based on pseudotime analysis using diffusion component (*58*, *59*) (DC1; Fig. 7E and fig. S13I) and marker gene expression (Fig. 7, F to I, and fig. S13J) particularly for early (Fig. 7G) (*60*, *61*) and late HCC (Fig. 7H) (*10*, *62*), daHeps in *Prmt1* LKO could be subclustered into early and late stages in terms of HCC progression (Fig. 7D), wherein stemness-related (fig. S13K) (*63*, *64*) and immunosuppression-related genes (fig. S13L) (*55*, *65*, *66*) were gradually expressed (Fig. 7I). Such observation was concordant with extent of *Prmt6* induction, decrease of m^6^A stop transcripts, and increase of direct NRF2 targets (Fig. 7, J to L). Notably, in daHep populations, *Prmt6* was induced only in the early stage with significant decrease in *Keap1* mRNA, which rather increased in the late stage (Fig. 7K). Such PRMT6-mediated regulation in the limited population of daHep explains marginal changes of *Keap1* mRNA detected in conventional bulk experiments confounded by cellular heterogeneity. As *FBP1* was reduced in daHeps, suppression of *PRMT1* expression and compensatory induction of *PRMT6* initially activates the previously reported NRF2-FBP1-p53 metabolic switch (*10*), which reverses hepatocyte senescence induced by MASH, supports proliferation and affords oncogenic mutations as premalignant state.

Last, we assessed human cases of steatohepatitic subtype HCC to determine the potential involvement of the PRMT6-METTL3-FBP1 axis in steatohepatitic HCC at early stage. We found that nuclear localization of PRMT6 was enhanced in the tumor compared with that in the adjacent nontumor area (Fig. 7M). Furthermore, we observed a concomitant induction of nuclear localization of METTL3 and a reduction of the FBP1, showing that MASLD-associated HCC could be instigated upon the induced expression of PRMT6 and the resultant regulation of the METTL3-FBP1 axis. These data further underscore the importance of PRMT6-dependent regulation of m^6^A modification in the progression of steatohepatitic HCC in mammals.

In summary, we found that the inhibition of PRMT1 and the subsequent compensatory induction of PRMT6 could instigate MASLD-associated HCC by chronic activation of the NRF2 pathway, associated with m^6^A-mediated mRNA degradation of targets including *Keap1* mRNA, contributing to a premalignant state via the NRF2-FBP1-p53 axis. This allows stressed hepatocytes to escape tumor-suppressive senescence and to increase proliferation, enhance stress resistance, and accumulate oncogenic mutations under metabolic and oxidative stress (Fig. 7N). Selective activation of PRMT1 and/or inhibition of PRMT6 in the liver could reduce the persistent NRF2 activity and m^6^A-mediated target mRNA degradation, eventually suppressing the progression of MASH and the MASLD-associated HCC.

## DISCUSSION

Initiated with steatotic lipid accumulation, MASLD progresses to MASH, featured with exacerbated inflammation and fibrosis, and elevates risk of HCC (*5*). However, the MASH-inducing metabolic defects generally drive stressed hepatocyte to enter a nondividing and tumor-suppressive senescence states (*8*, *9*), in part, by triggering DNA damage responses (*10*, *67*–*69*), activation of p53, and resultant expression of senescence-associated target genes (*68*–*70*) including *FBP1* (*10*, *71*), an AKT inhibitor (*11*). Recently, as associated with HCC progression, protumorigenic daHep subpopulation has been identified by snRNA-seq in fibrotic/cirrhotic patient livers (*12*), resembling HCC progenitor cells that present in collagenase-resistant hepatocyte aggregates in DEN-treated mice (*7*). The daHep is produced by prolonged metabolic and oxidative stress, resulting in reversal of senescence and promotion of proliferation via activation of NRF2-FBP1-AKT axis that leads to p53 inactivation (Fig. 7G) (*10*, *12*). Such premalignant states, that preceded oncogenic evolution, were primed in once-senescent p53-activated hepatocytes (*10*), therefore likely occurring by transcriptome changes rather than by DNA mutations. In line with this, dysregulated m^6^A-mediated transcriptome changes, which we discovered in this study as outcome of compensatory arginine methylation, well explain how daHep is primed under metabolic stress for MASLD-associated HCC (Fig. 7G).

In particular, activation of NRF2 in daHep is critical to confer protumorigenic state, in that it induces antioxidant responses that enable the stressed hepatocytes to survive under ongoing stress as well as triggering FBP1 degradation, AKT activation, and resultant p53 degradation to afford subsequent oncogenic mutations (*10*). Although NRF2 is generally activated by degradation of KEAP1 protein under oxidative stress, in part through the increased production and accumulation of p62 aggregates (*61*, *72*), its early and sustained activation, required to prime daHep, could be easily achieved by m^6^A-mediated destabilization of *Keap1* mRNA in combination with other regulatory mechanisms. Compensatory arginine methylation (*20*, *21*), presumably brought by a potential defense mechanism against metabolic defect, directly drives this m^6^A-mediated *Keap1* down-regulation, explaining how NRF2 is activated in response to metabolic stress. In the reciprocal regulation between NRF2 and FBP1, decreased expression of FBP1 is known to activate NRF2 via FBP-TP53-NRF2 axis, additionally accounting for sustained activation of NRF2 (*10*). Moreover, *FBP1* promoter region undergoes hypermethylation in human HCC, mediating long-term repression of FBP1 expression. Therefore, m^6^A-mediated *Keap1* down-regulation could serve as an important mechanism to initiate prolonged NRF2 activation under metabolic dysregulation.

Because its discovery in the past decades or so, PRMT1 has been shown to be involved in the various metabolic pathways. PRMT1 was shown to play a role in the mitochondrial homeostasis in adipose tissues (*20*). On the other hand, cardiomyocyte-specific *Prmt1* knockout mouse displayed the eventual heart failure via the activation of calcium–calmodulin kinase II–associated hypertrophy as well as induction of fibrosis (*22*). We showed that acute knockdown of *Prmt1* in the liver reduced hepatic gluconeogenesis and improved glucose tolerance in mice by inhibiting FOXO1 activity (*23*). Knockout of *Prmt1* ortholog in *Caenorhabditis elegans* reduced the life span of this organism by inhibition of DAF-16, an ortholog of FOXO protein in this species, suggesting that the crucial role of PRMT1 in the control of FOXO family of proteins is evolutionarily conserved between the species (*73*). Specifically, depletion of *Prmt1* in the type 2b muscle in mice led to the compensatory activation of PRMT6-FOXO3 axis, leading to the induction of catabolic signaling in the tissue and the resultant progression of skeletal muscle atrophy (*21*). All of these studies support that PRMT1 expression is essential in metabolic function; therefore, its defective expression should be compensated by expressing its paralog PRMT6 through removal of PRMT1-containing transcription corepressor complex from the *Prmt6* promoter.

The role of PRMT1 in cancers was explored in the recent studies. In non–small cell lung cancers as well as lung cancers, higher PRMT1 expression was observed in human cases, suggesting that PRMT1 might be positively associated with the incidence of tumor progression (*74*, *75*). In HBV-instigated human cases of HCC, PRMT1 expression was also found to be higher, hinting that PRMT1 expression might serve as a biomarker for certain types of human HCC (*76*). Recently, this notion was challenged in that reduced hepatic expression of PRMT1 in mice is causal to the increased incidence of alcohol-induced fatty liver diseases as well as hepatic tumor progression (*25*, *77*). Moreover, PRMT1 protein levels were inversely correlated with ASH-induced HCC in human, showing that the lack of PRMT1 activity could cause the progression of HCC at least in this subtype. Furthermore, HCV was shown to induce hepatocarcinogenesis, in part, via an inhibition of PRMT1 activity (*78*). These data suggest that it is cautious to designate the role of PRMT1 in the progression of HCC in general. Thus, instead of looking into the expression of single PRMT paralogs, it is suggested to investigate the relative expression of PRMT paralogs in parallel in association of diseases including HCC.

Normally, activation of NRF2 pathway is beneficial in protecting the cells and organisms against the oxidative stress by promoting expression of genes involved in the antioxidant genes, and, because of this nature, NRF2 has long been considered as a tumor suppressor (*79*). On the other hand, recent studies indicated that the chronic activation of NRF2 is often associated with the proliferation and stress resistance of the various cancers including HCC, showing a potential role of this factor as a previously unidentified class of oncogene (*10*, *11*, *61*). Unlike the case for alcohol-induced HCC, we did not observe any changes in HNF4α protein levels upon depletion of hepatic *Prmt1* under DIO conditions (fig. S5B). We rather observed increased expression of HNF4α target genes in our mouse models (fig. S5C). These data suggest that, although the lack of hepatic *Prmt1* could induce HCC in both AFLD- and MASLD-dependent manner, the exact signaling cascades for the cancer progression by two pathways are distinct. In this regard, a recent study suggests that the loss of PRMT1 reduced the certain class of antioxidant genes (SOD1 and SOD2) upon alcohol challenge (*77*), while we observed an increased expression of genes involved in the antioxidant pathway in *Prmt1* LKO mice under HFD feeding. It will be interesting to further delineate the differences in the mechanisms for the PRMT1-regulated pathways that are critical for the protection against the AFLD- and MASLD-associated HCC, particularly related to our observation of compensatory arginine methylation that induces activation of m^6^A-*Keap1*-NRF2 axis.

Dysregulation of m^6^A machinery is commonly observed in metabolic diseases and cancers, critically functioning in metabolic processes (*31*) and carcinogenesis (*32*). m^6^A demethylase FTO (*29*) was initially known to be genetically associated with common obesity for its variants (*80*). Activation of METTL3 was reported to induce hepatic lipid accumulation via m^6^A-mediated destabilization of target mRNAs such as *Ppara* (*81*) and also to facilitate liver cancer progression through m^6^A-mediated silencing of target mRNAs including *Socs2* (*51*). In addition to *Keap1*, we also observed significant down-regulation of *Ppara* and *Socs2* as altered m^6^A target transcripts in *Prmt1* LKO mice (fig. S2B) with corresponding MASLD-associated HCC phenotypes.

Previously, asymmetric arginine dimethylation of METTL14 by PRMT1 has been reported to promote m^6^A modification in mouse embryonic stem cells (*38*), where PRMT1 methylated the disordered C-terminal region of METTL14 (*39*). In our hand, both PRMT1 and PRMT6 only marginally confer the asymmetric dimethylation on arginine residues of METTL14 (Fig. 3D). On the other hand, we found that PRMT6, but not PRMT1, could catalyze asymmetric dimethylation on arginine residues of a core catalytic enzyme METTL3, leading to its nuclear accumulation (Fig. 3, B and E). In contrast to tumor promoting roles of METTL3 (*51*), METTL14 acts as both tumor promoter and suppressor (*82*), reported in liver cancer where it attenuated the metastatic potential by regulating microRNA processing (*50*). Considering roles of METTL14 that serves as an allosteric regulator for enhancing METTL3 activity and substrate recognition with degenerate active site (*27*), PRMT6 is more likely to be a major regulator of m^6^A methyltransferase that critically functions in MASLD-associated HCC.

While our data indicate that m6A-dependent regulation of Keap1 mRNA could contribute to the activation of NRF2 pathway upon Prmt1 deficiency in the liver, the precise causal relationship between Keap1 down-regulation and NRF2 activation has not been conclusively established. Specifically, epistasis experiments that would rigorously determine whether Keap1 loss is both necessary and sufficient for NRF2 activation downstream of Prmt1 deficiency have not yet been performed in the current study. Definitive clarification of the hierarchical organization of this pathway will require further studies using genetic rescue approaches and formal epistasis analyses in the future.

In summary, we identified that the reduced expression of PRMT1 and the subsequent induction of PRMT6 in the liver could promote diet-induced steatohepatitic HCC. We propose that the compensatory arginine methylation reprograms m^6^A-mediated hepatic transcriptome and is associated with chronic NRF2 activation, which might prime premalignancy in senescent hepatocytes under defective metabolism. This is presumably brought as an inadvertent outcome during adaptive defenses against metabolic and oxidative stress. Thus, we propose that inhibiting such compensatory arginine methylation and NRF2 activation could be beneficial in reducing the incidence of MASLD-associated HCC.

## MATERIALS AND METHODS

### Primary hepatocyte culture

Primary hepatocytes were prepared from 8- to 12-week-old *Prmt1* f/f or *Prmt1* LKO mice by collagenase perfusion method as previously described (*83*). After the isolation, primary hepatocytes were cultured with M199 medium (Sigma-Aldrich, M4530) including 10% fetal bovine serum (FBS; HyClone, SH30084.03), penicillin-streptomycin solution (50 U/ml), 10 nM dexamethasone, and 23 mM Hepes. After 3 hours of attachment, cells were then treated with various adenoviruses as shown in the figure legends.

### RNA isolation and qRT-PCR

Total RNA was isolated by using an RNeasy plus mini kit (QIAGEN, 4106). cDNA was synthesized by using a GoScript Reverse Transcription System (Promega, A5001) and analyzed by real-time quantitative PCR (qPCR) using a SensiFAST SYBR green mix (Bioline) and CFX connect real-time system (Bio-Rad). The data were normalized to ribosomal gene RPL32. Sequences for gene-specific primers are shown in table S1.

To validate repressive activity of the siRNAs, qRT-PCR was performed for target mRNAs in siRNA-transfected HepG2 cells. Forty-eight hours after the transfection of siMETTL3/14 (each 25 nM siMETTL3 and siMETTL14), siFTO/ALKBH5 (each 24 nM siFTO and siALKBH5), or control siRNA (50 nM NT) using RNAiMax (Invitrogen), large RNAs (>∼200 nt) were isolated using the RNeasy Mini Kit (QIAGEN) through on-column DNA digestion with a ribonuclease (RNase)–free deoxyribonuclease set (QIAGEN, 79254). Then, RT was performed using SuperScript III RT (Invitrogen, 18080051) with anchored oligo(dT) primers. qRT-PCR was conducted using Rotor-Gene Q (QIAGEN) with amfiSure qGreen Q-PCR Master Mix Without Rox (GenDEPOT, Q5600), prepared by OT-2 (Opentrons); METTL3 (*81*) (forward, 5′-CAAGCTGCACTTCAGACGAA-3′; and reverse, 5′-GCTTGGCGTGTGGTCTTT-3′), METTL14 (*81*) (forward, 5′-AGAAACTTGCAGGGCTTCCT-3′; and reverse, 5′-TCTTCTTCATATGGCAAATTTTCTT-3′), FTO (*84*) (forward, 5′-GACCTGTCCACCAGATTTTCA-3′; and reverse, 5′-AGCAGAGCAGCATACAACGTA-3′), and ALKBH5 (*84*) (forward, 5′-ACTGAGCACAGTCACGCTTCC-3′; and reverse, 5′-GCCGTCATCAACGACTACCAG-3′). All the reactions were run in triplicate with the standard two-step cycling protocol. Relative quantification was calculated by the ΔCT method, using glyceraldehyde-3-phosphate dehydrogenase as control (forward, 5′-TGCACCACCAACTGCTTAGC-3′; and reverse, 5′-GGCATGGACTGTGGTCATGAG-3′).

### Protein isolation and Western blot analysis

Proteins were extracted with radioimmunoprecipitation assay buffer [50 mM tris (pH 8), 150 mM sodium chloride, 1% NP-40, 0.5% sodium deoxycholate, 0.1% SDS, and freshly added protease inhibitor cocktail] and analyzed by SDS–polyacrylamide gel electrophoresis followed by Western blot analysis. Proteins were detected using the following specific antibodies: HSP90 (Santa Cruz Biotechnology, sc-13119); PRMT1 (Millipore, 07-404); NRF2 (Cell Signaling Technology, 12721); KEAP1 (Proteintech, 10503-2-AP), asymmetric dimethyl arginine; D6A8 (Cell Signaling Technology, custom order); p62 (Cell Signaling Technology, 5114); NQO1 (Abcam, ab34173); GSTM1 (GeneTex, GTX113448); α-SMA (Abcam, ab7817); PRMT6 (Cell Signaling Technology, 14641); METTL3 (Abcam, EPR18810 and ab195352); METTL14 (Abcam, ab220030); FBP1 (Abcam, EPR4619); AKT (Cell Signaling Technology, 9272S); p-AKT(S473) (Cell Signaling Technology, 9271L); p-AKT(T308) (Cell Signaling Technology, 9275S); and p53 (Santa Cruz Biotechnology, sc-126). All antibodies were used according to the manufacturers’ protocols.

### Animal studies

*Prmt1* heterozygous (EPD0070_1_B01) mice were received from the EUCOMM consortium. *Prmt1* floxed mice were generated by mating *Prmt1* heterozygous mice with FLP transgenic mice, leading to the FLP-mediated excision of FRT-flanked LacZ-neo cassette. *Prmt1* LKO mice were generated by breeding *Prmt1* homozygous floxed (*Prmt1* f/f) mice with albumin-Cre mice, which express Cre recombinase under the control of the albumin promoter. *Prmt1* f/f and *Prmt1* LKO mice fed a NCD were subjected to an GTT after 16-hour fasting and an ITT after 4-hour fasting. To induce hepatic steatosis, mice were fed a HFD (60% kcal Fat) (Research Diets, D12492) for the designated period as indicated in the figure legends. Heterozygotes for Mettl14 (Mettl14^+/−^) mice were described previously (*49*)

All procedures were performed in a specific pathogen-free facility at the Central Laboratory Animal Research Center, Korea University (a 12-hour:12-hour light-dark cycle, maintained at 22°C), based on the protocols that were approved by the Korea University Institutional Animal Care and Use Committee (approval number KUIACUC-2022-0079).

### MCD diet–induced liver fibrosis

Six-week-old male *Prmt1* f/f mice and *Prmt1* LKO mice were fed a MCD diet (Dyets, 518810) for 4 to 8 weeks.

### DEN-induced HCC model

For the diethyl nitrosamine (DEN)–induced HCC model, 25 mg/kg body weight of DEN (Sigma-Aldrich, N0756) was injected intraperitoneally into 20-day old mice. After 2 weeks of DEN administration, mice were fed a HFD (60% kcal of fat) (Research Diets, D12492) for 10 months and euthanized. Visible tumor nodules on the liver surface were counted for each mouse, and sizes of the tumors were also measured.

### Measurement of metabolites

Blood-glucose levels were measured using an automatic glucose monitor (One-Touch). Hepatic and plasma TG contents were measured using an Enzymatic Colorimetric Assay Kit (Wako Chemicals) according to the manufacturer’s instructions

### Hepatic TG extraction

Total hepatic TGs were extracted according to the Folch method (*85*) with minor modification. Briefly, mouse liver was homogenized with chloroform/methanol solution (2:1, v/v) and was centrifuged at room temperature. The supernatant was then washed with 1/5 volumes of 0.9% NaCl and was again centrifuged. After discarding the upper phase, the remaining solution was evaporated under vacuum.

### Immunohistochemistry

Liver tissues were fixed in 10% neutral buffered formalin (Sigma Aldrich, HT501128) and paraffin embedded. Liver sections were deparaffinized with xylene substitute (Merck Millipore, 109843) and rehydrated with decreasing concentrations of ethanol. Heat-induced antigen retrieval was achieved by placing slides in a 700-W microwave oven for 2 min with sections immersed in sodium citrate buffer (pH 6.0) followed by endogenous peroxidase blocking (3% hydrogen peroxide solution and 100% methanol). Nonspecific antibody binding was blocked with 1% bovine serum albumin (BSA), and primary antibodies were incubated at 4°C in a moisture chamber overnight. The appropriate horseradish peroxidase–conjugated secondary antibody was incubated for 1 hour at room temperature. Immunoreactivity was visualized by 3′-diaminobenzidine staining (Vector Labs, SK-4100). Slides were counterstained with Mayer’s hematoxylin (Abcam, ab220365), dehydrated, and mounted in a water-based mounting medium (Thermo Fisher Scientific, 9990402).

### Picrosirius Red staining

Liver sections were deparaffinized with xylene substitute (Merck Millipore, 109843) and rehydrated with decreasing concentrations of ethanol followed by heating in an oven at 60°C for 45 min. The adequate Picrosirius Red solution (Abcam, ab150681) was added onto the slides and incubated for 60 min. Slides were mounted using a xylene-based mounting medium (Fisher Chemical, SP15-500).

### ROS quantification in cells

Liver sections were deparaffinized with xylene substitute (Merck Millipore, 109843) and rehydrated with decreasing concentrations of ethanol. The slides were incubated with dihydroethidium (Invitrogen, D11347) at a final concentration of 5 μM in PBS for 30 min at 37°C in a moisture chamber. Slides were mounted using a xylene-based mounting medium (Fisher Chemical, SP15-500). Fluorescence images were obtained using an LSM800 confocal laser scanning microscope (Carl Zeiss). The images were quantified using image J (Fiji).

### Data analysis of patients with MASLD and MASH

Publicly available transcriptomic datasets of patients with MASLD and MASH were obtained from the Gene Expression Omnibus (GEO) with clinical annotation [GSE162694 (*40*): MASH, F0, *n* = 35; F1, *n* = 30; F2, *n* = 27; F3, *n* = 8; F4, *n* = 12; and GSE135251 (*41*): MASLD, *n* = 51; MASH, F0/F1, *n* = 34; F2, *n* = 53; F3, *n* = 54; F4, *n* = 14; F indicates fibrosis stage]. Raw read counts or normalized expression matrices (TPM) were downloaded, and raw read counts were normalized to RPKM using gene lengths of representative transcripts [Ensembl, MANE Select; (*86*)]. Expression levels of PRMT1 and FBP1 were visualized as box plots within violin plots (lines represent the median and the first and third quartiles, whiskers denote the minima and maxima, and “x” marks the mean) across disease stages (MASLD; MASH, F0-F4) using seaborn (seaborn; https://seaborn.pydata.org/, seaborn.violinplot).

### RNA-seq analysis

Total RNAs from liver tissues of *Prmt1* LKO and f/f mice under 8 weeks HFD (*n* = 3) were prepared with the RNeasy Plus Mini Kit (QIAGEN). To assess the integrity of the total RNA, samples were run on the TapeStation RNA ScreenTape (Agilent Technologies). A library was prepared by the TruSeq mRNA Sample Prep kit (Illumina) according to the manufacturer’s protocol. Indexed libraries were sequenced using the HiSeq 4000 platform (Illumina) by Macrogen Incorporated, Korea.

The raw reads (FASTQ files) were processed into the same limited read length (SE75) within the same batch of experiments and aligned to the mouse genome (mm10) using TopHat2 (*87*) (tophat2 -G–no-novel-juncs -a 4 -g 1–b2-sensitive–segment-length 20) under a supply of RefSeq gene annotations (RefSeqSelect). The abundances of the transcripts were quantified as reads per kilobase of the transcript, per million mapped reads (RPKM) using Cuffdiff (*88*) (cuffdiff --FDR 0.1 –b --compatible-hits-norm) with statistical significance (nominal *P* value). The Bland-Altman (MA) plot was analyzed with indication of significance cutoffs (*P* < 0.01 and *P* < 0.1), examining the distribution of the fold changes [log_2_(*Prmt1* LKO/f/f)] depending on the size of RPKM values (volume; log_2_ RPKM_mean_). The volcano plot analysis was performed by calculating the fold changes and statistical significance [−log_10_(*P* value)] to determine DEGs. All sequence data (FASTQ) were deposited into the Sequence Read Archive (SRA) database (SRP514808).

### GO and GSEAs

GO analysis was conducted by using DAVID (https://davidbioinformatics.nih.gov/) with supply of up-regulated (fold change > 2, *P* < 0.01; *n* = 180) or down-regulated DEG (fold change < −2, *P* < 0.01; *n* = 74). The list of expressed transcripts was used as the background, selected as volume > 0 for DAVID with default parameters unless otherwise indicated. Top five enriched terms in biological process (level 4) and molecular function (direct) were represented with significance (EASE score) and enrichment values.

Functional enrichment analysis was performed by using GSEA program (http://software.broadinstitute.org/gsea); RPKM values were obtained from StringTie v1.3.4d program after mapping the raw reads on mouse genome (mm10) using Hierarchical Indexing for Spliced Alignment of Transcripts (HISAT v2.1.0) by Macrogen Incorporated, Korea. After filtering out genes with no expression (RPKM_mean_ < 1), mouse gene sets were applied to GSEA using Molecular Signatures Database (MSigDB); mouse-ortholog hallmark including inflammatory response (HALLMARK_INFLAMMATORY_RESPONSE; MM3890), EMT (HALLMARK_EPITHELIAL_MESENCHYMAL_TRANSITION; MM3889), and ROS pathway (HALLMARK_REACTIVE_OXYGEN_SPECIES_PATHWAY; MM3895); curated gene sets (M2) including genes up-regulated (SHETH_LIVER_CANCER_VS_TXNIP_LOSS_PAM2; MM534) and down-regulated (SHETH_LIVER_CANCER_VS_TXNIP_LOSS_PAM4; MM1120) in HCC under deficiency of thioredoxin interacting protein (*Txnip*^−/−^); and ontology gene sets (M5) including fatty acid biosynthetic process (GOBP_FATTY_ACID_BIOSYNTHETIC_PROCESS; MM4791) and fibroblast proliferation (GOBP_FIBROBLAST_PROLIFERATION; MM8275). For the analysis of NRF2 targets, we used both direct NRF2 target gene set (*n* = 654) (*43*), selected by overlapping NRF2 chromatin immunoprecipitation sequencing targets and down-regulated transcripts in *Nrf2*^−/−^, and NRF2-dependent gene set (*n* = 469), derived from MSigDB [oncogenic signature genes (C6); NFE2L2.V2, M2870]. For the analysis of daHep marker genes, we used a list of human hepatocyte cluster markers genes as previously reported (*12*), of which 196 genes were assigned to marker genes for the daHep cluster. NES and nominal *P* value were calculated with following parameters; number of permutations = 1000, permutation type = gene_set, metric for ranking genes = log2_Ratio_of_Classes. Of note, NRF2 target genes (*n* = 124), selected by merging the direct NRF2 target genes and *Prmt1*-dependent genes (fold change > 0, *P* < 0.05), were also used for MA plot analyses.

The GO analyses for the m^6^A stop transcripts (*n* = 653) and the METTL14-dependent transcripts (*n* = 315) were performed by using DAVID and the results were visualized using REVIGO program (http://revigo.irb.hr/) with the significantly enriched GO terms (EASE score < 0.1; biological process). In the represented semantic space for clustering, the color and proportional size of circles indicate log_10_(*P* value), derived from EASE score.

### Quantification of m^6^A RNA

Colorimetric detection and quantification of m^6^A was processed by using the m^6^A RNA Methylation Assay Kit (Abcam, ab185912) according to manufacturer’s protocol. Large RNA fraction (300 ng; >∼200 nt), separately purified by using the miRNeasy Mini Kit (QIAGEN, 217084), was subjected to ELISA. After binding RNA samples to the designated wells, m^6^A modification was captured by anti-m^6^A antibody, followed by treatment of secondary antibody with enhancer solution for detection. The absorbance of the sample was measured by spectrophotometer (450 nm, GloMax-Multi Detection System; Promega). The concentration of m^6^A was estimated by comparison with the pre-determined m^6^A standard curve, further represented as relative amount, m^6^A ratio (m^6^A RNA/total RNA; in picograms per nanogram).

### m^6^A RIP-seq

m^6^A RIP-seq was performed as previously described (*48*) with minor modifications (*89*). Using the Dynabeads mRNA Purification Kit (Invitrogen, 61006), polyA^+^ mRNAs (1 to 2 μg) were selected from large RNA (>∼200 nt; 35 to 75 μg), fractionated using the miRNeasy Mini Kit (QIAGEN, 217084) and further fragmented using the NEBNext Magnesium RNA Fragmentation Module (New England Biolabs, E6150S) according to the manufacturer’s protocols. After purification with the RNeasy Mini kit (QIAGEN, 74104) using RLT buffer, the fragmented RNAs were subjected to sequencing library construction either directly (input) or after m^6^A IP. For the quantification of RNA, spectrophotometer (Denovix, DS-11) was used.

For m^6^A IP, magnetic beads were prepared by incubating 5 μg of anti-m^6^A rabbit polyclonal antibody (Synaptic Systems, 202003) with 125 μl of Dynabeads Protein G (Invitrogen, 10004D) in phosphate-buffered saline (PBS; Welgene, LB 004-02; 500 μl of total volume) for 1 hour at room temperature with rotation. After two times of PBS wash, 150 to 500 ng of the fragmented RNA was added to the prepared bead in 200 μl of PXL buffer; PBS (Gibco, 70011044), 0.1% SDS (Invitrogen, AM9820), 0.5% sodium deoxycholate (Sigma-Aldrich, D6750), 0.5% NP-40 (Sigma-Aldrich, 74385), and 60 U of Recombinant RNase Inhibitor (RRI; Takara; 2313A). After 2 hours of incubation at 4°C with rotation, series of wash were applied using five different wash buffers as previously described (*89*); wash 1: ice-cold TE buffer [15 mM tris-HCl (pH 7.5) (Invitrogen, 15567027), 5 mM EDTA (Invitrogen, AM9260G)]; wash 2: ice-cold TE buffer with high detergent [1% Triton X-100 (Sigma-Aldrich, T1020), 1% sodium deoxycholate, 0.1% SDS, and 2.5 mM EGTA (Biosesang, ER2024-001-80)] and high salt [1 M NaCl (Invitrogen, AM9760G)]; wash 3: ice-cold TE buffer with high detergent and extreme high salt (2 M NaCl); wash 4: TE with high detergent and moderate salt (120 mM NaCl); and wash 5: final wash buffer [50 mM tris-HCl (pH 7.5), 1 mM MgCl_2_ (Sigma-Aldrich, AM9530G), 150 mM NaCl, and 0.05% NP-40]. As previously described (*90*), m^6^A RNAs on the beads were competitively eluted two times in 100 μl of IP buffer (PXL) supplemented with 6.7 mM m^6^A (Sigma-Aldrich, M2780) and 40 U of RRI. After incubation for 1 hour at 4°C with continuous shaking, m^6^A RNA was extracted from the supernatant by using an RNeasy Mini kit (QIAGEN, 74104) with RLT buffer.

The cDNA sequencing libraries were constructed using the NEBNext Ultra II Directional RNA Library Prep Kit (New England Biolabs, E7760) following previously reported protocol (*91*). Total amount of the eluted RNA from m^6^A IP (5 μl) was primed by random primer (1 μl) via annealing (5 min, 65°C; hold, 4°C) and then used for first-strand cDNA synthesis (4 μl of 5× buffer, 8 μl of strand specificity reagent, and 2 μl of first-strand synthesis enzyme mix). For input control, 15 to 25 ng of fragmented mRNA was used for this reaction. Sequentially, second-strand cDNA synthesis, purification of double-stranded cDNA using SPRIselect bead (Beckman Coulter, B23318; 1.8×), end prep of cDNA library, adaptor ligation (30-fold dilution) (*91*), and purification of the ligation reaction using SPRIselect bead (0.9×) were performed according to manufacturer’s protocol. Then, part of the sample was used to perform qPCR (Rotor-Gene Q; QIAGEN, 9001550) by addition of SYBR Green I (1:10,000, Invitrogen) to NEBNext Ultra II Q5 Master Mix (New England Biolabs, M0544), determining the number of cycles that were required to reach a linear range of amplification. With the defined optimal cycle number, adaptor ligated cDNAs were amplified by PCR followed by purification using SPRIselect beads (0.9×). The constructed libraries were quantified by a Qubit dsDNA HS assay kit (Invitrogen, Q32854) and Fragment Analyzer (Advanced analytical, M5310AA) with cross-check. Last, all the prepared multiplexed libraries were precisely pooled and sequenced by MiniSeq System (Illumina) in-house 75 single-end (SE75). In case of need, the libraries were sent to the sequencing facility to be sequenced by HiSeq X Ten System (Illumina) in-depth as 150 paired-end reads (PE150). All sequence data (FASTQ) were deposited into the SRA database (SRP514828 and SRP514833)

### Analysis of m^6^A RIP-seq data

The demultiplexed sequencing reads (FASTQ files) were processed into the same read length (SE150) to be comparable within the same batch of replicate experiments, subsequently trimmed for 3′ adaptor sequence using Cutadapt (version 4.1; cutadapt -m 20 -a AGATCGGAAGAGCACACGTC). The trimmed reads were aligned to the mouse transcriptome (RefSeqSelect, derived from UCSC genome browser for mm10) using Bowtie2 (https://bowtie-bio.sourceforge.net/bowtie2) with default parameters followed by filtering out low-quality alignments (MAPQ score ≤ 5). Then, the reads were further selected for ones only in reproducible clusters using CLIPick program (*92*) (CLIPick.reproducible_peaks), where reads were overlapped within at least two biological replicates (BC ≥2).

To identify m^6^A clusters, we followed the analysis method used in the previous study (*47*) with minor modifications. After calculating coverage of compiled reads by summing up the number of reads overlapping each nucleotide using BEDTools (bedtools genomecov), enriched clusters in m^6^A IP at every transcript were selected by comparing read distribution in input control. Initially, the mean coverage for each window was calculated by scanning each transcript using sliding windows of 20 nt with 10-nt overlap (WinIP_mean_ and WinControl_mean_, respectively). After normalizing coverages relative to median coverage value for corresponding transcript (GeneIP_median_ and GeneControl_median_, respectively), WinScores were calculated for every window as log ratio of normalized coverages between m^6^A IP and input control: WinScore = log_2_([WinIP_mean_/GeneIP_median_]/[WinControl_mean_/GeneControl_median_]). After examining different combinations of thresholds considering both relative and absolute m^6^A enrichment, robust m^6^A-enriched windows were refined by using thresholds in WinScore (>2) and WinIP_mean_ (>15), yielding <5% in FDR. FDRs were estimated by comparing the number of selected windows exceeding a set threshold to the number that is obtained if the experiments were computationally reversed with input being treated as immunoprecipitate and vice versa. Distribution of WinScores between two different genotypes was also examined for the windows with absolute m^6^A enrichment (WinIP_mean_ > 15), also indicating the determined WinScore thresholds (>2) for selection. Last, “m^6^A clusters” were defined by merging the m^6^A-enriched windows considering overlaps. For the analysis between *Prmt1* LKO and f/f, we particularly focused on ones located in expressed mRNAs [log_2_(RPKM_mean_) > 0; “m^6^A mRNA cluster”]. Within the m^6^A cluster, middle point of the window that allocates the maximal WinScore, termed “PeakScore,” was defined as a peak position.

For the analyses of m^6^A sequence motifs, RRACH was searched within 200-nt flanking regions from the peak in “unique m^6^A mRNA cluster,” where annotated sequences were available, but there was no other cluster within this flanking region (±200 nt). Positional frequency of the motif was represented as motif ratio, where motif count in 10-nt flanking window (±10 nt) was divided by the total number of clusters so as to compare the distributions between unique m^6^A mRNA clusters (IP) and control clusters (input). The control clusters were defined by selecting input windows exceeding the thresholds (WinScore < −2, WinControl_mean_ > 15) relative to IP. Average motif count for RRACH in the window (20 nt) was also compared with the count for control motifs, RRUGH and RRCAH, for the “unique m^6^A mRNA cluster” in *Prmt1* f/f mice (*n* = 4066) and *Prmt1* LKO mice (*n* = 6760).

Based on the RefSeqSelect annotation, location of the m^6^A mRNA cluster was analyzed using “bedtools intersect” in BEDTools (https://bedtools.readthedocs.io/) program. Allowing multiple counts, the number of the m^6^A mRNA cluster was analyzed and represented as ratio (%) for the given annotations, TSS with 200-nt downstream region (TSS), 5′UTR, coding sequencing (CDS), near stop codon (Stop, ±200 nt), and 3′UTR. Enrichment of annotation in the m^6^A mRNA clusters was calculated for the observed frequency relative to total nucleotides of the annotation in RefSeqSelect. After selecting m^6^A mRNA clusters close to stop codon, where the m^6^A cluster located in mRNAs with >500-nt-long CDS and 3′UTR, positional distribution relative to stop codon was analyzed as ratio (number of clusters per total) depending on genotype (*Prmt1* LKO versus *Prmt1* f/f), compared between m^6^A mRNA clusters (IP) and control clusters (input). Based on this result, “m^6^A stop transcript” was defined ones with m^6^A cluster located within ±300-nt regions from stop codon and used for further analyses.

Analysis of m^6^A clusters for their intersection between two different genotypes accounted at least one nucleotide overlaps between the clusters. For analyzing the overlapping clusters, PeakScores between two different genotypes were compared in the scatter plot particularly for m^6^A stop clusters (*Prmt1* LKO versus f/f), IP clusters (*Mettl14*^+/−^ versus WT), and m^6^A cluster (*Mettl14*^+/−^ versus WT). Relative to WT, the degree of m^6^A enrichment in the given clusters was calculated as ΔPeakScore, where PeakScore of m^6^A cluster in the given genotype was subtracted by the one derived from the overlapped regions in the WT. The volcano plot was also analyzed for ΔPeakScore (*Prmt1* LKO − *Prmt1* f/f) and significance [−log_10_ (*P* value), where *P* value was calculated by comparing WinScore distribution in the m^6^A stop cluster (*t* test, unpaired two-tailed)]. Then, significantly increased m^6^A stop clusters in *Prmt1* LKO mice (*n* = 623; ΔPeakScore > 0 and *P* < 0.05) were reanalyzed for checking their dependency on METTL14 by the volcano plot [ΔPeakScore (*Mettl14^+/−^* − WT) and *P* value]. For analyzing m^6^A IP reads on *Keap1* mRNA, compiled reads from m^6^A IP were visualized after normalizing their coverage values according to read per million mapped reads. All the Python scripts used in these analyses are available at http://clip.korea.ac.kr/m6a/.

### CDF analysis with m^6^A clusters

To check the repressive propensity of transcripts with m^6^A stop clusters, which were identified by m^6^A RIP-seq analysis, CDF depending on fold change [log_2_(*Prmt1* LKO/*Prmt1* f/f)] was analyzed with RPKM values (Cuffdiff) derived from the RNA-seq analyses for the transcripts with different extents of m^6^A modifications (with increment of WinScore threshold in *Prmt1* LKO from default value, >2) near stop codon (±300 nt). Using box plots, we checked the distribution of fold change values depending on WinScore thresholds (>2, >3, and >3.5) of the m^6^A stop transcript, where the corresponding m^6^A clusters were enriched in *Prmt1* LKO (ΔPeakScore > 0) and located near stop codon (±300 nt); the lines represent the median and the first and third quartiles, the whiskers denote the minima and maxima, and “x” marks the mean value. The repressive tendency that positively correlated with WinScore threshold was also confirmed in the Volcano plot analysis for WinScore > 3 considering statistical significance (Cuffdiff; *n* = 3). After narrowing down m^6^A stop transcript (>2, *n* = 3,091; >3, *n* = 946; >3.5, *n* = 435) into ones, of which m^6^A cluster showed METTL14 dependency [ΔPeakScore (*Mettl14*^+/−^ − WT) < 0], the same analyses were conducted for these “METTL14-dependent transcripts” (>2, *n* = 1291; >3, *n* = 374; >3.5, *n* = 151). For CDF analyses, Kolmogorov-Smirnov (KS) test (two-tailed nonparametric) was performed using Scipy (scipy.stats.ks_2samp), relative to the distribution from m^6^A clusters with default threshold in WinScore (>2). With indication of the direct NRF2 target genes showing up-regulation, the changes of transcriptome profile depending on *Prmt1* were analyzed by plotting fold changes [log_2_(*Prmt1* LKO/*Prmt1* f/f) in RNA-seq] and difference in peak height [ΔPeakScore (*Prmt1* LKO − *Prmt1* f/f) in m^6^A RIP-seq], highlighting m^6^A-dependent repressive propensity of m^6^A stop transcripts (*n* = 653) and their METTL14-dependent subset (METTL14-dependent transcripts; *n* = 315) that showed down-regulation in *Prmt1* LKO mice.

### siRNA synthesis

Custom synthesis and modification services from Bioneer (Korea) were used to produce siRNAs with modifications, the quality of which was monitored, reported, and confirmed by the company through mass spectrometry. siRNAs against human METTL3 (*47*), METTL14 (*28*), FTO (*93*), and ALKBH5 (*94*) were synthesized on the basis of previously reported sequences as indicated: siMETTL3 (guide strand, 5′p-AaUCACAACAGAUCCACUdTdT-3′; and passenger strands, 5′-AGUGGAUCUGUUGUGAUAUCC-3′), siMETTL14 (guide strand, 5′p-UuGCUAUUUAACACGGCACdTdT-3′; and passenger strand, 5′-GUGCCGUGUUAAAUAGCAAAG-3′), siFTO (guide strand, 5′p-UcUCACAAGCAGCGGCUAUdTdT-3′; and passenger strand, 5′-AUAGCCGCUGCUUGUGAGACC-3′), and siALKBH5 (guide strand, 5′p-AgAAGUACUUGUUGCGCAGdTdT-3′; and passenger strand, 5′-CUGCGCAACAAGUACUUCUUC-3′); “dT,” thymidine deoxynucleotide; lower case, 2′*-O*-methyl modification. To check the delivery of duplex siRNAs and also prevent passenger strand from loading on RISC complex, 6-carboxyfluorescein was attached to the 5′ end of passenger strands. As a control siRNA, nontargeting miRNA (NT), derived from cel-miR-67 (*C. elegans*–specific miRNA provided as a negative control by Dharmacon), was synthesized (guide strand, 5′p-UCACAØCCUCCUAGAAAGAdTdT-3′; and passenger strand, 5′p-UCUUUØUAGGAGGUUGUGAdTdT-3′); “Ø,” dSpacer (abasic deoxynucleotide). Of note, dSpacer at position 6 or 2′-*O*-methyl at positions 2 was intentionally modified to prevent seed-mediated off-target effects as previously reported (*95*).

### Cell lines and culture

Human HCC Huh7 and HepG2 cell lines were purchased from Korean Cell Line Bank. Immortalized human hepatocyte MIHA cell line was a generous gift from S. W. Nam and originated from J. Roy-Chowdhury (Albert Einstein College of Medicine, Bronx, NY). All of the cell lines were maintained in Dulbecco’s modified Eagle’s medium (Gibco, 22400105; MIHA), modified Eagle’s medium (Gibco, 11095098; HepG2), or RPMI 1640 medium (Gibco, 22400105) supplemented with 10% FBS (Gibco, 12483020), penicillin (100 U/ml), and streptomycin (100 μg/ml; Welgene, LS 202-02). All cells were cultured at 37°C in a humidified incubator with 5% CO_2_. Cell lines have been regularly tested for mycoplasma contamination based on the PCR method (services provided by Cosmogenetech). Authentication of every cell line used in this study was conducted by short tandem repeat DNA Profiling (Korean Cell Line Bank).

### Construction of luciferase reporter vectors

To examine m^6^A-mediated gene expression regulation, we constructed a luciferase reporter vector to contain the m^6^A cluster sequence with repeated RRACH motifs near stop codon of mouse *Keap1* mRNA (mouse m^6^A reporter; 122 nt). For this, we used psiCheck-2 (Promega, C8021) vector and inserted the synthetic m^6^A reporter sequences into its multicloning sites (Xho I/Not I) that locate right after the stop codon of luciferase reporter gene. In addition to mouse Keap1 mRNA, corresponding location of mouse m^6^A reporter in human (146 nt) was identified by using liftover in UCSC genome browser (https://genome.ucsc.edu/cgi-bin/hgLiftOver), narrowed down to contain clustered RRACH motifs (human m^6^A reporter; 123 nt), and also used for luciferase reporter. In parallel, we also constructed control luciferase reporter, of which every RRACH motif was changed to RRUCH. Synthetic duplex oligos (Bionics) used for constructing luciferase reporter vectors are as follows: mouse m^6^A reporter (RRACH: forward, 5′p-tcgagAAGAAAACA AAGCATAAACAGAAAACACAGGGCCGAAGAGGCGGCAGAAGAAGTCATCCCTTCTTCCAGGAAGGGCGACTGGGATGCCTTGTAAAGGACCTTGTGGAAGACCAGAACTCAAAgc-3′; and reverse, 5′p-ggccgcTTTGAGTTCTGGTCTTCCACAAGGTCCTTTACAAGGCATCCCAGTCGCCCTTCCTGGAAGAAGGGATGACTTCTTCTGCCGCCTCTTCGGCCCTGTGTTTTCTGTTTATGCTTTGTTTTCTTc-3′; RRUCH: forward, 5′p-tcgagAAGAAATCA AAGCATAATCAGAAATCACAGGGCCGAAGAGGCGGCAGAAGAAGTCATCCCTTCTTCCAGGAAGGGCGACTGGGATGCCTTGTAAAGGTCCTTGTGGAAGTCCAGATCTCAAAgc-3′; and reverse, 5′p-ggccgcTTTGAGATCTGGACTTCCACAAGGACCTTTACAAGGCATCCCAGTCGCCCTTCCTGGAAGAAGGGATGACTTCTTCTGCCGCCTCTTCGGCCCTGTGATTTCTGATTATGCTTAGTTTTCTTc-3′) and human m^6^A reporter (RRACH: forward, 5′p-tcgagAAAATACAGTCCAATGGGGAGTATCATTGTTTTTGTACAAAAACCGGGACTAAAAGAAAAGACAGCACTGCAAATAACCCATCTTCCGGGAAGGGAGGCCAGGATGCCTCAGTGTTAAAATGAgc-3′; and reverse, 5′p-ggccgcTCATTTTAACACTGAGGCATCCTGGCCTCCCTTCCCGGAAGATGGGTTATTTGCAGTGCTGTCTTTTCTTTTAGTCCCGGTTTTTGTACAAAAACAATGATACTCCCCATTGGACTGTATTTTc-3′; RRUCH: forward, 5′p-tcgagAAAATACAGTCCAATGGGGAGTATCATTGTTTTTGTACAAAATCCGGGTCTAAAAGAAAAGTCAGCACTGCAAATAACCCATCTTCCGGGAAGGGAGGCCAGGATGCCTCAGTGTTAAAATGAgc-3′; and reverse, 5′p-ggccgcTCATTTTAACACTGAGGCATCCTGGCCTCCCTTCCCGGAAGATGGGTTATTTGCAGTGCTGACTTTTCTTTTAGACCCGGATTTTGTACAAAAACAATGATACTCCCCATTGGACTGTATTTTc-3′); underline (RRACH or its control RRUCH motif), lower case (Xho I or Not I cohesive end).

### Luciferase reporter assays

Luciferase reporter assays were conducted as described previously (*89*). Briefly, the constructed luciferase reporter vectors (psi-Check-2; 100 ng per well, 48 wells) were cotransfected with 70 nM siRNAs (70 nM siMETTL3/14: 35 nM siMETTL3 and 35 nM siMETTL14; 70 nM siFTO/ALKBH5: 35 nM siFTO and 35 nM siALKBH5; or 70 nM NT) into Huh7 or MIHA cell lines using Lipofectamine 3000 (Invitrogen) according to the general protocol provided by the manufacturer. Seventy-two hours after the transfection, activity of expressed luciferase was generally measured according to the manufacturer’s protocol. Relative activity (Renilla luciferase activity normalized to firefly luciferase) was initially measured by Dual-Luciferase Reporter Assay System (Promega) with the GloMax-Multi Detection System (Promega) with replicates (*n* ≥ 4) and recalculated into relative m^6^A activity as ratio of relative activity between RRACH m^6^A reporter and RRUCH control reporter.

### dFP reporter assay using flow cytometry

To examine m^6^A-mediated gene expression regulation at the single-cell level, we constructed dFP reporter vectors (*96*), containing the same m^6^A cluster sequences used in the luciferase reporter described above. For this, we used p.UTA.3.0 (a gift from J. Gruber; Addgene, plasmid no. 82447) following the same subcloning method used for psiCheck-2 (Xho I and Not I sites).

The dFP reporter assays were conducted using flow cytometry as described previously (*89*, *97*) with minor modifications. Briefly, the dFP reporter vectors (500 ng per well, 12-well plates) were cotransfected with 70 nM siRNAs (35 nM siMETTL3 and 35 nM siMETTL14; 35 nM siFTO and 35 nM siALKBH5; and 70 nM NT) into Huh7 or MIHA cell lines using Lipofectamine 3000 (Invitrogen) according to the manufacturer’s protocol. Forty-eight hours after transfection, cells were collected in Dulbecco’s PBS (Wellgene, LB 001-01) supplemented with 4% BSA (Bovogen) and 4 mM EDTA (Invitrogen, AM9260G) and analyzed using an Attune NxT flow cytometer equipped with blue (488 nm) and yellow (561 nm) lasers to achieve full excitation of both GFP and RFP. Data acquisition and analysis were performed using Attune software version 3.1 (Thermo Fisher Scientific) and the CytoExploreR package (v1.1.0; https://github.com/DillonHammill/CytoExploreR).

After gating single live cells based on forward scatter and side scatter parameters, cells lacking reporter expression were further excluded on the basis of the basal autofluorescence level estimated from psiCheck-2–transfected cells. To assess the overall distribution of m^6^A reporter activity, the ratio of RFP to GFP fluorescence intensity was calculated per cell as log_2_(RFP/GFP) and normalized by subtracting the median log_2_(RFP/GFP) value of the corresponding RRUCH control population, thereby representing the relative m^6^A-mediated repression as “relative fold change” value at the single-cell level and displayed as box plots.

To further examine reporter activity across a comparable range of vector expression levels, cells were binned into equal intervals based on log_10_(GFP) values as a proxy for vector expression. Within each bin, the mean log_2_(RFP/GFP) value was normalized by subtracting the corresponding mean value from the corresponding RRUCH control of the same bin, yielding the relative fold change in m^6^A reporter activity independent of transfection efficiency. To enrich for cells most sensitive to m^6^A-mediated repression, this analysis was additionally performed using only cells within the lowest 25% of RFP signal values in each GFP expression bin. The box plots depict the median and the first and third quartiles, with whiskers denoting maxima or minima within 1.5 times the interquartile range. The line plots depict the means ± SEM. Statistical significances were tested by the KS test.

### TCGA data analysis of patients with HCC

The results shown herein were based on data generated by TCGA Research Network (www.cancer.gov/about-nci/organization/ccg/research/structural-genomics/tcga) and conducted as previously described (*96*). The clinical datasets within patients with HCC (LIHC; *n* = 424) were downloaded together with matched gene expression profiles (normal tissue, 50; and primary tumor tissue, 371) to retrieve information on RPKM, patient survival, and tumor grade. Therefore, some patients were excluded, such as those with missing gene expression profile, those without follow-up meetings, and those without clinical information. Expression level of the genes (RPKM) related to m^6^A modification—*PRMT1*, *PRMT6*, *METTL3*, and *METTL14*—and of NRF2 targets in HCC were analyzed depending on tumor grade as box plots using Excel or as a violin plot using seaborn (https://seaborn.pydata.org, seaborn.violinplot). In the case of analyzing more than one gene, average RPKM value after quantile normalization (“preprocessCore” in Bioconductor; www.bioconductor.org/) was used.

For PCA, Seurat (*97*) (v5.0.1) in the R program was used for data normalization from read counts (“SCTransform” function), selection of highly variable genes that overlapped with given sets (m^6^A stop transcripts, *n* = 33; and direct NRF2 targets, *n* = 10), and calculation of PCA and display of scatter plot (PC1 and PC2) with information of patient survival year. Of note, box plot analyses for the selected m^6^A stop transcripts (*n* = 33) and direct NRF2 targets (*n* = 10) were represented with average of normalized read count after quantile normalization. For Kaplan-Meier analysis, the packages “survival” and “KMsurv” in Bioconductor were used to compare the survival rates of patients with the high versus the low value of given gene expression or PC (split quartile). The Wilcoxon signed-rank test (two-sided) was performed to check statistical significance considering multiple testing correction.

### CCLE liver cancer cell line data analysis

RNA-seq results from various cancer cell lines were downloaded as read counts or RPKM with corresponding annotations from the CCLE dataset (*n* = 1019; https://sites.broadinstitute.org/ccle/) (*98*). To identify liver cancer cell lines sharing features with *Prmt1* LKO-induced HCC, clustering analysis was performed after merging RNA-seq results from *Prmt1* LKO and *Prmt1* f/f mice, which were generated using StringTie v1.3.4d and converted to human gene symbols using the Ensembl biomart hgnc_homolog database, excluding genes with multiple human orthologs (*99*).

For clustering analysis, the Seurat package in R was used to normalize read counts (SCTransform function), and 2000 highly variable genes were selected and used for PCA (“RunPCA” function). A shared nearest-neighbor graph was constructed using the first 18 principal components (“FindNeighbors” function), and clusters were generated with the Louvain clustering algorithm (“FindClusters” function). For dimensionality reduction, UMAP was calculated and visualized (“RunUMAP” function; dims = 1:18), with the “Primary site” annotation of each cell line or genotype of each mouse displayed in distinct colors. To compare the expression levels of *PRMT1* and *PRMT6*, scatter plots were generated using RPKM values for all cell lines (*n* = 1019) or liver cancer cell lines within the cluster including *Prmt1* LKO mice (*n* = 10). The statistical significance of the correlation was assessed using the Pearson correlation coefficient.

### snRNA-seq data analysis of patients with HCC

snRNA-seq results from liver tissues of 10 patients with HCC were retrieved from the GEO dataset (GSE149614) as a normalized read-count matrix (*55*). Using metadata, hepatocytes (cell type = “hepatocytes”; *n* = 20,782) and protumorigenic hepatocyte clusters (cluster = “15,” “17,” “19,” “27,” “42,” and “47”; *n* = 7285) were filtered as previously described. Cells were stratified into six groups based on PRMT1 expression levels (log_2_-normalized counts: 0 to 0.25, 0.25 to 0.5, 0.5 to 0.75, 0.75 to 1.0, 1.0 to 1.25, and >1.25), and PRMT6 expression levels within these groups were visualized using box plots, excluding cells with no PRMT6 expression; the box plots depict the median and the first and third quartiles, with whiskers denoting maxima or minima within 1.5 times the interquartile range. Of note, PRMT1 expression levels were further visualized as a histogram with bin intervals of 0.05.

snRNA-seq data, describing daHeps, were obtained as processed and annotated Seurat objects from the publicly available dataset (*12*) (https://data.mendeley.com/datasets/w7yh4yjvbw/2), which encompasses liver samples from healthy donors (*n* = 8) and patients with NAFLD (*n* = 7), cirrhosis (*n* = 2), and HCC (*n* = 2); a dataset was originally compiled by integrating publicly available transcriptomic data from GEO (*100*–*102*) (GSE185477, GSE174748, GSE192742, and GSE212046). A total of 78,250 hepatocytes were clustered into normal hepatocytes (normal_Hep), daHeps, and three HCC subtypes (HCC1, HCC2, and HCC3). Expression levels of m^6^A stop transcripts (*n* = 653) and direct NRF2 targets (*n* = 124) were calculated using AddModuleScore function in Seurat and visualized in density feature plots using Nebulosa (*103*) (“plot_density” function) to recover dropout or low-expression signals.

### Bulk RNA-seq deconvolution

Based on scRNA-seq results that described daHep population (*12*), we deconvoluted bulk RNA-seq data from *Prmt1* LKO and *Prmt1* f/f mice to quantify hepatocyte subtypes using CIBERSORTx (*56*). Initially, RNA-seq results in *Prmt1* LKO and *Prmt1* f/f mice were aligned and quantified using StringTie (v1.3.4d), followed by annotation with human orthologs using Ensembl Biomart (gene symbol) as described in the “CCLE liver cancer cell line data analysis” section. A signature matrix, a reference table of cell-type–specific gene-expression profiles, was generated from snRNA-seq dataset (117,121 nuclei) (*12*) using only genes shared with the bulk RNA-seq (*n* = 15,204) for previously defined cell types (normal_Hep, daHep, HCC1, HCC2, and HCC3 for hepatocytes; mesenchymal, endothelial, lymphoid, BECs, and myeloid for nonhepatocytes). CIBERSORTx was executed via Docker (cibersortx/fractions) with “--single_cell TRUE --rmbatchSmode TRUE” options to perform S-mode batch correction. To avoid memory overload, the snRNA-seq matrix was randomly partitioned into 10 equal subsets in R by simple random sampling [sample(replace = FALSE)], and each subset was processed separately by CIBERSORTx. For each sample, hepatocyte subtype fractions from all 10 runs were averaged to obtain a single representative value. These representative fractions were compared between *Prmt1* LKO and *Prmt1* f/f mice using two-sided *t* tests, and relative ratios were calculated by dividing each *Prmt1* LKO mice fraction by the mean *Prmt1* f/f mice fraction; the mean and SD of these relative ratios were represented.

### Single-nucleus RNA sequencing

Single-nucleus isolation and construction of snRNA-seq libraries were performed through custom services from ROKIT GENOMICS Inc. For single-nucleus isolation, ∼100 mg of frozen liver tissue from each *Prmt1* LKO or *Prmt1* f/f mouse was used; after liver collection, tissues were finely minced, flash frozen in liquid nitrogen, and stored at −80°C until use, and, when HCC lesions were present, particularly in *Prmt1* LKO mice, lesion and adjacent nonlesion regions were dissected separately before freezing and proportionally pooled immediately before homogenization. The tissue was homogenized with a pestle in ice-cold Nuclei EZ Lysis Buffer (Sigma-Aldrich, NUC101) supplemented with RNase inhibitor (0.2 U/μl). The nucleus suspension was passed through a 30-μm strainer to remove cell debris and undigested tissue fragments, and washed twice with Ca^2+^- and Mg^2+^-free PBS containing 1% BSA and RNase inhibitor (0.2 U/μl) by centrifugation at 500*g* for 5 min at 4°C. Debris was further removed using Anti-Nucleus MicroBeads (Miltenyi Biotec, 130-132-997), and the purified nuclei were gently resuspended in cold Ca^2+^- and Mg^2+^-free PBS containing 1% BSA and RNase inhibitor (0.2 U/μl) and filtered through a 30-μm Pre-Separation Filter (Miltenyi Biotec, 130-041-407). The nuclei were counted using a LUNA-FX7 Automated Fluorescence Cell Counter (Logos Biosystems) with acridine orange and propidium iodide staining (Logos Biosystems, F23001).

The snRNA-seq libraries were generated according to the Chromium GEM-X Single Cell 3′ Reagent Kits v4 protocol (10x Genomics, CG000731) using the Chromium X platform and GEM-X Single Cell 3′ Kit v4 (10x Genomics, 1000691). Briefly, nucleus suspensions were loaded to recover 10,000 nuclei per sample and partitioned with Single Cell 3′ Gel Beads and Partitioning Oil in a GEM-X Single Cell 3′ Chip Kit v4 (10x Genomics, 1000690) to generate single-nucleus gel bead-in-emulsions. Polyadenylated RNAs were barcoded and reverse transcribed within droplets, followed by cDNA amplification by PCR. Gene expression libraries were constructed by sequential enzymatic fragmentation, end repair, A-tailing, adaptor ligation, and index PCR. Purified libraries were quantified by qPCR using the KAPA Library Quantification Kits (Roche, 07960140001) and assessed using the 4200 TapeStation system (Agilent Technologies). The libraries were sent to the sequencing facility for deep sequenced on the NovaSeq X Plus platform (Illumina) with 150–base pair paired-end reads (PE150). All sequence data (FASTQ) were deposited into the SRA database (SRP684930).

### Analysis of snRNA-seq data

Demultiplexed reads (FASTQ file), including index reads, were processed using Cell Ranger (v9.0.1) with the mouse reference transcriptome (refdata-gex-GRCm39-2024-A, 10x Genomics). Filtered gene–barcode matrices generated by Cell Ranger were imported into R (v4.4.1) using the Seurat package (v5.3.1) (*97*) with Read10X_h5 and converted into Seurat objects with CreateSeuratObject. Quality-control (QC) filtering was applied based on the number of detected genes (nFeature_RNA) and the percentage of mitochondrial transcripts calculated using PercentageFeatureSet. Only nuclei with more than 500 and fewer than 3000 detected genes and mitochondrial transcript proportions below 5% were retained, as previously described (*12*).

For reference-based cell-type and hepatocyte-subtype annotation, we applied sequential anchor-based label transfer (*57*) using reference annotations in a previous snRNA-seq dataset (Mendeley Data, doi: 10.17632/w7yh4yjvbw.2), derived from MASH mouse model (*12*). For whole-liver annotation, cell-type labels were transferred using the “cell_type” metadata provided in “Processed_mouse_snRNA-seq_data/All_cells.rds” (28,692 genes × 40,748 nuclei), including hepatocytes, mesenchymal cells, endothelial cells, biliary epithelial cells, myeloid cells, plasmacytoid dendritic cells, B cells, T/natural killer cells, and mesothelial cells. The QC-filtered Seurat objects from individual samples (*Prmt1* LKO and f/f) were merged, and layers in the merged object were collapsed into a single RNA layer using JoinLayers, followed by performing normalization with NormalizeData, variable feature selection with FindVariableFeatures, scaling with ScaleData, and PCA with RunPCA. Transfer anchors between the whole-liver reference and the query object were then identified using FindTransferAnchors, with the precomputed PCA of the reference object used as the reference reduction (reference.reduction = “pca,” dims = 1:30), yielding 3693 anchors. Query cell labels were subsequently assigned using TransferData, and the predicted labels were added as metadata.

Hepatocyte populations in the first-round annotation were subsetted and subjected to a second round of label transfer for hepatocyte subtype annotation using the cell_type metadata in “Processed_mouse_snRNA-seq_data/Hep.rds” (28,692 genes × 12,540 nuclei), including zone 1 (periportal) hepatocytes, zone 2 (midzonal) hepatocytes, zone 3 (pericentral) hepatocytes, and daHep. Following preprocessing of the hepatocyte reference object in the same manner as the query object, transfer anchors were identified between the reference and the hepatocyte query subset using FindTransferAnchors with the reference PCA (reference.reduction = “pca,” dims = 1:30), yielding 2297 anchors, and subtype labels were assigned using TransferData. For joint visualization of the two conditions, we applied batch correction using the mutual nearest neighbor (MNN) method (*104*) implemented in RunFastMNN with 3000 features and generated UMAP embeddings from the first 30 MNN dimensions. Transferred annotations were further supported by the expression patterns of previously described normal hepatocyte zonation and daHep marker genes (*10*). Zone 1 hepatocytes were characterized by *Sds*, *Hal*, and *Gls2*; zone 2 hepatocytes by *Cyp2e1*, *Cyp2c67*, and *Cyp2c29*; zone 3 hepatocytes by *Glul*, *Slc1a2*, and *Lgr5*; and daHep by *Anxa2* and *Gsta1*.

For diffusion map–based pseudotime analysis (*59*), diffusion components were computed from the MNN-corrected normalized expression matrix using the destiny package (*58*). Briefly, 500 highly variable genes were selected with FindVariableFeatures, and the corresponding expression values were used as input for DiffusionMap using 50 principal components (n_pcs = 50). The first diffusion component (DC1) was used as a pseudotime variable, as previously described (*105*), and DC1 and DC2 were used for diffusion map visualization. For daHep subclustering, a shared nearest-neighbor graph was constructed from DC1 to DC10 using FindNeighbors, and clusters were identified by Louvain clustering with FindClusters. daHep subclusters were then annotated as early daHep or late daHep on the basis of the expression patterns of daHep markers (*Anxa2* and *Gsta1*) (*10*), together with previously reported markers associated with early HCC (*Sqstm1*, *Gpc3*, and *Cap2*) (*60*, *61*) or late HCC (*Spp1*, *Myc*, and *Cd44*) (*10*, *62*). daHep subclusters were further supported by the expression patterns of daHep signature genes, HCC marker genes, stemness-related and immunosuppression-related genes, genes used for daHep signatures (*Anxa2*, *Abcc4*, *Krt8*, *Pvt1*, *Robo1*, *Tinag*, *Cyp2a4*, *Sybu*, *Sytl5*, *Apoa4*, and *Zfp704*) (*12*), HCC markers [*Afp*, *Ly6d*, *Gpc3*, *Myc*, *Cd44*, and *Sqstm1* (p62)] (*10*, *106*), stemness-related genes (*Epcam*, *Sox9*, and *Fzd7*) (*63*, *64*), and immunosuppression-related genes (*Cd24a*, *Cd47*, and *Ccl20*) (*55*, *65*, *66*).

Expression patterns of individual genes were visualized using density plots with Nebulosa and dot plots with Seurat. To assess broader gene-set activity, module scores for m^6^A stop transcripts (*n* = 653), direct NRF2 targets (*n* = 124), daHep signatures (*n* = 11), and HCC markers (*n* = 6) were calculated using AddModuleScore in Seurat and visualized density plots (method = “wkde”). Expression of *Prmt6*, *Keap1*, the m^6^A stop transcripts, and the direct NRF2 targets was further visualized using violin plots or box plots with jittered points, excluding cells with no detected counts for each gene; the box plots depict the median and the first and third quartiles, with whiskers denoting maxima or minima within 1.5 times the interquartile range. Statistical differences were tested by KS tests.

### Clinical study

The patients diagnosed with the steatohepatitic subtype of HCC associated with metabolic dysfunction underwent curative hepatic resection between 2010 and 2012 at Severance Hospital, Yonsei University Health System. There was a total of eight patients, with an average age of 68.6 ± 8.63 years (means ± SD), including two men and six women. No patients were administered any preoperative treatment. The pathological diagnosis of steatohepatitic subtype of HCC was performed according to the *Digestive System Tumours: WHO Classification of Tumours* (fifth edition) (*107*). Metabolic dysfunction was diagnosed on the basis of established cardiometabolic criteria (*4*), and all eight patients had metabolic syndrome, and five of them had concurrent chronic B viral infection. The formalin-fixed, paraffin embedded tissue specimens of tumor and their pair-matched nontumor liver tissues were used in this study. For the immunohistochemistry, following antibodies were used; PRMT6 (Proteintech, 15395-1-AP), METTL3 (Abcam, EPR18810), and FBP1(Abcam, EPR4619). The Institutional Review Board of Severance Hospital approved this study (no. 4-2017-0973) and waived the requirement for informed consent.

### Statistical analysis

Data were analyzed using Prism (GraphPad 8.0, GraphPad Software), Scipy (www.scipy.org/), Bioconductor (www.bioconductor.org/), and Excel and were presented as means ± SEM or SD. Statistical significance was determined using either Student’s *t* test (when comparing values between two conditions) or one-way analysis of variance (ANOVA) and post hoc Tukey’s test (when comparing values among more than two conditions). We used Wilcoxon signed-rank test (two-sided) for patient survival analyses and KS test (two-tailed nonparametric) for CDF analyses. *P* values of <0.05 were considered statistically significant.
