# Supplementary material for: Dysregulated m6A via compensatory arginine methylation primes premalignancy in metabolic dysfunction–associated steatotic liver disease
Source: Sci Adv. 2026 Sep 9;12(37):eaec5094. doi: 10.1126/sciadv.aec5094 (PMC13557090; doi:10.1126/sciadv.aec5094)
Supplement: Supplementary file 1 [file sciadv.aec5094_sm.pdf]

Science Advances
AAAS

Supplementary Materials for

Dysregulated m^{6}A via compensatory arginine methylation primes premalignancy in metabolic dysfunction–associated steatotic liver disease

Geun-Woo D. Kim *et al.*

Corresponding author: Seung-Hoi Koo, koohoi@korea.ac.kr; Sung Wook Chi, chi13@korea.ac.kr

Sci. Adv. **12**, eaec5094 (2026)
DOI: 10.1126/sciadv.aec5094

This PDF file includes:

Figs. S1 to S13
Table S1

Supplementary Figures

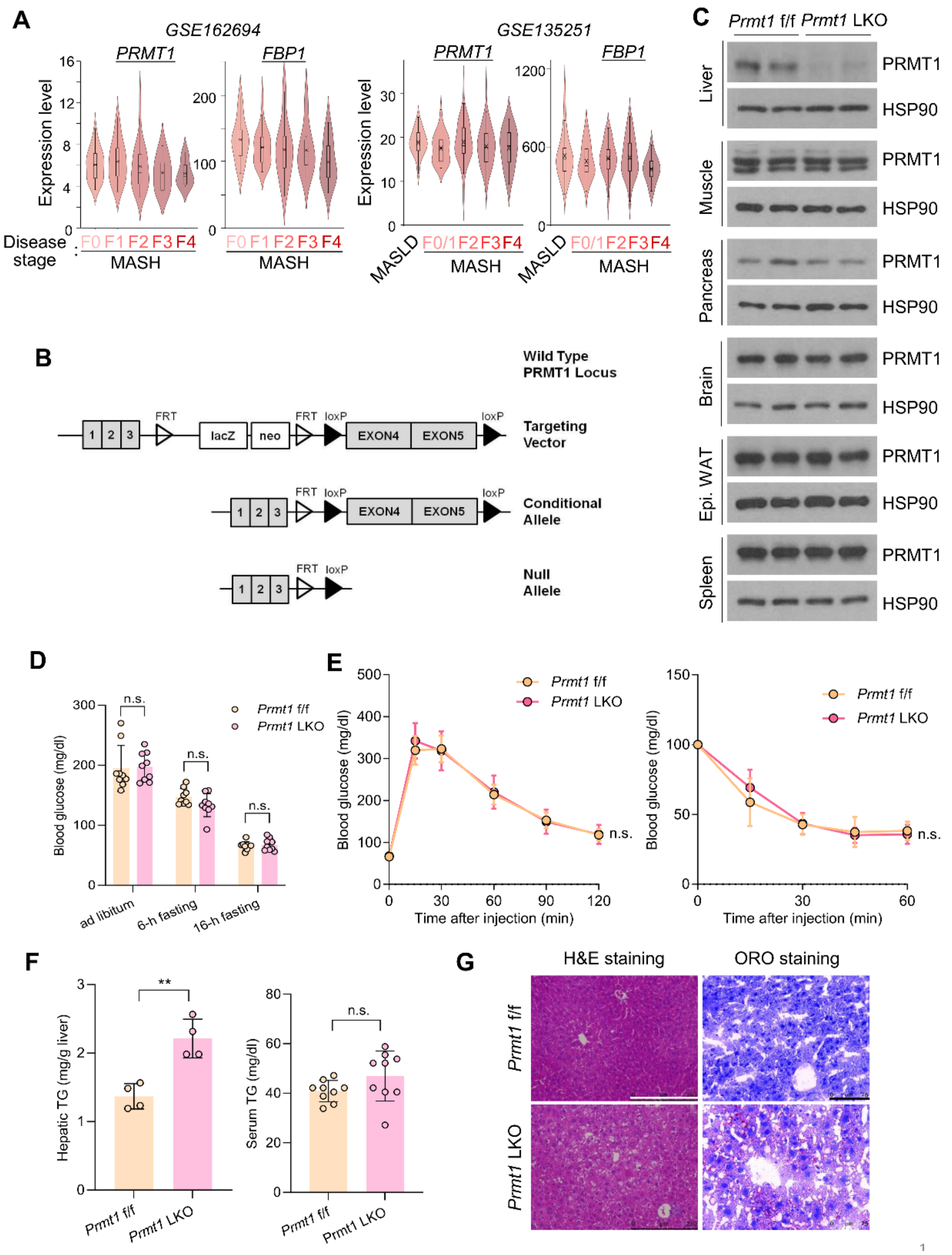

Figure S1. Liver-specific *Prmt1* knockout promotes accumulation of TG in the liver. **A.** Average expression levels of *PRMT1* and *FBP1* in MASLD and MASH patients from the GSE162694 (left panel; *n* = 112) *(40)* and GSE135251 (right panel; *n* = 206) *(41)* across different disease stages (MASLD; MASH with fibrosis stages F0, F1, F2, F3 and F4), represented as box plots within violin plots (lines represent the median, first and third quartiles, whiskers denote the minima and maxima, and ‘x’ marks the mean). **B.** Schematic diagram showing the targeting strategy for generating liver-specific *Prmt1* knockout mice. **C.** Protein levels of PRMT1 in metabolic tissues showing the specificity of liver-specific depletion of *Prmt1* in 6 h-fasted, 8-week-old mice under normal chow diet (NCD). **D.** Ad

libitum (fed), 6-h fasting or 16 h-fasting blood glucose levels from either *Prmt1* f/f mice or *Prmt1* LKO mice under NCD (male 8-week-old mice. n = 9 mice per group). **E.** Glucose tolerance test (GTT, left) and insulin tolerance test (ITT, right) showing effects of chronic depletion of hepatic *Prmt1* in mice under NCD on glucose metabolism (n = 9 mice per group). **F.** Hepatic TG contents (left) and Serum TG contents (right) from *Prmt1* f/f mice or *Prmt1* LKO mice under NCD (male 8-week-old mice. left, n = 4 mice per group or right, n = 9 mice per group). **G.** H&E staining (left) and oil red o (ORO, right) staining of liver tissues from either *Prmt1* f/f mice or *Prmt1* LKO mice under NCD (male 8-week-old mice). Data represent three independent experiments (n = 3 mice per group). Scale bars: 250µm (x200) (for H&E staining), 75 µm (x400) (for ORO staining). All values represent mean ± SEM. ** $P < 0.01$. n.s.: non-specific.

A

Sample name	Total read	Mapped read	%	RefSeq coverage	%
f/f-1	32,842,335	31,800,115	96.8	25,919	74.3
f/f-2	39,741,596	38,255,639	96.3	26,144	75
f/f-3	33,252,833	32,104,752	96.5	25,737	73.8
Prmt1-LKO-1	33,036,308	32,015,370	96.9	26,669	76.5
Prmt1-LKO-2	31,252,651	30,220,222	96.7	26,322	75.5
Prmt1-LKO-3	33,788,021	32,778,653	97	26,321	75.5

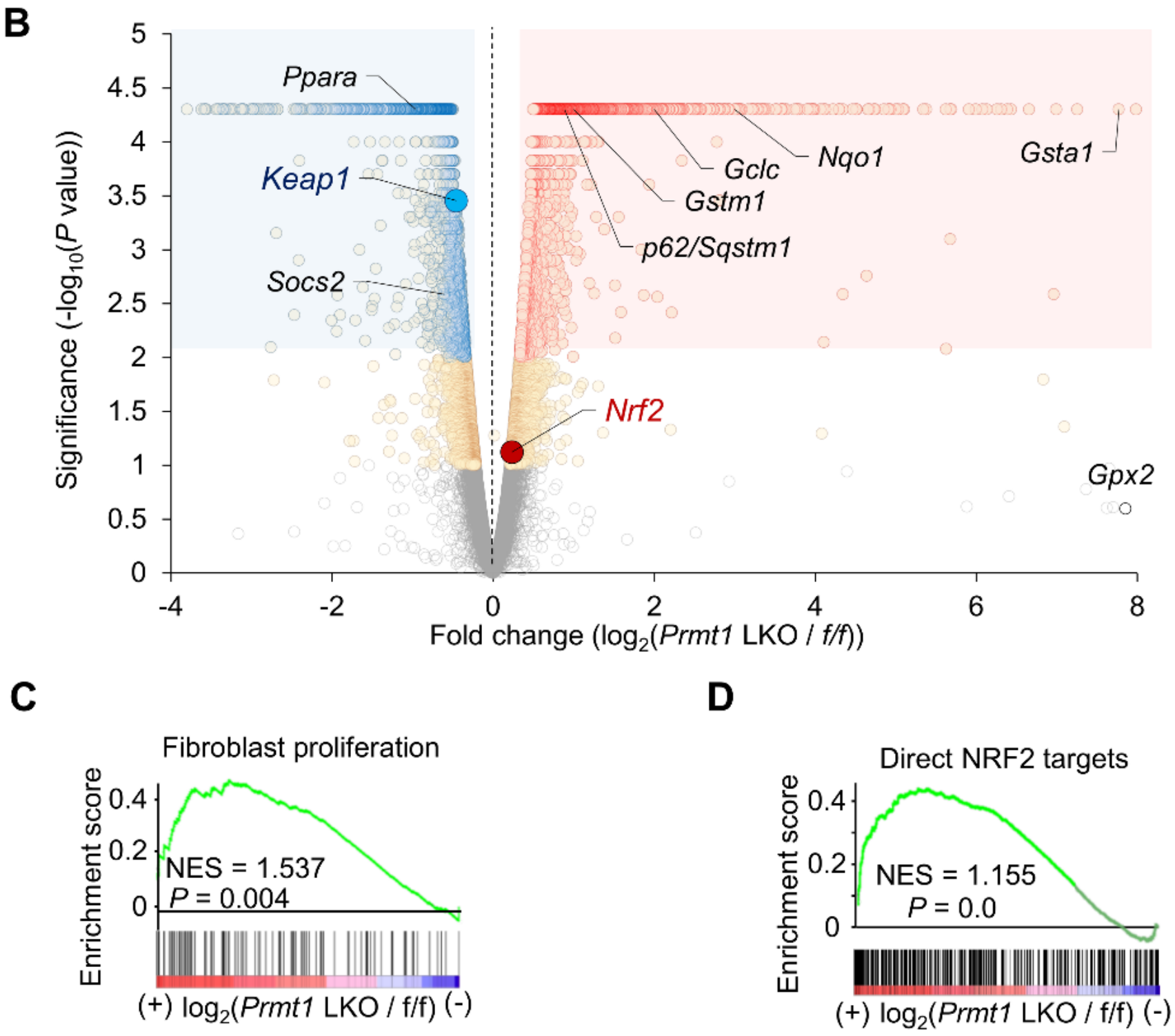

Figure S2. Transcriptome profiles of livers from *Prmt1* f/f mice and *Prmt1* LKO mice. **A.** Summary of mapping results for RNA-seq, performed in livers from *Prmt1* f/f mice or *Prmt1* LKO mice under 8-week HFD (n=3 mice per group). **B.** RNA-seq analysis conducted in livers from *Prmt1* f/f mice or *Prmt1* LKO as in Fig. 2a except representing as the Volcano plot; relative to *Prmt1* f/f (n = 3 individual samples) with statistical significance (derived from Cuffdiff; $P < 0.01$); faint red circle, upregulation; blue circle, downregulation; orange circle, $P < 0.1$. *Keap1* and *Nrf2* were highlighted. Validated genes using qRT-PCR in Fig 2F and western blots in Fig 2G are also displayed (*Gstm1*, *Gsta1*, *Gclc*, *Gpx2*, *Nqo1* and *p62/Sqstm1*). **C, D.** GSEA results with functional terms, "fibroblast proliferation" (**C**) in MSigDB (MM8275) and "direct NRF2 targets" (**D**), which were selected by overlapping NRF2 ChIP-Seq targets and downregulated transcripts in *Nrf2*$^{-/-}$ (n = 654) *(43)* and represented as distribution of enrichment score depending on fold change ($\log_2$ (*Prmt1* LKO / *Prmt1* f/f)); Normalized enrichment score (NES) and nominal P value indicate significant positive correlation ($P < 0.01$, NES > 1) with fibroblast proliferation.

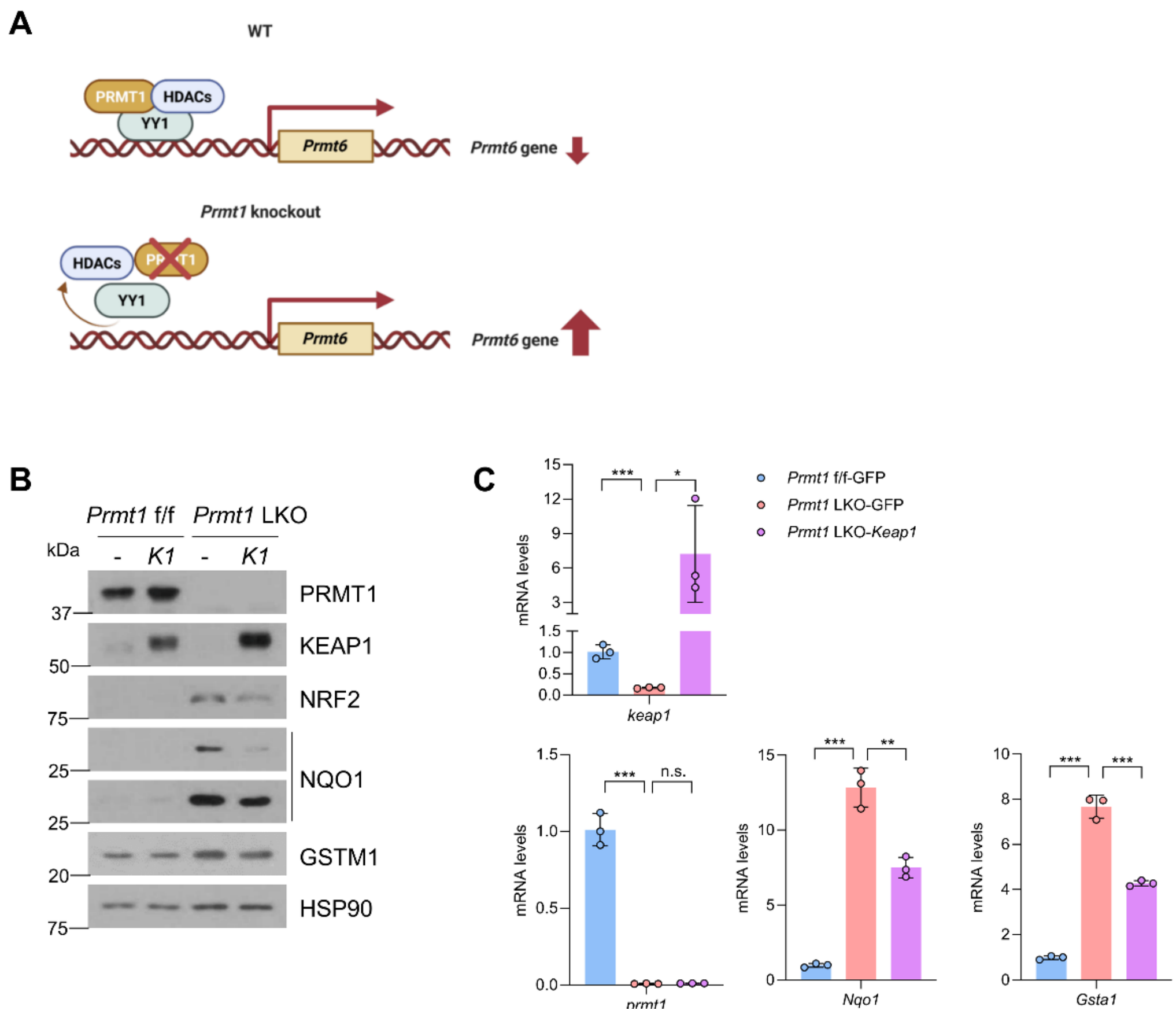

Figure S3. *Prmt1* deficiency induces an increased expression of NRF2.
A. Transcriptional activation of *Prmt6* upon depletion of *Prmt1* based on the previous study *(21)*. **B.** Western blot analysis showing expression of PRMT1, KEAP1, NRF2 and its downstream target proteins in the primary hepatocytes from *Prmt1* f/f or *Prmt1* LKO mice that were infected with Ad-GFP or Ad-*Keap1*. A representative data was shown. **C.** The mRNA levels of NRF2 target genes were measured by qRT-PCR in the primary hepatocytes from *Prmt1* f/f or *Prmt1* LKO mice that were infected with Ad-GFP or Ad-*Keap1*. Values represent mean ± SEM (n=3 per group). * $P < 0.05$, ** $P < 0.01$ and *** $P < 0.001$. n.s.: non-specific.

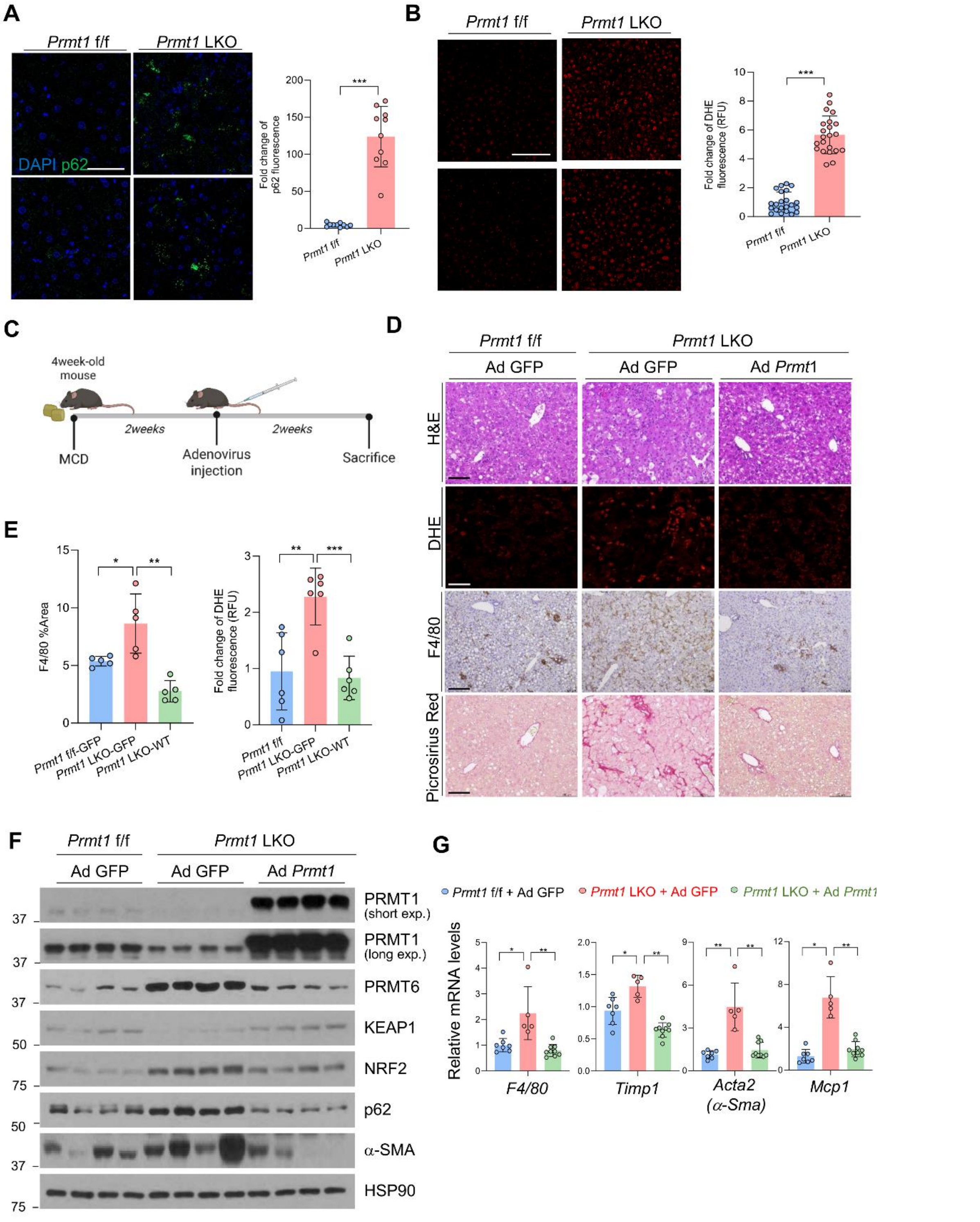

Figure S4. *Prmt1* deficiency leads to an accumulation of ROS in the liver.

A. Histological staining of p62 (green) and DAPI (blue) in liver tissues from *Prmt1* f/f or *Prmt1* LKO mice under 8-week HFD (left). Data represent three independent experiments (*n* = 3 mice per group). Scale bar: 50 µm (x200). Quantification of p62 level was shown (right). **B.** Dihydroethidium (DHE) staining of liver tissues from *Prmt1* f/f or *Prmt1* LKO mice under 8-week HFD (left). Data represent three independent experiments (*n* = 3 mice per group). Scale bar: 50 µm (x200). Quantification of DHE staining was shown (right). **C.** Schematic diagram for the mouse experiment. **D.** Histological staining of paraffin-embedded sections of liver

tissues from 4-week MCD diet-fed mice. Data represent three independent experiments (*n* = 3 mice per group). Scale bar: 100 μm (x200) (for H&E staining), 100 μm (x200) (for Picrosirius Red staining), 100 μm (x100) (for F4/80 staining) and 25 μm (x400) (for DHE staining). **E.** Quantitation of F4/80 (left) and DHE (right) in Fig S4D. **F.** Expression of PRMTs, KEAP1, NRF2 and its downstream target proteins, as well as α-SMA in the liver tissues from *Prmt1* f/f mice (with Ad-GFP infection), *Prmt1* LKO mice (with Ad-GFP infection) or *Prmt1* LKO mice (with Ad-*Prmt1* infection) under 4-week MCD diet (*n* = 4 per group). **G.** The mRNA levels of genes associated with inflammation and fibrosis were measured by qRT-PCR in the liver tissues from *Prmt1* f/f mice (with Ad-GFP infection, *n* = 7), *Prmt1* LKO mice (with Ad-GFP infection, *n* = 5) or *Prmt1* LKO mice (with Ad-*Prmt1* infection, *n* = 7) under 4-week MCD diet. Values represent mean ± SEM. **P* < 0.05, ** *P* < 0.01 and *** *P* < 0.001. Fig. S4**C**: Created in BioRender. Kim, S. (2026) https://BioRender.com/o2vy791.

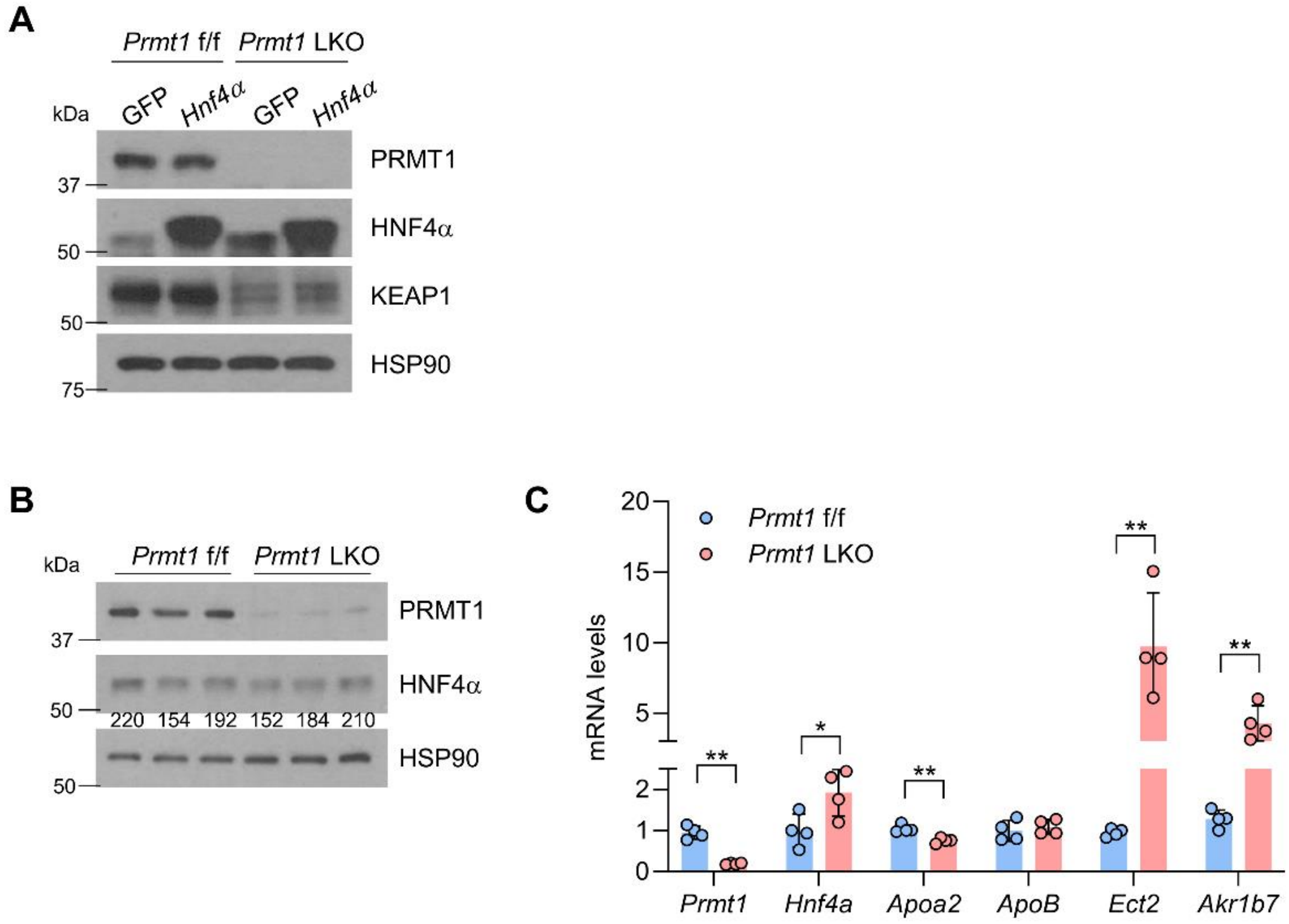

Figure S5. Depletion of *Prmt1* does not alter HNF4a-mediated signaling in the liver. **A.** Western blot analysis showing effects of HNF4a on KEAP1 expression in the primary hepatocytes from *Prmt1* f/f or *Prmt1* LKO mice that were infected with Ad-GFP or Ad-*Hnf4a*. A representative image was shown. **B.** Western blot analysis showing expression of HNF4a in the livers from *Prmt1* f/f mice or *Prmt1* LKO mice under 12-week HFD (male 8-week-old mice. n = 3 mice per group). **C**. qRT-PCR analysis showing expression of *Hnf4a* and its downstream target genes in the livers from *Prmt1* f/f mice or *Prmt1* LKO mice under 12-week HFD (male 8-week-old mice. n = 4 mice per group). Values represent mean ± SEM. * $P < 0.05$ and ** $P < 0.01$.

A

Sample name	Sample type	Exp	Total read	Mapped read	%	Reproducible read (BC≥2)
f/f-1	f/f liver mRNA	input	18,226,124	8,585,882	47.1	
f/f-2	f/f liver mRNA	input	24,216,905	8,465,505	35	26,398,107
f/f-3	f/f liver mRNA	input	21,641,606	9,370,466	43.3	
Prmt1-LKO-1	LKO liver mRNA	input	19,094,159	7,398,921	38.8	
Prmt1-LKO-2	LKO liver mRNA	input	21,042,204	7,700,194	36.6	21,367,939
Prmt1-LKO-3	LKO liver mRNA	input	16,376,132	6,294,729	38.5	
f/f-IP-1	f/f liver mRNA	m^{6}A IP	20,673,540	11,621,595	56.3	
f/f-IP-2	f/f liver mRNA	m^{6}A IP	33,768,535	16,068,058	47.6	40,353,089
f/f-IP-3	f/f liver mRNA	m^{6}A IP	23,194,594	12,685,416	54.7	
Prmt1-LKO-IP-1	LKO liver mRNA	m^{6}A IP	20,394,169	10,371,398	51	
Prmt1-LKO-IP-2	LKO liver mRNA	m^{6}A IP	17,806,896	8,764,364	49.2	28,296,664
Prmt1-LKO-IP-3	LKO liver mRNA	m^{6}A IP	18,770,380	9,186,571	49	

Sample name	m^{6}A cluster	m^{6}A mRNA cluster (log$_2$(volume)>0)	Unique m^{6}A mRNA cluster (with ±200nt sequence & no other cluster within ±200nt)	m^{6}A stop cluster (m6A mRNA cluster within ±300nt of stop codon
f/f	3,021	**3,003**	**2,426**	**309**
PRMT1-LKO	5,994	**5,980**	**4,170**	**672**
f/f-IP	7,508	**6,893**	**4,066**	**2.030**
PRMT1-LKO-IP	13,938	**13,345**	**6,760**	**4,672**

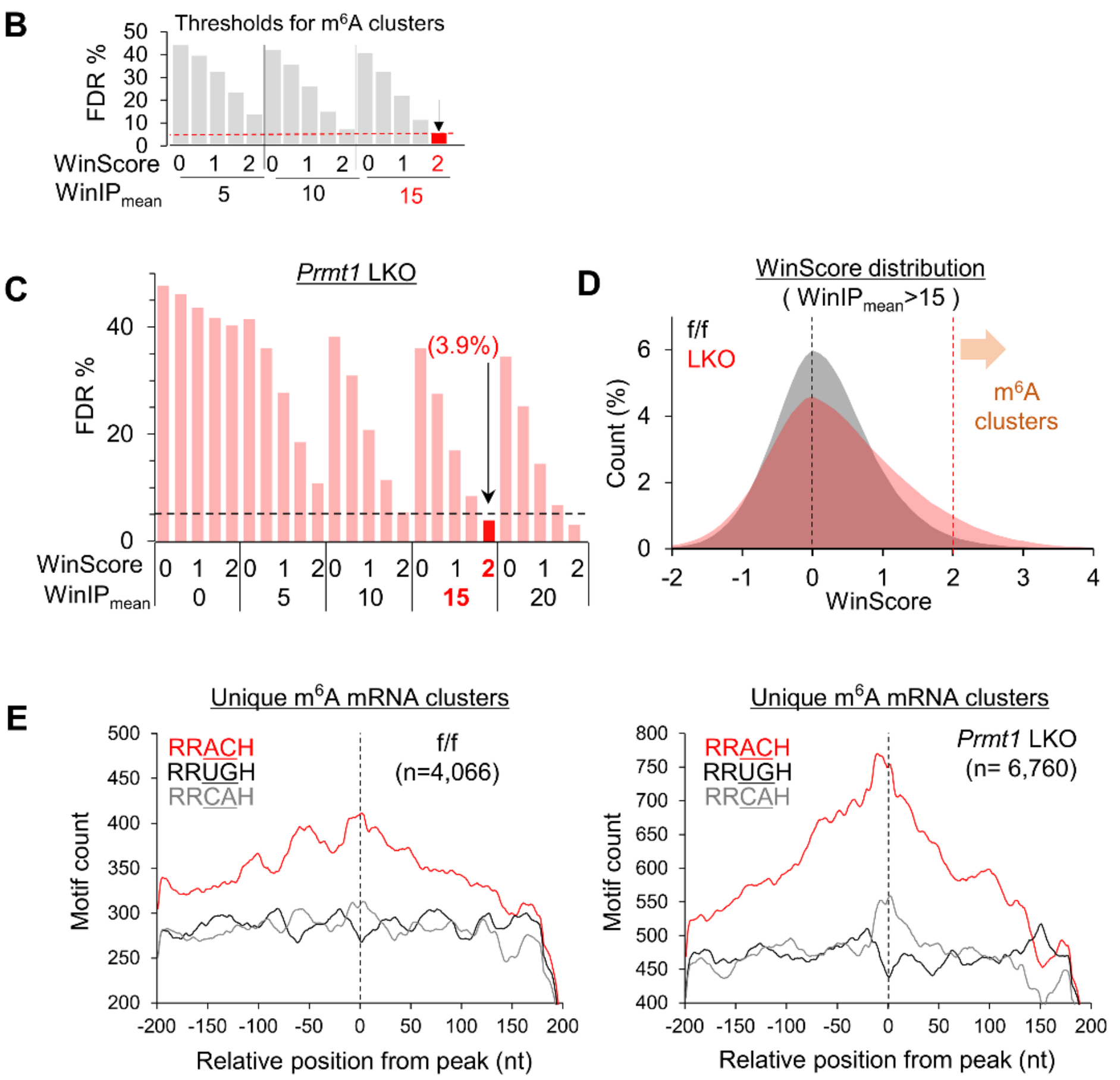

Figure S6. Analysis of m^{6}A clusters in the livers of *Prmt1* LKO and *Prmt1* f/f mice.
A. Mapping summary of m^{6}A RIP-Seq obtained from the livers of *Prmt1* LKO and *Prmt1* f/f mice. The mapped reads, generated using Bowtie2 program with mouse transcriptome (RefSeqSelect, derived from mm10 in UCSC genome browser), were filtered to retain only those presented in reproducible clusters using the CLIPick program *(92)* (biological complexity (BC) ≥ 2; upper panel). After calculating coverage, enriched clusters in m^{6}A IP were selected by comparing read distribution in input control. "m^{6}A cluster" was defined by merging m^{6}A enriched windows considering overlaps, and selecting ones located in

expressed mRNAs($\log_2(RPKM_{mean}) > 0$; “m^{6}A mRNA cluster”). To characterize m^{6}A clusters, "unique m^{6}A mRNA cluster" was defined as clusters with no other clusters within ± 200 nucleotides (nt), and "m^{6}A stop cluster" was selected by m^{6}A clusters located within ± 300 nt from the stop codon (lower panel). **B-D.** Distribution of false discovery rate (FDR, %; **B**, *Prmt1* f/f; **C**, *Prmt1* LKO) and WinScore (**D**) of windows of 20 nt with 10 nt overlap; FDRs were estimated by comparing the number of selected windows exceeding a set of thresholds in $WinIP_{mean}$ (mean coverage for each window in m^{6}A IP) and WinScore (log ratio of normalized coverage between m^{6}A IP and input control; $WinScore = \log_2([WinIP_{mean} / GeneIP_{median}] / [WinControl_{mean} / GeneControl_{median}])$; $GeneIP_{median}$, median coverage value of IP; $WinControl_{mean}$, mean coverage for each window in input; $GeneControl_{median}$, median coverage value of IP. The windows with absolute m^{6}A enrichment ($WinIP_{mean} > 15$) and WinScore over thresholds (> 2) were selected as FDR > 0.05 for m^{6}A cluster (highlighted in red (**B**, **C**) or orange (**D**)). **E.** Enrichment of m^{6}A methylation motif (RRACH) comparing with control motif (RRUGH and RRCAH) in *Prmt1* f/f (left panel, n = 4,066) and *Prmt1* LKO mice (right panel, n = 6,760).

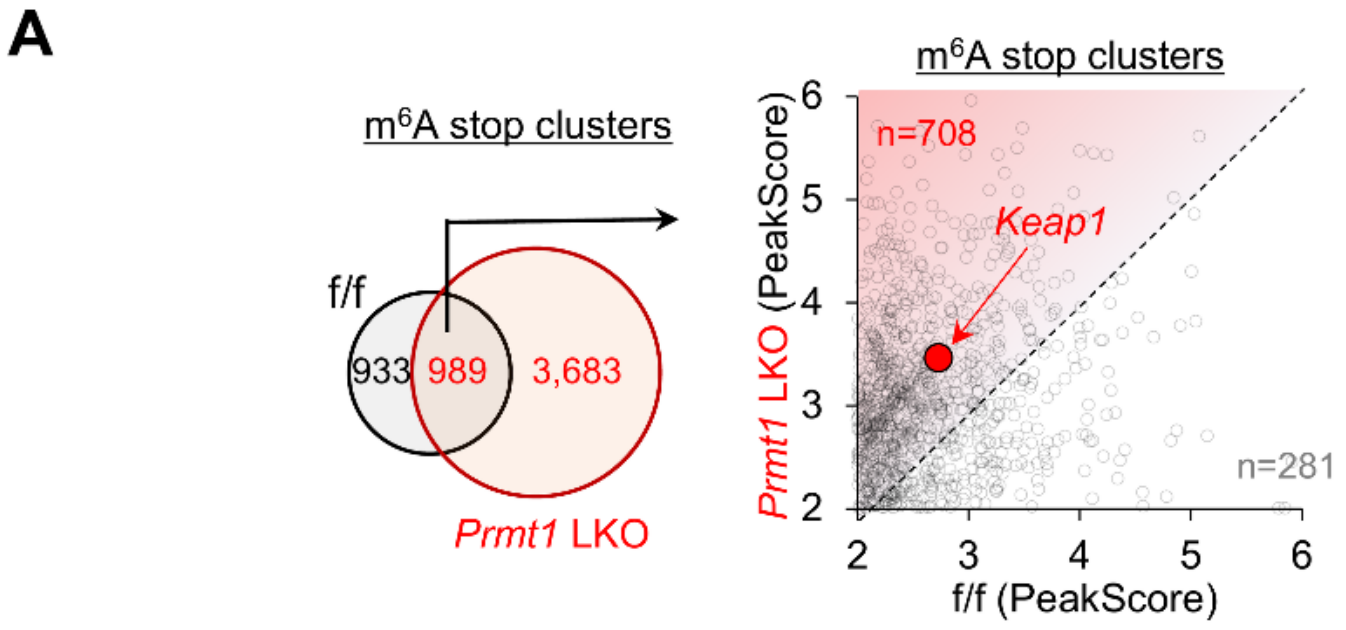

B

Sample name	Genotype	Sample type	Exp	Total read	Mapped read	%	IP cluster	m^6A cluster
WT	*Mettl14* +/+	Liver mRNA	Input	6,065,123	1,568,317	25.9	-	-
Mettl14$^{+/-}$	*Mettl14* +/-	Liver mRNA	Input	6,019,638	1,525,963	25.3	-	-
WT_IP	*Mettl14* +/+	Liver mRNA	m^6A IP	5,974,528	2,189,610	36.6	51,562	5,769
Mettl14$^{+/-}$_IP	*Mettl14* +/-	Liver mRNA	m^6A IP	7,627,824	2,943,460	38.6	53,697	6,169

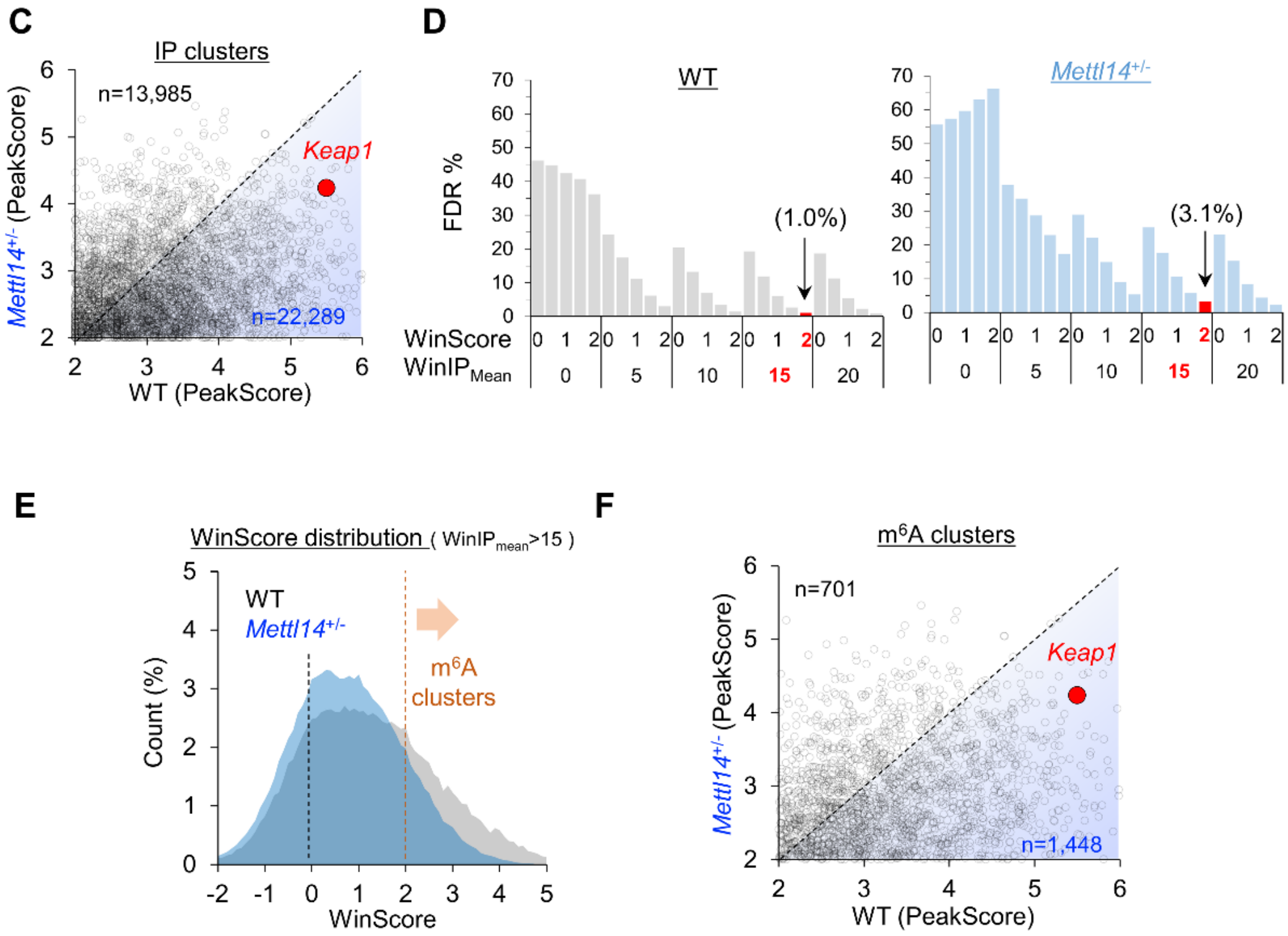

Figure S7. Analysis of m^6A clusters in the hepatocytes of *Mettl14*$^{+/-}$ and WT mice.
A. Differential analysis of m^6A stop clusters in *Prmt1* LKO and *Prmt1* f/f mice. The numbers of m^6A stop clusters in *Prmt1* LKO and *Prmt1* f/f mice were shown in the Venn diagram (left panel). Peak heights (PeakScore) of overlapping clusters between the two different genotypes were compared in the scatter plot (right panel); Enriched PeakScores in *Prmt1* LKO (n = 708) versus in *Prmt1* f/f (n = 281); *Keap1*, highlighted in red. **B.** Mapping summary of m^6A RIP-Seq, performed in hepatocytes of *Mettl14*$^{+/-}$ or wild type (WT) mice; IP cluster, compiled reads in m^6A IP; m^6A cluster, selected cluster considering reads from input control. **C.** Same comparative analysis for m^6A RIP-Seq results as in **A** except for IP clusters derived from *Mettl14*$^{+/-}$ versus WT; Reduced PeakScores in *Mettl14*$^{+/-}$ mice (n = 13,985) versus in WT mice (n = 22,289); *Keap1*, highlighted in red. **D, E.** Distribution of false discovery rate (FDR, %; **D**) and WinScore (**E**) as shown in Fig S6**B-D** except for results derived from

Mettl14$^{+/-}$ mice (**D**; right panel) and WT mice (**D**; left panel). Selected cutoffs were highlighted with red color in **D** and with orange dotted line in **E**. **F.** Same comparative analysis as in **C** except using the defined m^{6}A clusters in *Mettl14*$^{+/-}$ mice versus WT mice; Reduced PeakScores in *Mettl14*$^{+/-}$ mice (n = 701) versus in WT mice (n = 1,448); *Keap1*, highlighted in red.

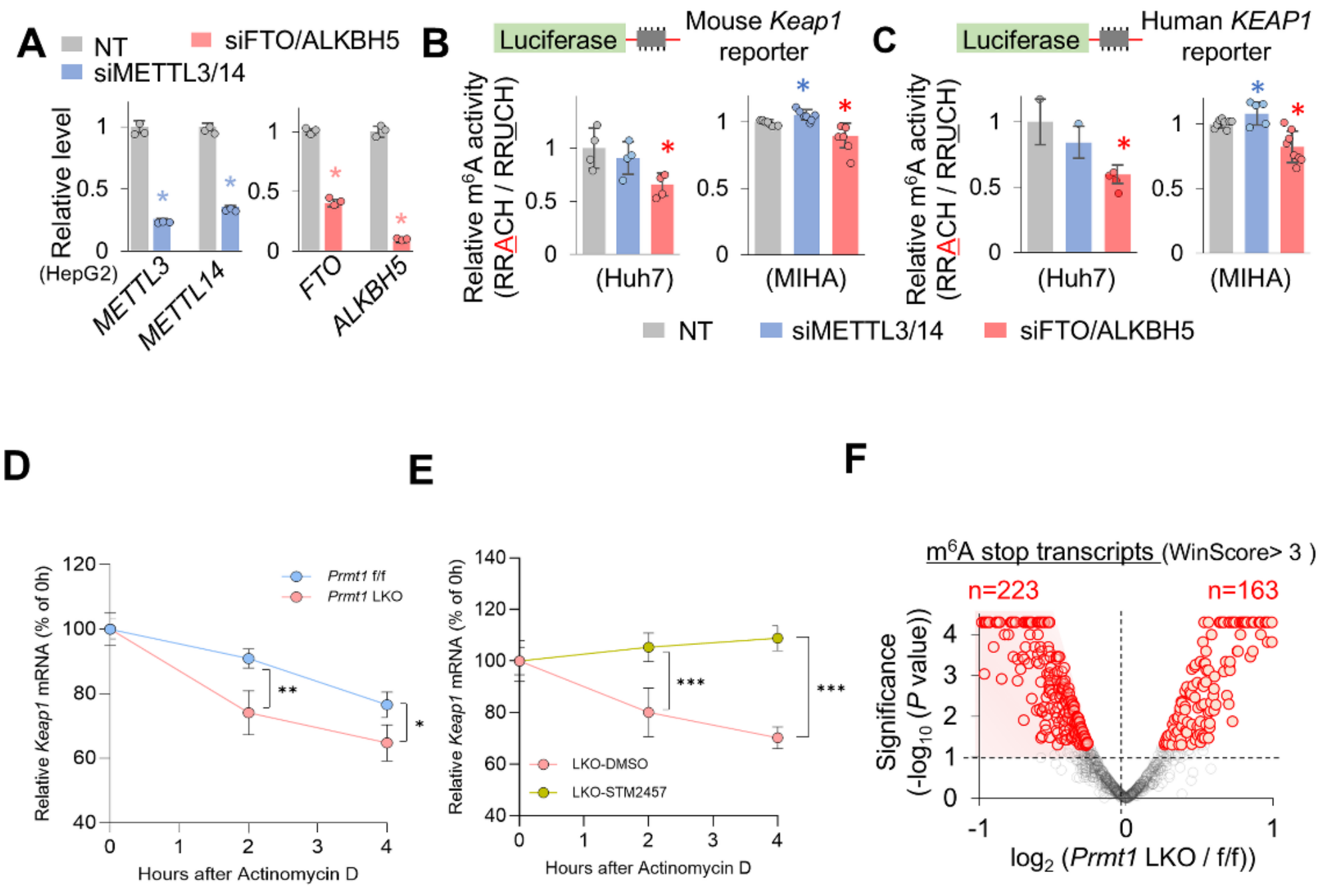

Figure S8. Validation of m^{6}A-dependent destabilization of *Keap1* mRNA.
A. Validation of knockdown efficiency of siRNAs targeting m^{6}A writers (METTL3 and METTL14; siMETTL3/14) and erasers (FTO and ALKBH5; siFTO/ALKBH5) in HepG2 cells using qRT-PCR; NT, control siRNA; *P = 1.2 x 10^{-5} (METTL3), 2.8 x 10^{-6} (METTL14), 1.5 x 10^{-5} (FTO), and 4.3 x 10^{-6} (ALKBH5), respectively (n = 3 per group). **B**, **C**. Luciferase reporter assays for the m^{6}A enriched region (indicated in Fig.4**G**) in mouse *Keap1* (**B**) and human *KEAP1* mRNA (**C**), following knock-down of m^{6}A writers (siMETTL3/14) or erasers (siFTO/ALKBH5) in Huh7 and MIHA cells. Relative m^{6}A activity is calculated for the repression-mediated by RRACH motifs, relative to by their mutant motifs, RRUCH; NT, control siRNA (n = 4); *P = 0.019 (n = 4), 0.006 (n = 7) and 0.012 (n = 8), respectively (**B**); *P = 0.005 (n = 4), 0.037 (n = 5) and 0.001 (n = 8), respectively (**C**). **D.** The mRNA levels of *Keap1* were measured by qRT-PCR in primary hepatocytes isolated from *Prmt1* f/f or *Prmt1* LKO mice treated with DMSO for 24 h, followed by Actinomycin D treatment (5 µM) for 0, 2, or 4 h. *Keap1* mRNA levels were normalized to the 0 h time point and are presented as a percentage of the initial value. Values represent the mean ± SEM (n = 3 per group). *P < 0.05 and **P < 0.01. **E.** The mRNA levels of *Keap1* were measured by qRT-PCR in primary hepatocytes isolated from *Prmt1* LKO mice treated with DMSO or STM2457 (20 µM) for 24 h, followed by Actinomycin D treatment (5 µM) for 0, 2, or 4 h. *Keap1* mRNA levels were normalized to the 0 h time point and are presented as a percentage of the initial value. Values represent the mean ± SEM (n = 3 per group). *P < 0.05, **P < 0.01, and ***P < 0.001. **F.** m^{6}A-modified mRNAs, that harbor m^{6}A stop clusters with height of WinScore > 3 in *Prmt1* LKO mice, were selected and analyzed for their differential expression in *Prmt1* LKO mice using RNA-Seq data, represented in Volcano plot as a subset of the results in Fig S2**B**; upregulated DEGs (right, red dot; n = 163) and downregulated DEGs (left, red dot with red shade; n = 223), P < 0.05 (>1.3 in y axis, dotted line).

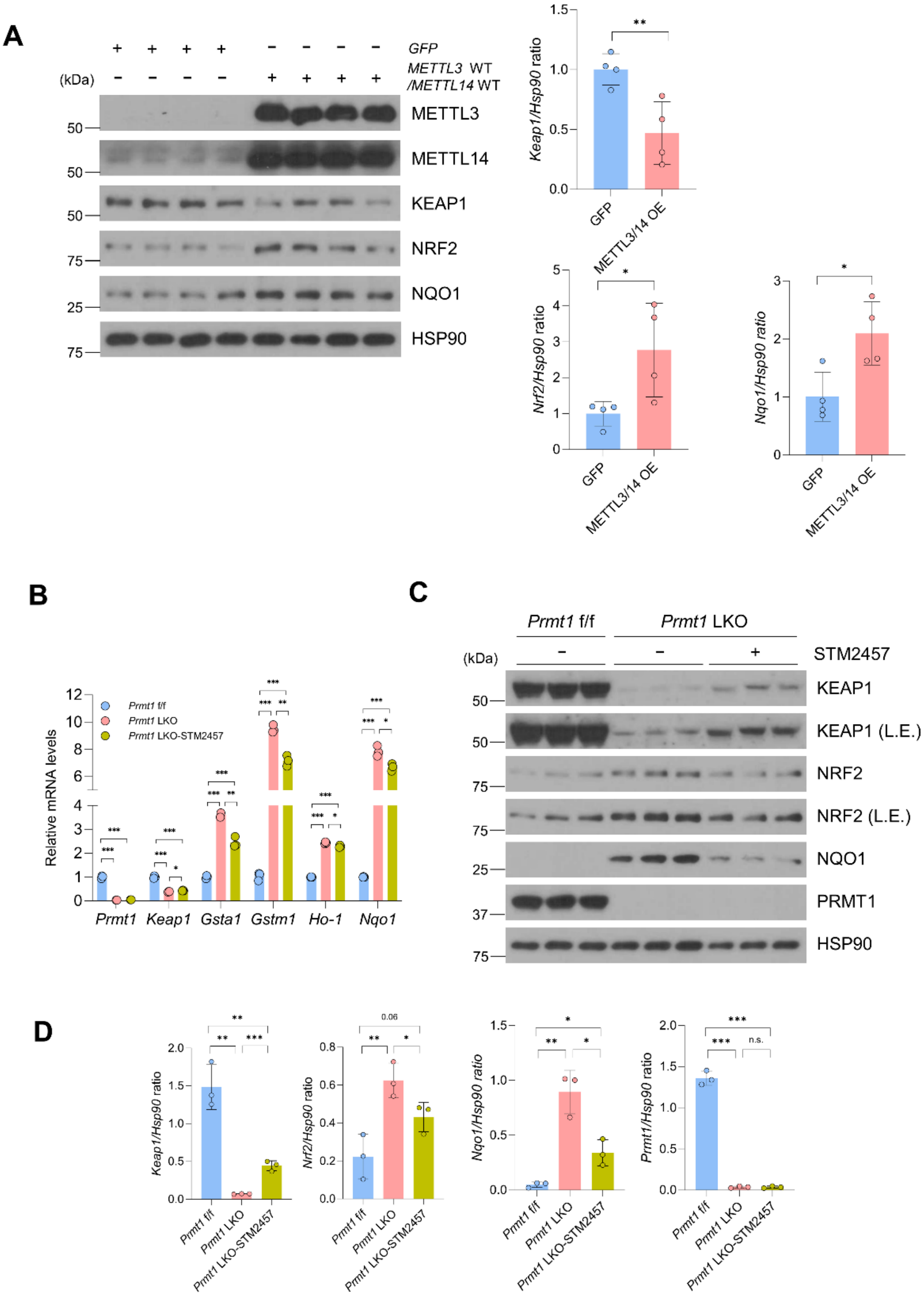

Figure S9. A role of METTL3/14 m6A writer on the control of KEAP1-NRF2 axis in hepatocytes.

A. Western blot analysis showing expression of METTL3, METTL14, KEAP1, NRF2 and its downstream target in mouse primary hepatocytes infected with Ad-GFP, Ad-WT METTL3 and Ad-WT METTL14 (left), and quantitation of each band (right) ($n = 4$ per group). **B**. The mRNA levels of *Prmt1*, *Keap1*, NRF2 target genes were measured in mouse primary hepatocytes from *Prmt1* f/f or *Prmt1* LKO mice treated with DMSO, STM2457 (20 μM, 24h). **C.** Western blot analysis showing expression of PRMT1, KEAP1, NRF2 and its downstream target in mouse primary hepatocytes from *Prmt1* f/f or *Prmt1* LKO mice treated with DMSO, STM2457 (20 μM, 72h). **D.** Quantitation of each band in Fig S9**C** ($n = 3$ per group). Values in **A**, **B**, and **D** represent mean ± SEM. * $P < 0.05$, ** $P < 0.01$ and ***$P < 0.001$. n.s.: not significant.

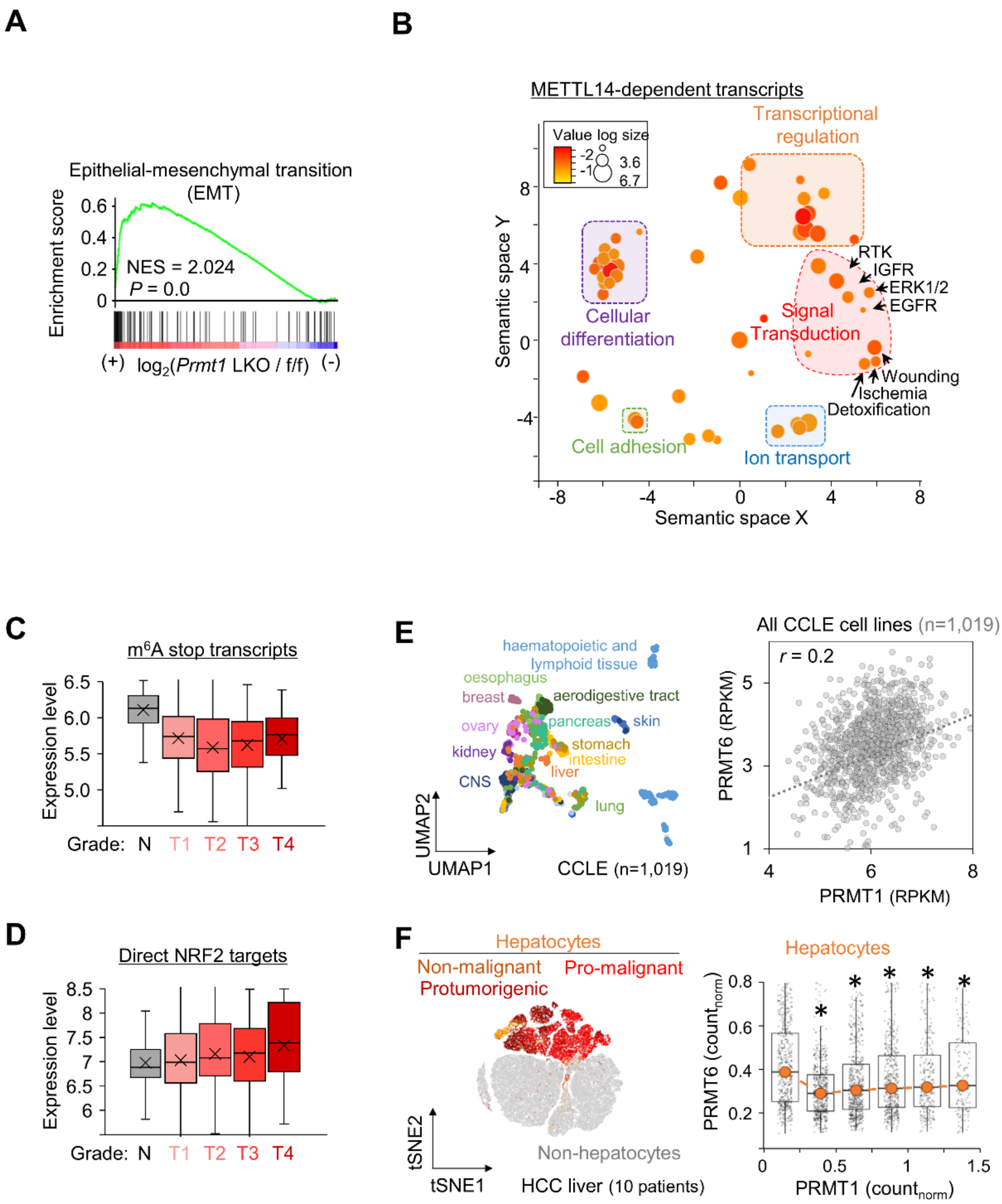

Figure S10. Association of m^{6}A stop transcripts in *Prmt1* LKO mice with NRF2 targets and human liver cancer.

A. GSEA results with "Epithelial-mesenchymal transition (EMT)" term in MSigDB, represented as distribution of enrichment score depending on fold change (log$_2$ (*Prmt1* LKO / f/f)); Normalized enrichment score (NES) and nominal *P* value indicate significant positive correlation (*P* = 0.0, NES > 1). **B.** GO analysis as indicated in Fig 5**A** except for METTL14-dependent transcripts (ΔPeakScore (*Mettl14*$^{+/-}$ - WT) < 0); semantic space as cluster representatives; the color and proportional size of circles indicate log^{10}(*P* value); *P* values were obtained using DAVID. **C**, **D**. Average expression levels of m^{6}A transcripts (**C**) and direct NRF2 targets (**D**) in HCC patients (LIHC in TCGA) across different tumor grades (normal tissues, N; T1, T2, T3 and T4 grades), represented as box plots. **E.** UMAP analyses for cancer cell lines (*n* = 1,019) based on gene expression profiles in CCLE (Cancer Cell Line Encyclopedia; left panel); comparison of expression (RPKM) between *PRMT1* and *PRMT6* in all cancer cell lines in CCLE (dotted line for linear regression; *r*, correlation coefficient; right panel). **F.** Hepatocyte population (tSNE visualization; left panel), identified by scRNA-

Seq analyses in HCC patients (*n* = 10; GSE149614) was subjected regardless of types (non-malignant, pro-malignant and pro-tumorigenic) to compare expression between *PRMT1* and *PRMT6* (normalized read-count, $count_{norm}$; right panel); *$P = 2.3 \times 10^{-47}$ ($n = 538$), 3.5×10^{-28} ($n = 588$), 4.5×10^{-17} ($n = 484$), 1.2×10^{-10} ($n = 270$) and 0.01 ($n = 187$), respectively; relative to cells with $count_{norm} < 0.25$ ($n = 643$). *P* values were from two-sided t-tests and repeated with biologically independent samples ($n \geq 3$); data are presented as mean ± SD.

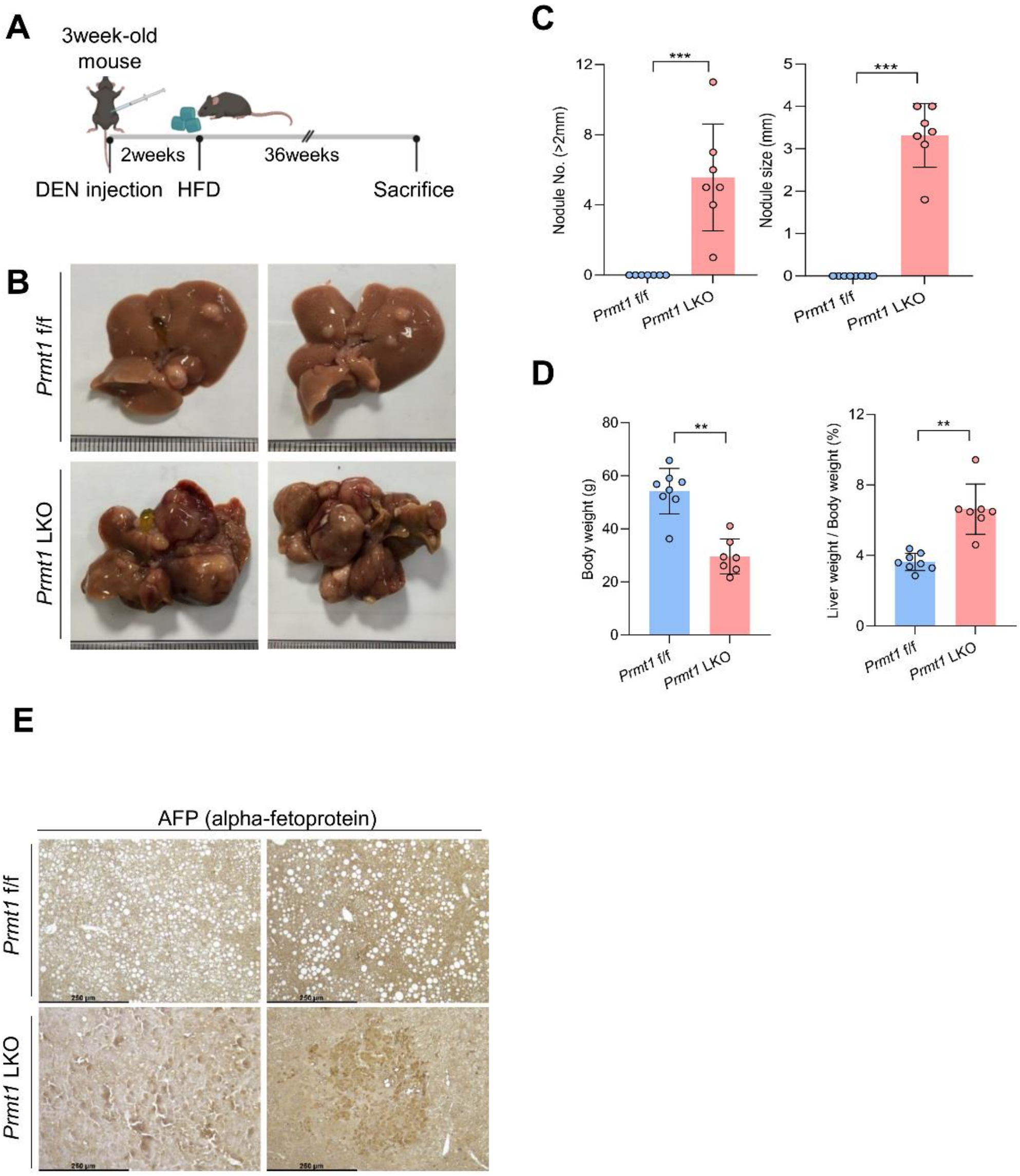

Figure S11. Depletion of *Prmt1* accelerates MASLD-associated HCC.
A. Schematic diagram for the generation of DEN-induced HCC in mice. **B.** A representative picture showing morphologies of livers from *Prmt1* f/f or *Prmt1* LKO mice that were injected with DEN, followed by HFD feeding for 38 weeks. **C.** Tumor number (left) and maximal size of tumors (right) of livers from *Prmt1* f/f or *Prmt1* LKO mice that were injected with DEN, followed by HFD feeding for 36 weeks (n = 5 for *Prmt1* f/f mice and n = 7 for *Prmt1* LKO mice). Values represent mean ± SEM. *$P < 0.05$ and ** $P < 0.01$. **D.** Body weight (left) and liver weight (right) of *Prmt1* f/f or *Prmt1* LKO mice that were injected with DEN, followed by HFD feeding for 36 weeks (n = 5 for *Prmt1* f/f mice and n = 7 for *Prmt1* LKO mice). Values represent mean ± SEM. ** $P < 0.01$. **E.** AFP staining for detection of hepatocellular carcinomas of liver tissues from *Prmt1* f/f or *Prmt1* LKO mice that were injected with DEN, followed by HFD feeding for 36 weeks. Data represent three independent experiments (n = 3 mice per group). Scale bar: 250 µm (x50). Fig. S11**A**: Created in BioRender. Kim, S. (2026) https://BioRender.com/o2vy791.

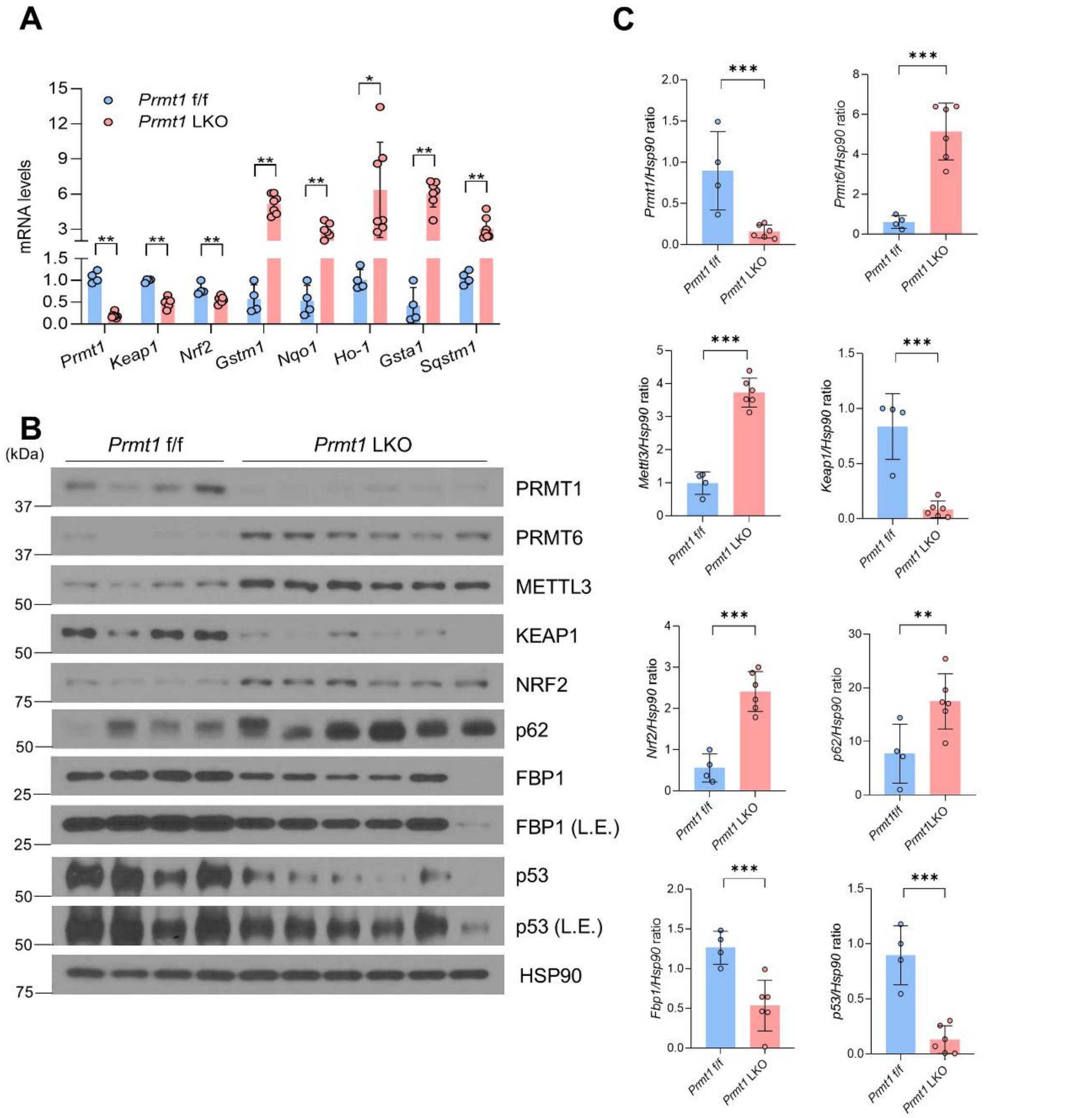

Figure S12. Depletion of *Prmt1* accelerates MASLD-associated HCC via METTL3-NRF2 pathway.

A. The mRNA levels of NRF2 target genes were measured by quantitative RT-PCR in the liver tissues from *Prmt1* f/f or *Prmt1* LKO mice that were injected with DEN, followed by HFD feeding for 36 weeks. (n = 5 for *Prmt1* f/f mice and n = 7 for *Prmt1* LKO mice). Values represent mean ± SEM. *$P < 0.05$ and ** $P < 0.01$. **B.** Western blot analysis showing expression of PRMTs, KEAP1, FBP1, p53, NRF2 and its downstream target proteins from livers from *Prmt1* f/f or *Prmt1* LKO mice that were injected with DEN, followed by HFD feeding for 36 weeks. **C.** Quantitation of each band shown in Fig S12**B** (n = 4 for *Prmt1* f/f mice and n = 6 for *Prmt1* LKO mice). Values represent mean ± SEM. ** $P < 0.01$ and ***$P <$ 0.001.

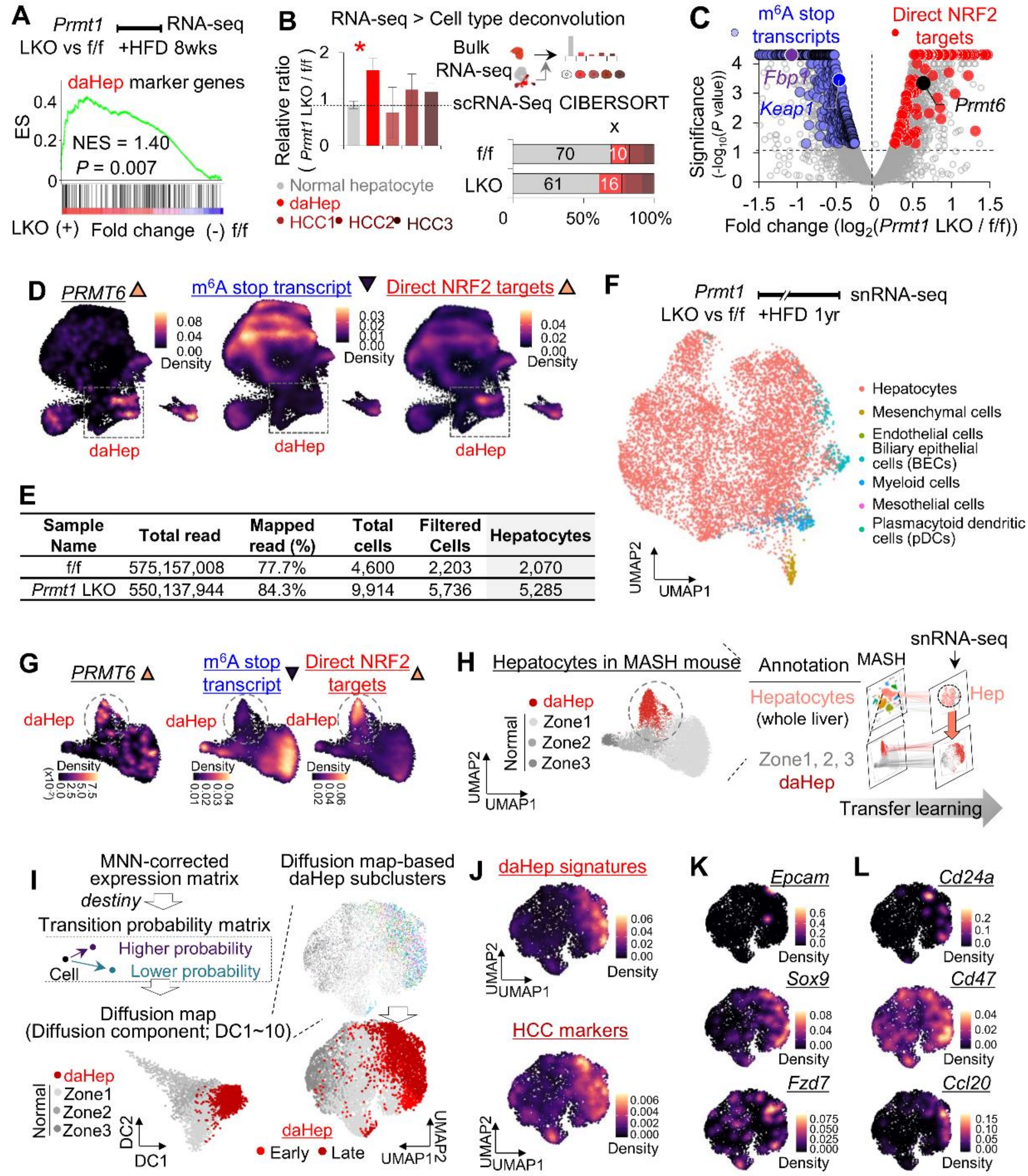

Figure S13. *Prmt1* deficiency associates with MASLD-induced daHep expansion in human and mouse.

A. GSEA result with daHep marker genes (n = 196) *(12)* for bulk RNA-Seq of *Prmt1* LKO mice, represented as distribution of enrichment score (ES) depending on fold change ($\log_2$ (*Prmt1* LKO / *Prmt1* f/f)). Normalized ES (NES) and nominal P value indicate significant positive correlation (P = 0.007, NES = 1.40). **B**. Deconvoluted bulk RNA-Seq results of *Prmt1* LKO based on snRNA-Seq results in Fig 1A and B (CIBERSORTx *(56)*), representing distribution of hepatocyte populations in *Prmt1* LKO compared to *Prmt1* f/f (right panel); relative ratio of cell population between *Prmt1* LKO mice and *Prmt1* f/f mice (left panel); *P = 0.021 (n = 3). **C.** Volcano plot for RNA-Seq results of *Prmt1* LKO liver relative to *Prmt1* f/f liver (n = 3 individual samples), representing global downregulation of m^6A stop transcripts (faint blue circle) and upregulation of direct NRF2 targets (red circle) with statistical significance (derived from Cuffdiff; P < 0.05); *Prmt6*, black circle; *Fbp1*, purple circle. **D.** Density plots for analyzing relative expression of *PRMT6* (left panel), m^6A stop transcripts (middle panel), direct NRF2 targets (right panel) in hepatocyte populations of liver diseases,

represented on UMAP results in Fig 1A and B. **E.** Summary of mapping results for snRNA-Seq, performed in livers from *Prmt1* f/f and LKO mice after 1-year HFD feeding. **F–H.** Reference-based annotation of cell populations by sequential anchor-based label transfer using a previously generated mouse MASH liver snRNA-seq dataset. Hepatocytes were first identified from whole-liver snRNA-seq data by transfer of liver cell type annotations (**F**). Density plots for *Prmt6*, m^6A stop transcripts and direct NRF2 targets in MASH mouse hepatocyte populations (**G**) recapitulated the trends observed in hepatocyte populations from human liver disease patients shown in **D**, supporting the hepatocyte subpopulation annotations for transfer learning (**H**, right panel). A second transfer was then performed to annotate hepatocyte subpopulations as zone 1 (light grey), zone 2 (grey), zone 3 (dark grey) hepatocytes and daHep (dark red) (**H**, left panel). **I,** Diffusion map-based pseudotime analysis was performed using the *destiny* package, and the diffusion map was visualized by diffusion component 1 (DC1) and DC2 (left panel), as shown in Fig. 7E. For daHep subclustering, a shared nearest-neighbor graph was constructed from DC1–DC10 and clusters were identified by Louvain clustering (upper right panel), followed by annotation of daHep subclusters as early daHep or late daHep (lower right panel) based on the expression of markers for daHep (*Anxa2, Gsta1*)*(10)*, early HCC (*Sqstm1, Gpc3* and *Cap2*) *(60, 61)* and late HCC (*Spp1, Myc* and *Cd44*) *(10, 62)*, as shown in Fig. 7I. **J–L.** Relative expression of daHep signatures (**J**, upper panel) *(12)*, HCC markers (**J**, lower panel) *(10, 106),* and stemness-related (**K**) *(63, 64)* and immunosuppression-related (**L**) *(55, 65, 66)* genes were shown as density plots on UMAP.

Table S1. RT-PCR primer list

Gene	Species	Forward Primer (5'-3')	Reverse Primer (5'-3')
Prmt1	m	ACCCTCACATACCGCAACTC	TGTTGGCTTTGACAATCTTCAC
Nrf2	m	CTCGCTGGAAAAAGAAGTGG	CCGTCCAGGAGTTCAGAGAG
Gstm1	m	AGAACCAGGTCATGGACACC	ACTTGGGCTCAAACATACGG
Gsta1	m	AAGACTGCCTTGGCAAAAGA	GAGGCTGCTGATTCTGCTCT
Gclc	m	AGGCTCTCTGCACCATCACT	TGGCACATTGATGACAACCT
Gpx2	m	GGCTTACATTGCCAAGTCGT	CCGGGTAGTTGTTCCTCAGA
Nqo1	m	TTCTCTGGCCGATTCAGAGT	GGCTGCTTGGAGCAAAATAG
Ho-1	m	CACGCATATACCCGCTACCT	CCAGAGTGTTCATTCGAGCA
Sqstm1	m	AGGGAACACAGCAAGCTCAT	GACTCAGCTGTAGGGCAAGG
Mettl3	m	CAAGCTGCACTTCAGACGAA	GCTTGGCGTGTGGTCTTT
Mettl14	m	AGAAACTTGCAGGGCTTCCT	TCTTCTTCATATGGCAAATTTTCTT
Fto	m	GACCTGTCCACCAGATTTTCA	AGCAGAGCAGCATACAACGTA
Alkbh5	m	ACTGAGCACAGTCACGCTTCC	GCCGTCATCAACGACTACCAG
Keap1	m	ATGGCCACACTTTTCTGGAC	ATCAATTTGCTTCCGACAGG
F4/80	m	TTTTCAGATCCTTGGCCATC	AGGAGCCTGGTACATTGGTG
Tnfa	m	CCCACACCGTCAGCCGATTT	GTCTAAGTACTTGGGCAGATTGAC C
Cd68	m	TTCTGCTGTGGAAATGCAAG	AGAGGGGCTGGTAGGTTGAT
Il1b	m	TGAAATGCCACCTTTTGACA	CTTCTCCACAGCCACAATGA
Timp1	m	TCCCCAGAAATCAACGAGAC	CTCAGAGTACGCCAGGGAAC
Acta2	m	CTGACAGAGGCACCACTGAA	CATCTCCAGAGTCCAGCACA
Mcp1	m	TCTGGACCCATTCCTTCT	ATCCCAATGAGTAGGCTG

L32	m,r,h	GGCCTCTGGTGAAGCCCAAGATCG	CCTCTGGGTTTCCGCCAGTTTCGC
Gapdh	m	TGCACCACCAACTGCTTAGC	GGCATGGACTGTGGTCATGAG

Gene	Species	Forward Primer (5'-3')	Reverse Primer (5'-3')
Hnf4a	m,r	GGTCAAGCTACGAGGACAGC	ATGTACTTGGCCCACTCGAC
Apoa2	m	TGCTGGTCACCATCTGTAGC	TTGGCCTTCTCCATCAAATC
Apob	m	GGCACTGTGGGTCTGGAT	TTCTTCTCTGGAGGGGACTG
Ect2	m	GTTCCTCCAAAGCAGTCAGC	CCACGCTGTCCTTCTTCTTC
Akr1b7	m	GTGAAAGCTCTGGGCATCTC	GATGCCCTTGGATTGACAGT
